# Supplementary material for: Rapid systematic review on developing web-based interventions to support people affected by cancer
Source: BMJ Open. 2022 Sep 6;12(9):e062026. doi: 10.1136/bmjopen-2022-062026 (PMC9454073; doi:10.1136/bmjopen-2022-062026)
Supplement: Supplementary data [file bmjopen-2022-062026supp002.pdf]

Developing web-based interventions to support people living with and affected by cancer: a protocol for a rapid review

Dr David Nelson[1], Dr Samuel Cooke[2], Heidi Green[2], Kathie McPeake[3], Prof. Mark Gussy[1], Dr Ros Kane[2]

[1] Lincoln International Institute for Rural Health, University of Lincoln, UK.

[2] School of Health and Social Care, University of Lincoln, UK.

[3] NHS Lincolnshire Clinical Commissioning Group

Correspondence to Dr David Nelson; dnelson@lincoln.ac.uk

Abstract

Introduction: More people are living with and beyond cancer and digital interventions are increasingly being used to support them at all stages through their journey. This rapid review aims to systematically identify and explore the existing evidence that reports on primary data concerning the development of web-based interventions used to support people living with and affected by cancer.

Methods and analysis: Keyword searches were performed in MEDLINE to identify peer-reviewed literature on web-based interventions that are designed to support people living with and affected by cancer. The review will include studies published in the English language and will not have any restrictions on publication date or geography. Screening and data extraction will be completed independently by two reviewers. The included studies will be tabulated and the results synthesised narratively.

Discussion: This rapid review aims to identify and synthesise the peer-reviewed academic literature that reports on primary data concerning the development of web-based interventions to support people living with and affected by cancer. This methodology was chosen to rapidly synthesise the existing peer-reviewed evidence to support the development and design of an online web-based platform that the team are working on to make qualitative research data on lived cancer experience publicly available and accessible.

Ethics and dissemination: The review was registered and given a favourable ethical opinion on the 19/07/21 by a committee at the University of Lincoln (Review ref: 2021_6976). The findings from this rapid review will be presented at appropriate conferences and published in a peer reviewed academic journal as well as a report for the National Institute for Health Research Clinical Research Network.

Systematic review registration: The protocol was registered on the Open Science Framework [insert link here].

Keywords: cancer survivorship; web-based support; user experience; rapid review; protocol

Introduction

Globally, cancer incidence is increasing with an estimated 18.1 million new diagnoses in 2018.[1] This can be attributed to advances in screening, earlier detection, diagnostic methods and improved treatments. Consequently, more and more people are now surviving cancer, and in the UK it is predicted that there will be four million people living with and beyond cancer by 2030.[2] Cancer raises a wide range of specific issues pertaining to information provision and emotional support [3] and there are now an increasing number of online health communities for people affected by cancer, each with their own specific aims.[4-6] Existing research has shown that people living with and affected by cancer use the internet for (1) content (online health information) (2) communication (e-mail and instant messaging) (3) communities (virtual support groups and forums) and (4) e-commerce (selling or buying products). [7]

The ongoing Covid-19 pandemic poses several challenges to oncology services and people living with and affected by cancer may now rely more heavily on digital and remote support.[8 9] Consequently, some psychosocial and supportive cancer care has now shifted from face-to-face to virtual delivery.[10] Digital health technologies have the potential to reduce health inequalities in cancer care and can improve access, integration and personalisation of care.[6] They can be particularly beneficial to those in rural and remote settings where access has long been acknowledged as a barrier to care.[11] However, the benefits of digital health technology depend partly on digital health literacy (capabilities and resources required by people to use and benefit from it).[6]

Existing reviews have previously explored the use of web-based interventions for supporting people living with and beyond cancer. These include identifying and evaluating the effectiveness of a wide range of web-based resources such as educational and psychosocial platforms[5 12 13], social media sites[14], mobile applications[14 15], and digital health interventions that focus on specific health behaviours e.g. physical activity and diet.[16] The current review will differ to previous reviews by exploring the academic evidence that reports on user and developer experience/perception for building and developing web-based tools. The evidence synthesised from the review will be used to directly inform the development of a novel web-based resource that will support those living with and beyond cancer through making qualitative research data on lived cancer experience publicly available and accessible.

This rapid review will aim to achieve the following:

- ❖ Identify and map the peer reviewed academic evidence that reports on primary data concerning the development and utilisation of web-based tools for supporting people living with and affected by cancer.

- ❖ Collate and analyse primary data with a view to informing evidence-based recommendations for the development of a novel and accessible web-based tool that meets the needs of people living with and affected by cancer.

Cooke S, *et al. BMJ Open* 2022; 12:e062026. doi: 10.1136/bmjopen-2022-062026

Methods

The team will utilise a rapid review approach which is now considered a key component of the knowledge synthesis family alongside systematic reviews, scoping reviews and realist reviews.[17] Despite the increase in popularity of rapid review methods there is still no universal agreement within the extant literature as to how a rapid review should be conducted or defined.[18-20] In sum, rapid reviews are a form of evidence synthesis in which components of the systematic review process are omitted or simplified with a view to producing findings in a timely manner. [18 19] Still, rapid reviews must remain systematic in their approach and have a duty to report the methods in a transparent manner making sure that they are clear about deviations or omissions from the Preferred Reporting Items for Systematic Reviews and Meta-Analyses (PRISMA) criteria.[21] This review will be conducted in line with the recently published guidance from the Cochrane Rapid Reviews Methods Group[22] and reported using the PRISMA checklist.[23]

Protocol and Ethics

The protocol has been registered on the Open Science Framework [insert link here]. This rapid review will be conducted over a 3-4 month period (July 2021 – October 2021).

The review was given a favourable ethical opinion by a committee at the University of Lincoln (Review ref: 2021_6976) on 19/07/21.

Search Strategy

We used keyword searches together with Boolean operators (OR and AND) and truncation (*) to locate relevant peer-reviewed literature on the user experience of web-based support that is delivered to people living with and affected by cancer. We searched MEDLINE (20/07/21) which is the leading full-text database of biomedical and health journals. The primary search strategy and syntax was developed and refined by three members of the review team (DN, SC, HG). The search strategy for MEDLINE can be found at Appendix 1. Due to time constraints, limited sources and the need to produce findings in a timely manner, we are limiting our search to one database which is considered acceptable for a rapid systematic review.[18 19] All database searches were supplemented with google scholar searches in addition with forward and backward citation searchers of relevant articles. Database searches will continually be updated to identify and incorporate the most up to date evidence where appropriate.

To identify people living with and affected by cancer we will use the following keywords: "cancer surviv*" or "living with cancer" or "living with and beyond cancer" or "cancer patient*" or "patients with cancer" "people affected by cancer" or "oncology patient" or "cancer experience*" or "cancer management" or "cancer support" or "cancer care*"

The following keywords will be used to identify web-based support and interventions: "web*" or "internet*" or "online*" or "digital*"

To search literature on user experience we will use the following keywords: "user experience*" or "usability" or "functionality" or "design" or "interaction" or "development" or "user testing"

Inclusion and Exclusion Criteria

Peer-reviewed publications will be selected for inclusion in the review utilising the pre-defined eligibility criteria outlined in Table 1 below.

Table 1 Inclusion and Exclusion Criteria

**Criterion**	**Include**	**Exclude**
Population	Adults (18+)<br>All genders<br>People living with cancer/caregivers/people affected by cancer<br>Any geography.	Non-adult populations (under 18).
Intervention	Website based/internet-based cancer support resources.	Support programmes that focus solely on mobile and digital apps.<br><br>E-learning programmes or interventions (self-directed and practitioner/professionally lead).<br><br>Social media/networking sites
Comparator	Not applicable.	Not applicable.
Outcomes	Reports primary data on user and developer experience/usability/functionality/design on web/internet-based support for people living with and affected by cancer.	There are no primary data reported on user experience/usability/functionality/design on web/internet-based support for people living with and affected by cancer.
Study design	Reports empirical research data using the following designs:<br>• Quantitative<br>• Qualitative<br>• Mixed Methods	• Systematic and literature reviews<br>• Editorials<br>• Commentaries<br>• Opinion pieces<br>• Case series or reports
Language	Published in the English language.	Not published in the English language.

Data and Analysis

Record Selection

References identified via the search were exported and managed using Endnote Version X9. The final search identified 2,452 articles for screening, see Appendix 1. The titles and abstracts will be independently screened against the eligibility criteria by two reviewers (DN and SC). Where discrepancies exist, the team will aim to resolve via discussion or through a third reviewer (HG). Following title and abstract screening, the remaining articles will be independently screened by full text, for inclusion by two reviewers (DN and SC), with any disagreements resolved through discussion or a third reviewer (RK). The results will be presented in a PRISMA flow diagram.

Data Extraction

Data will be extracted using an adapted Cochrane Data Extraction Template and this can be found at Appendix 2. This will be piloted with a subset ($n=5$) of full text studies that meet our eligibility criteria to determine whether any further changes are needed to the data extraction

template. After piloting the tool, two reviewers (DN and SC) will undertake data extraction for each full text article with cross checking for data quality taking place by a third reviewer (HG). Study characteristics will be extracted from each study based upon (1) study methods (e.g. aims/objectives, study design, participants, outcomes) (2) details on the web-based intervention/support and (3) study findings (details of all relevant data concerning user experience, usability, functionality, and design). All discrepancies will be resolved through further discussion, or where required, a fourth reviewer (RK).

Data synthesis and analysis

It is likely that the review will include a wide range of study designs that make use of both quantitative and qualitative methodologies. Following data extraction, the results of the full text articles will be tabulated. Quantitative data will be described using basic descriptive statistics as well as being written up narratively. We do not plan to conduct a formal statistical meta-analysis. Qualitative findings on user experience will be analysed using thematic synthesis.[24] Where possible we will group and comment on similarities and dissimilarities within the user experience data.

Quality assessment

The focus of this rapid review is on identifying and exploring the literature on user experience of web-based support that is delivered to people living with and beyond cancer, therefore, a quality assessment of included articles was not deemed appropriate.

Discussion

The aim of this research is to identify and map the peer reviewed academic evidence that reports on primary data concerning the development of web-based tools that support people living with and affected by cancer. It will also aim to collate and analyse data with a view to informing evidence-based recommendations for the development of a novel and accessible web-based tool that meets the needs of people living with and affected by cancer. It has been acknowledged that digital interventions have the potential to provide an excellent source of support for people living with and affected by cancer.[5 25] Specifically, they can help people to cope better with the disease and with side effects as well as improving self-management and wellbeing.[5]

A rapid review methodology was chosen to support and inform the timely need for the development and implementation of an innovative online web-based platform that is informed by peer-reviewed academic evidence. Rapid reviews are useful in adapting to and overcoming time and resource constraints that genuinely prevent the development and execution of a high-quality systematic review.[22] Nevertheless, researchers must endeavour to optimise the methodological rigour, clarity, and reproducibility of a rapid review, starting with the implementation of a rigorous and systematic protocol.[22]

Systematic reviews and meta-analyses are often intensive[26], especially when considering the large number of included studies, which is likely to be the case for evidence concerning user experience for developing web-based interventions in cancer. Whilst conducting a rapid review may accelerate the development process, it will enable a timely evidence appraisal that will address priority research questions as well as allowing for the rapid dissemination of findings.

Cooke S, *et al. BMJ Open* 2022; 12:e062026. doi: 10.1136/bmjopen-2022-062026

Dissemination

This rapid review and wider work (development of a web-based platform to support people living with and affected by cancer) benefits from the establishment of a study steering group with representation from academic researchers, cancer professionals and people with lived cancer experience. The review team will draw on their expertise and the findings will be disseminated in accordance with an ongoing dissemination strategy that will be developed collaboratively by the review team and steering group. This will involve presenting at appropriate local and national conferences, as well as, publishing in a peer reviewed academic journal. A summary of the findings will also be written up as a report for the National Institute for Health Research (NIHR) Clinical Research Network (CRN) who are funding this work. The team will disseminate the results in lay and accessible formats including using social media and press releases via the University of Lincoln and Macmillan Cancer Support.

Funding

This review was funded by the National Institute for Health Research (NIHR) Clinical Research Network (CRN).

Acknowledgements

We would like to acknowledge the Steering Group for their support in directing this review as well as the wider project. Acknowledgments must also go to Macmillan Cancer Support and the East Midlands Cancer Alliance for previous funding and their continued support throughout the early development of the Shared Lives Cancer project.

References

1. Bray F, Ferlay J, Soerjomataram I, et al. Global cancer statistics 2018: GLOBOCAN estimates of incidence and mortality worldwide for 36 cancers in 185 countries. *CA: A Cancer Journal for Clinicians* 2018;68(6):394-424. doi: https://doi.org/10.3322/caac.21492
2. Maddams J, Utley M, Møller H. Projections of cancer prevalence in the United Kingdom, 2010–2040. *British Journal of Cancer* 2012;107(7):1195-202. doi: 10.1038/bjc.2012.366
3. van Eenbergen MC, van de Poll-Franse LV, Heine P, et al. The Impact of Participation in Online Cancer Communities on Patient Reported Outcomes: Systematic Review. *JMIR Cancer* 2017;3(2):e15. doi: 10.2196/cancer.7312
4. van Eenbergen MC, van de Poll-Franse LV, Krahmer E, et al. Analysis of Content Shared in Online Cancer Communities: Systematic Review. *JMIR Cancer* 2018;4(1):e6. doi: 10.2196/cancer.7926
5. Escriva Boulley G, Leroy T, Bernetière C, et al. Digital health interventions to help living with cancer: A systematic review of participants' engagement and psychosocial effects. *Psycho-Oncology* 2018;27(12):2677-86. doi: https://doi.org/10.1002/pon.4867
6. Kemp E, Trigg J, Beatty L, et al. Health literacy, digital health literacy and the implementation of digital health technologies in cancer care: the need for a strategic approach. *Health Promot J Austr* 2021;32 Suppl 1:104-14. doi: 10.1002/hpja.387
7. Eysenbach G. The Impact of the Internet on Cancer Outcomes. *CA: A Cancer Journal for Clinicians* 2003;53(6):356-71. doi: https://doi.org/10.3322/canjclin.53.6.356
8. Al-Quteimat OM, Amer AM. The Impact of the COVID-19 Pandemic on Cancer Patients. *Am J Clin Oncol* 2020;43(6):452-55. doi: 10.1097/COC.0000000000000712
9. Burki TK. Cancer guidelines during the COVID-19 pandemic. *The Lancet Oncology* 2020;21(5):629-30. doi: 10.1016/S1470-2045(20)30217-5
10. Bultz BD, Watson L. Lessons learned about virtual cancer care and distress screening in the time of COVID-19. *Supportive Care in Cancer* 2021 doi: 10.1007/s00520-021-06322-6

11. Douthit N, Kiv S, Dwolatzky T, et al. Exposing some important barriers to health care access in the rural USA. *Public Health* 2015;129(6):611-20. doi: https://doi.org/10.1016/j.puhe.2015.04.001
12. Aapro M, Bossi P, Dasari A, et al. Digital health for optimal supportive care in oncology: benefits, limits, and future perspectives. *Support Care Cancer* 2020;28(10):4589-612. doi: 10.1007/s00520-020-05539-1 [published Online First: 2020/06/14]
13. McCann L, McMillan KA, Pugh G. Digital Interventions to Support Adolescents and Young Adults With Cancer: Systematic Review. *JMIR Cancer* 2019;5(2):e12071. doi: 10.2196/12071 [published Online First: 2019/08/02]
14. Charbonneau DH, Hightower S, Katz A, et al. Smartphone apps for cancer: A content analysis of the digital health marketplace. *DIGITAL HEALTH* 2020;6:2055207620905413. doi: 10.1177/2055207620905413
15. Prochaska JJ, Coughlin SS, Lyons EJ. Social Media and Mobile Technology for Cancer Prevention and Treatment. *Am Soc Clin Oncol Educ Book* 2017;37:128-37. doi: 10.1200/edbk_173841 [published Online First: 2017/06/01]
16. Roberts AL, Fisher A, Smith L, et al. Digital health behaviour change interventions targeting physical activity and diet in cancer survivors: a systematic review and meta-analysis. *J Cancer Surviv* 2017;11(6):704-19. doi: 10.1007/s11764-017-0632-1 [published Online First: 2017/08/06]
17. Moher D, Stewart L, Shekelle P. All in the Family: systematic reviews, rapid reviews, scoping reviews, realist reviews, and more. *Systematic Reviews* 2015;4(1):183. doi: 10.1186/s13643-015-0163-7
18. Khangura S, Konnyu K, Cushman R, et al. Evidence summaries: the evolution of a rapid review approach. *Systematic Reviews* 2012;1(1):10. doi: 10.1186/2046-4053-1-10
19. Tricco AC, Antony J, Zarin W, et al. A scoping review of rapid review methods. *BMC Medicine* 2015;13(1):224. doi: 10.1186/s12916-015-0465-6
20. Kelly SE, Moher D, Clifford TJ. DEFINING RAPID REVIEWS: A MODIFIED DELPHI CONSENSUS APPROACH. *International Journal of Technology Assessment in Health Care* 2016;32(4):265-75. doi: 10.1017/S0266462316000489 [published Online First: 2016/10/11]
21. Schünemann HJ, Moja L. Reviews: Rapid! Rapid! Rapid! …and systematic. *Systematic Reviews* 2015;4(1):4. doi: 10.1186/2046-4053-4-4
22. Garritty C, Gartlehner G, Nussbaumer-Streit B, et al. Cochrane Rapid Reviews Methods Group offers evidence-informed guidance to conduct rapid reviews. *Journal of Clinical Epidemiology* 2021;130:13-22. doi: https://doi.org/10.1016/j.jclinepi.2020.10.007
23. Moher D, Liberati A, Tetzlaff J, et al. Preferred Reporting Items for Systematic Reviews and Meta-Analyses: The PRISMA Statement. *PLOS Medicine* 2009;6(7):e1000097. doi: 10.1371/journal.pmed.1000097
24. Thomas J, Harden A. Methods for the thematic synthesis of qualitative research in systematic reviews. *BMC Medical Research Methodology* 2008;8(1):45. doi: 10.1186/1471-2288-8-45
25. Laing CM, Moules NJ, Sinclair S, et al. Digital Storytelling as a Psychosocial Tool for Adult Cancer Survivors. *Oncol Nurs Forum* 2019;46(2):147-54. doi: 10.1188/19.onf.147-154
26. Allen IE, Olkin I. Estimating time to conduct a meta-analysis from number of citations retrieved. *Jama* 1999;282(7):634-5. doi: 10.1001/jama.282.7.634 [published Online First: 1999/10/12]

Appendix 1 Search Strategy for MEDLINE

Key search terms	Date	Hits (*n*=)	Parameters
S1: “Cancer surviv*”	20/07/21	27,751	Medline only
S2: “Living with cancer”	20/07/21	865	Medline only
S3: “Living with and beyond cancer”	20/07/21	103	Medline only
S4: “Cancer patient*”	20/07/21	203,503	Medline only
S5: “Patients with cancer”	20/07/21	31,724	Medline only
S6: “People affected by cancer”	20/07/21	146	Medline only
S7: “Oncology patient*”	20/07/21	6,036	Medline only
S8: “Cancer experience*”	20/07/21	2,911	Medline only
S9: “Cancer management”	20/07/21	4,928	Medline only
S10: “Cancer support”	20/07/21	1,350	Medline only
S11: “Cancer care*”	20/07/21	26,976	Medline only
**S12: S1 OR S2 OR S3 OR S4 OR S5 OR S6 OR S7 OR S8 OR S9 OR S10 OR S11**	**20/07/21**	**280,235**	**Medline only**
S13: “Web*”	20/07/21	287,832	Medline only
S14: “Internet*”	20/07/21	113,871	Medline only
S15: “Online*”	20/07/21	150,659	Medline only
S16: “Digital*”	20/07/21	182,713	Medline only
**S17: S13 OR S14 OR S15 OR S16**	**20/07/21**	**650,510**	**Medline only**
S18: “User experience*”	20/07/21	2,428	Medline only
S19: “Usability”	20/07/21	15,671	Medline only
S20: “Functionality”	20/07/21	66,353	Medline only
S21: “Design”	20/07/21	1,135,342	Medline only
S22: “Interaction”	20/07/21	876,907	Medline only
S23: “Development”	20/07/21	3,148,188	Medline only
S24: “User testing”	20/07/21	350	Medline only
**S25: S18 OR S19 OR S20 OR S21 OR S22 OR S23 OR S24**	**20/07/21**	**4,858,310**	**Medline only**
**S26: S12 AND S17 AND S25**	**02/08/21**	**2,452**	**Medline only**

Appendix 2 Adapted Data Extraction Form

Review title or ID	
Study ID *(surname of first author and year first full report of study was published e.g. Smith 2001)*	
Report ID	
Report ID of other reports of this study including errata or retractions	
Notes	

General Information

Date form completed *(dd/mm/yyyy)*	
Name/ID of person extracting data	
Reference citation	
Study author contact details	
Publication type *(e.g. full report, abstract, letter)*	
Notes:	

Study eligibility

**Study Characteristics**	**Eligibility criteria** *(Insert inclusion criteria for each characteristic as defined in the Protocol)*	**Eligibility criteria met?** **Yes**	**No**	**Unclear**	**Location in text or source** *(pg & ¶/fig/table/other)*
**Type of study**	Quantitative design	☐	☐	☐	
	Qualitative design	☐	☐	☐	
	Mixed methods design	☐	☐	☐	
	Other (Please specify)				
**Participants**	Cancer (Specify what type)	☐	☐	☐	
	Caregiver	☐	☐	☐	
	Friends/family	☐	☐	☐	
**Types of intervention**	Website/Internet-based cancer support programme	☐	☐	☐	

Cooke S, *et al. BMJ Open* 2022; 12:e062026. doi: 10.1136/bmjopen-2022-062026

**Type of data (quant and/or qual)**<br><br>(Reports primary data on any of the following)	User experience	☐ ☐ ☐	
	Usability	☐ ☐ ☐	
	Functionality	☐ ☐ ☐	
	Design on web	☐ ☐ ☐	
	Internet-based support living with and affected by cancer.	☐ ☐ ☐	
INCLUDE ☐		EXCLUDE ☐	
Reason for exclusion			
Notes:			

DO NOT PROCEED IF STUDY EXCLUDED FROM REVIEW

Characteristics of included studies

Methods

	**Descriptions as stated in report/paper**	**Location in text or source** *(pg & ¶/fig/table/other)*
**Aim of study** *(e.g. efficacy, equivalence, pragmatic)*		
**Participants** *(e.g. Type of cancer, caregiver role, family and friend's role)*		
**Design** *(e.g. parallel, crossover, non-RCT, exploratory)*		

**Outcomes** *(details of primary data e.g. user experience, usability, functionality, design etc)*			
**Start/End date**			
**Ethical approval needed/ obtained for study**	☐ Yes ☐ No ☐ Unclear		
**Notes:**			

Cooke S, *et al. BMJ Open* 2022; 12:e062026. doi: 10.1136/bmjopen-2022-062026
