# Supplementary material for: Rapid systematic review on developing web-based interventions to support people affected by cancer
Source: BMJ Open. 2022 Sep 6;12(9):e062026. doi: 10.1136/bmjopen-2022-062026 (PMC9454073; doi:10.1136/bmjopen-2022-062026)
Supplement: Supplementary data [file bmjopen-2022-062026supp003.pdf]

Review title or ID	Hodgkin lymphoma survivor wellness: Development of a web-based intervention.
Study ID *(surname of first author and year first full report of study was published e.g. Smith 2001)*	Amweg et al. (2020)
Report ID	10.1188/20.CJON.284-289
Report ID of other reports of this study including errata or retractions	N/A
Notes: N/A	

General Information

Date form completed *(dd/mm/yyyy)*	12/10/2021
Name/ID of person extracting data	SC
Reference citation	Amweg LN, McReynolds J, Lansang K, Jones T, Snow C, Berry DL, Partridge AH, Underhill-Blazey ML. Hodgkin Lymphoma Survivor Wellness: Development of a Web-Based Intervention. Clin J Oncol Nurs. 2020 Jun 1;24(3):284-289. doi: 10.1188/20.CJON.284-289. PMID: 32441674.
Study author contact details	Could not access
Publication type *(e.g. full report, abstract, letter)*	Full report
Notes: N/A	

Study eligibility

**Study Characteristics**	**Eligibility criteria** *(Insert inclusion criteria for each characteristic as defined in the Protocol)*	**Eligibility criteria met?** **Yes**	**No**	**Unclear**	**Location in text or source** *(pg & ¶/fig/table/other)*
**Type of study**	Quantitative design	☐	☐	☐	
	Qualitative design	☐	☐	☐	
	Mixed methods design	☒	☐	☐	Page 1 – Abstract<br>Page 2 – methods – Focus groups – Development and usability testing
	Other (Please specify)				

**Participants**	Cancer (Specify what type)	☒	☐	☐	Page 2 – Participant identification and recruitment
	Caregiver	☐	☐	☐	
	Friends/family	☐	☐	☐	
**Types of intervention**	Website/Internet-based cancer support programme	☒	☐	☐	Page 2 – Design and setting

**Type of data (quant and/or qual)** (Reports primary data on any of the following)	User experience/Needs/Preferences	☒	☐	☐	Page 2 – Focus groups
	Usability/Acceptability/Feasibility	☒	☐	☐	Page 2 – Development and individual usability testing
	Functionality	☐	☐	☐	
	Design on web	☐	☐	☐	
	Internet-based support living with and affected by cancer.	☐	☐	☐	
INCLUDE ☒		EXCLUDE ☐			
Reason for exclusion					

Notes:

The aim of this article was to adapt and evaluate a previously developed survivorship care website for Hodgkin Lymphoma survivors.

A mixed methods design broken down into phases

1) Focus group interviews – user needs
2) Web design and user testing – usability

DO NOT PROCEED IF STUDY EXCLUDED FROM REVIEW

Characteristics of included studies

Methods

	Descriptions as stated in report/paper		Location in text or source *(pg & ¶/fig/table/other)*
**Aim of study** *(e.g. efficacy, equivalence, pragmatic)*	The aim of this article was to adapt and evaluate a previously developed survivorship care website for Hodgkin Lymphoma survivors.		Page 1 – Abstract<br><br>Page 2 – Introduction – paragraph 2
**Participants** *(e.g. Type of cancer, caregiver role, family and friend's role)*	Hodgekins Lymphoma survivors		Page 2 – Methods – Participant identification and recruitment
**Design** *(e.g. parallel, crossover, non-RCT, exploratory)*	Mixed methods user centred design – two phases<br><br>1) Focus groups<br>2) Development and individual usability testing		Page 2 – Methods – Focus groups – Development and usability testing
**Outcomes** *(details of primary data e.g. user experience, usability, functionality, design etc)*	User needs/preferences and usability of web-resource		Page 2 – Methods – Focus groups – Development and usability testing
**Start/End date**			
**Ethical approval needed/ obtained for study**	☒ Yes ☐ No ☐ Unclear	The Dana-Farber/Harvard Cancer Centre Institutional Review Board approved all study procedures. The study was conducted from April 2017 through December 2018.	Page 2 – Methods – Design and setting.

Notes:
