# Supplementary material for: Rapid systematic review on developing web-based interventions to support people affected by cancer
Source: BMJ Open. 2022 Sep 6;12(9):e062026. doi: 10.1136/bmjopen-2022-062026 (PMC9454073; doi:10.1136/bmjopen-2022-062026)
Supplement: Author's manuscript [file bmjopen-2022-062026.draft_revisions.pdf]

BMJ Open

BMJ Open is committed to open peer review. As part of this commitment we make the peer review history of every article we publish publicly available.

When an article is published we post the peer reviewers' comments and the authors' responses online. We also post the versions of the paper that were used during peer review. These are the versions that the peer review comments apply to.

The versions of the paper that follow are the versions that were submitted during the peer review process. They are not the versions of record or the final published versions. They should not be cited or distributed as the published version of this manuscript.

BMJ Open is an open access journal and the full, final, typeset and author-corrected version of record of the manuscript is available on our site with no access controls, subscription charges or pay-per-view fees (http://bmjopen.bmj.com).

If you have any questions on BMJ Open's open peer review process please email info.bmjopen@bmj.com

BMJ Open

# A rapid systematic review on developing web-based interventions to support people affected by cancer.

| | |
|---|---|
| Journal: | *BMJ Open* |
| Manuscript ID | bmjopen-2022-062026 |
| Article Type: | Original research |
| Date Submitted by the Author: | 16-Feb-2022 |
| Complete List of Authors: | Cooke, Samuel; University of Lincoln, School of Health and Social Care<br>Nelson, David; University of Lincoln, Lincoln International Institute for Rural Health; Macmillan Cancer Support<br>Green, Heidi; University of Lincoln, School of Health and Social Care<br>McPeake, Kathie; Macmillan Cancer Support; NHS Lincolnshire Clinical Commissioning Group<br>Gussy , Mark ; University of Lincoln, Lincoln International Institute for Rural Health<br>Kane, R; University of Lincoln, School of Health and Social Care |
| Keywords: | PUBLIC HEALTH, ONCOLOGY, Health informatics < BIOTECHNOLOGY & BIOINFORMATICS, World Wide Web technology < BIOTECHNOLOGY & BIOINFORMATICS |
| | |

SCHOLARONE™
Manuscripts

*I, the Submitting Author has the right to grant and does grant on behalf of all authors of the Work (as defined in the below author licence), an exclusive licence and/or a non-exclusive licence for contributions from authors who are: i) UK Crown employees; ii) where BMJ has agreed a CC-BY licence shall apply, and/or iii) in accordance with the terms applicable for US Federal Government officers or employees acting as part of their official duties; on a worldwide, perpetual, irrevocable, royalty-free basis to BMJ Publishing Group Ltd ("BMJ") its licensees and where the relevant Journal is co-owned by BMJ to the co-owners of the Journal, to publish the Work in this journal and any other BMJ products and to exploit all rights, as set out in our licence.*

*The Submitting Author accepts and understands that any supply made under these terms is made by BMJ to the Submitting Author unless you are acting as an employee on behalf of your employer or a postgraduate student of an affiliated institution which is paying any applicable article publishing charge ("APC") for Open Access articles. Where the Submitting Author wishes to make the Work available on an Open Access basis (and intends to pay the relevant APC), the terms of reuse of such Open Access shall be governed by a Creative Commons licence – details of these licences and which Creative Commons licence will apply to this Work are set out in our licence referred to above.*

*Other than as permitted in any relevant BMJ Author's Self Archiving Policies, I confirm this Work has not been accepted for publication elsewhere, is not being considered for publication elsewhere and does not duplicate material already published. I confirm all authors consent to publication of this Work and authorise the granting of this licence.*

For peer review only

# A rapid systematic review on developing web-based interventions to support people affected by cancer.

Samuel Cooke[1], David Nelson[2,3], Heidi Green[1], Kathie McPeake[3,4], Mark Gussy[2], Ros Kane[1]

[1] School of Health and Social Care, University of Lincoln, UK.

[2] Lincoln International Institute for Rural Health, University of Lincoln, UK.

[3] Macmillan Cancer Support, London, UK.

[4] NHS Lincolnshire Clinical Commissioning Group

Correspondence to Samuel Cooke, University of Lincoln, School of Health and Social Care, Brayford Way, Brayford Pool, Lincoln, LN6 7TS Email: scooke@lincoln.ac.uk

Word Count: 3770

For peer review only



For peer review only - http://bmjopen.bmj.com/site/about/guidelines.xhtml

# ABSTRACT

**Objective:** To systematically identify and explore the existing evidence to inform the development of web-based interventions to support people affected by cancer (PABC).

**Design:** A rapid review design was employed in accordance with the guidance produced by the Cochrane Rapid Reviews Methods Group and reported using the PRISMA checklist. A rapid review was chosen as there was a need for a timely evidence synthesis to underpin the subsequent development of an online digital resource (Shared Lives: Cancer) as part of an ongoing funded project.

**Methods and outcomes:** Keyword searches were performed in MEDLINE to identify peer-reviewed literature that reported primary data on the development of web-based interventions designed to support PABC. The review included peer reviewed studies published in the English language with no limits set on publication date or geography. Key outcomes included any primary data that reported on the design, usability, feasibility, acceptability, functionality, and user experience of web-based resource development.

**Results:** Ten studies were identified that met the pre-specified eligibility criteria. All studies employed an iterative, co-design approach underpinned by either quantitative, qualitative, or mixed methods. The findings from the ten articles were grouped into the following overarching themes (1) exploring current evidence, guidelines, and theory, (2) identifying user needs and preferences, and (3) evaluating the usability, feasibility, and acceptability of resources.

**Conclusion:** The findings of this rapid review provide novel methodological insights into the approaches used to design web-based interventions to support PABC. Our findings have the potential to inform and guide researchers when considering the development of future digital health resources.

**Trial registration:** The review protocol was registered on the Open Science Framework (osf.io/ucvsz).




For peer review only - http://bmjopen.bmj.com/site/about/guidelines.xhtml

## STRENGTHS AND LIMITATIONS OF THIS STUDY

- This rapid review provides original and important insights into the methodological approaches used to design and develop web-based resources to support PABC.

- This review provides a rapid, yet comprehensive synthesis of the current evidence to support the time-sensitive decision making for the development and implementation of a novel digital resource (Shared Lives: Cancer) to help support PABC.

- This rapid review, whilst streamlined, was conducted using a systematic methodology, following rigorous reporting guidelines to ensure transparency and reproducibility.

- Whilst considered a key part of the knowledge synthesis 'family', rapid review methods are not subject to the same robustness as a full systematic review and are more vulnerable to bias and error.

- Due to time constraints, database searches were restricted to one database only and no formal quality assessment was performed on the included studies.

For peer review only



For peer review only - http://bmjopen.bmj.com/site/about/guidelines.xhtml

# INTRODUCTION

Improvements in cancer screening, early detection, diagnostic methods, and treatment are resulting in an increasing number of people living with and beyond cancer.[1-4] Globally, there were an estimated 18.1 million new diagnoses in 2018.[5] In the UK, it is estimated that 4 million people will be living with and beyond cancer by 2030.[6] As services have expanded to support the continuing rise in cancer incidence, so too have the complexities in delivering care.[7-9] This is epitomised by the changes in the way cancer care has been implemented over recent decades, which in the UK for example, now involves a multitude of bodies responsible for purchasing, commissioning, delivering, and regulating services.[8,10]

To ensure the provision of future cancer services adapts to changes in health needs, medical advances, and societal developments, NHS England implemented a long-term plan in which digital health technologies are central.[11] Digital health technologies have become an important tool in cancer care with the potential to revolutionise patient data, transform patient experiences, improve patient recovery, and improve the access, integration, and personalisation of care.[9-12] Evidence suggests that individuals living with and beyond cancer are engaging with digital health technologies now more than ever[13-16] and are using them to frequently access online health information as well as virtual support groups and forums.[17,18]

The rapid growth of internet use has led to a substantial increase in the number of web-based interventions to support PABC, including a wide range of educational and psychosocial platforms,[19-21] social media sites,[22] mobile applications,[22,23] and digital health interventions that focus on specific health behaviours e.g. physical activity and diet.[24] Whilst previous reviews have focused predominantly on the evaluation of web-based interventions, there remains little evidence documenting the developmental (design, usability, feasibility, acceptability, functionality, and user experience) processes of web-based interventions in cancer populations. This review assumes a novel approach by exploring and synthesising the academic literature that reports on the development of web-based tools in cancer.



For peer review only - http://bmjopen.bmj.com/site/about/guidelines.xhtml

The findings will be used to directly inform the development of a novel web-based resource called (Shared Lives: Cancer),[25,26] that aims to support PABC through making qualitative research data on lived cancer experience publicly available and freely accessible.

This rapid review aims to:

- Identify and map the peer-reviewed academic evidence that reports primary data concerning the development of web-based interventions for supporting PABC.
- Collate and report on the academic evidence with a view to informing web-based interventions for supporting PABC.

## METHODS

This study used a rapid review approach adhering to the recently published guidance from the Cochrane Rapid Reviews Methods Group and for reporting used the Preferred Reporting Item for Systematic Reviews and Meta-Analyses (PRISMA) checklist, see supplementary material (S1). Rapid reviews are now considered a key component of the knowledge synthesis family alongside systematic reviews, scoping reviews and realist reviews. They provide a streamlined, efficient, and pragmatic approach to evidence synthesis.[27] In summary, rapid reviews are a form of evidence synthesis in which components of the systematic review process are simplified, with a view to producing findings in a timely manner.[28,29] Still, rapid reviews must remain systematic in their approach and have a duty to report their methods in a transparent manner making sure they are clear about deviations or omissions from the PRISMA criteria. This review was conducted over a 4-month period (July 2021 – October 2021). The study protocol has been registered on the Open Science Framework (osf.io/ucvsz) to promote reproducibility and facilitate methodological transparency, see supplementary material (S2).

### Ethics Approval

Not applicable/No human participants included.



For peer review only - http://bmjopen.bmj.com/site/about/guidelines.xhtml

**Patient and Public Involvement**

No patient involvement.

**Search Strategy**

Keyword searches together with Boolean operators (OR and AND) and truncation (*) were used to locate relevant peer-reviewed literature on the development of web-based support that is delivered to PABC. Due to the need to produce findings in a timely manner, database searches were limited to one database which is considered acceptable for a rapid systematic review. MEDLINE was searched as it is the leading full-text database of biomedical and health journals. The primary search strategy and syntax was developed and refined by three members of the review team (SC, DN, HG). All database searches were supplemented by Google Scholar searches in addition to forward and backward citation tracking on all relevant articles. Database searches were continually updated to identify and incorporate the most up to date evidence where appropriate.

To identify PABC the following keywords were used: "cancer surviv*" or "living with cancer" or "living with and beyond cancer" or "cancer patient*" or "patients with cancer" "people affected by cancer" or "oncology patient" or "cancer experience*" or "cancer management" or "cancer support" or "cancer care*". To identify web-based support and interventions the following keywords were used: "web*" or "internet*" or "online*" or "digital*". To search literature on user experience the following keywords were used: "user experience*" or "usability" or "functionality" or "design" or "interaction" or "development" or "user testing". The search strategy for MEDLINE can be found in supplementary material (S2).

All retrieved records were collated and stored using Endnote referencing software (EndNote X9, Clarivate Analytics, Philadelphia, USA). The titles and abstracts were screened against the eligibility criteria by one reviewer (SC). Identified discrepancies were resolved via discussion. Following title and abstract screening, the remaining articles were independently screened by full text, for inclusion by two reviewers (SC and DN), with any disagreements again resolved through discussion.



For peer review only - http://bmjopen.bmj.com/site/about/guidelines.xhtml

## Eligibility Criteria

Inclusion criteria

Peer-reviewed publications were selected for inclusion in this review if they met the following pre-defined eligibility based on the PICOT approach. **Population:** Adults (aged 18+), all genders, people living with cancer or affected by cancer, caregivers, any geographical location. **Intervention:** Website-based cancer support resources. **Comparator:** Not applicable. **Outcomes:** Reports primary data on the design, usability, feasibility, acceptability functionality, or user and developer experience of web-based support for PABC. **Type:** Reports empirical research data using either quantitative, qualitative, or mixed methods design. Only publications written in English language were included.

Exclusion criteria

Peer-reviewed publications were excluded based on the following exclusion criteria. **Population:** Non-adult population (under the age of 18). **Intervention:** Support programmes that focus solely on mobile and digital apps, E-learning programmes or interventions (self-directed and practitioner/professionally led), social media or networking sites. **Comparator:** Not applicable. **Outcomes:** No primary data reported on the design, usability, feasibility, acceptability, functionality, or user experiences of web-based support for PABC. **Type**: Systematic reviews or literature reviews, editorials, commentaries, opinion pieces, case series, or reports. Any publications that were not written in the English language were excluded from this review.

## Data abstraction

Data were extracted using an adapted Cochrane Data Extraction Template, see supplementary (S3). One reviewer (SC) undertook data extraction for each full text article with cross checking taking place by a second reviewer (DN). Study characteristics were extracted from each study based upon (1) study methods (e.g. aims/objectives, study design, participants, outcomes) (2) details on the web-based intervention/support and (3) study findings (details of all relevant data concerning user experience, needs, preferences, usability, acceptability ,feasibility, functionality, and design).



For peer review only - http://bmjopen.bmj.com/site/about/guidelines.xhtml

For peer review only

### Quality assessment

The focus of this rapid review is on identifying and exploring the literature on the development of web-based support that is delivered to PABC therefore, a quality assessment of included articles was not deemed appropriate.

### Data synthesis and analysis

The review included a wide range of study designs that utilised quantitative, qualitative, and mixed methodologies. To identify and map the evidence on the development of web-based interventions for supporting PABC we tabulated the results. This was then accompanied by a narrative summary where comments on the similarities and dissimilarities within data were made. Due to the wide heterogeneity of the design and outcomes of included studies, as well as the considerable amount of qualitative data, a formal statistical meta-analysis was not conducted; however, the findings were synthesised narratively.

## RESULTS

### Search results

The search of MEDLINE database provided a total of 2,446 distinct citations with an additional 6 identified through secondary sources, see Figure 1. After reviewing for title and abstract, 2,439 did not meet the pre-specified eligibility criteria. The remaining 13 citations were reviewed for full text and examined in detail for inclusion in this review. Three did not meet the pre-specified inclusion criteria for various reasons, see Figure 1. Overall, 10 studies met the pre-defined eligibility criteria that focused on the development of web-based tools to support PABC.

### Study Characteristics

The ten articles were published between 2012-2020 and were undertaken in Australia,[31] Belgium,[32] Vietnam,[33] United Kingdom,[34-37] and the United States of America.[38-40] Five studies focused on people with specific cancer types including survivors of Hodgkin Lymphoma,[38] patients with experience of gynaecological cancers,[34] survivors of oral cancer,[39] and patients and survivors of



For peer review only - http://bmjopen.bmj.com/site/about/guidelines.xhtml

breast cancer,[32,40] whilst four studies,[31,35,36] included patients with experience of a range of cancer types. Some studies also included family caregivers,[39] intimate partners,[32] healthcare professionals,[33,34,36,38] and researchers[34] alongside people with lived cancer experience. One of the included studies collected data with carers of people with cancer and health professionals only.[37]

All studies employed an iterative, co-designed methodological approach for the development of web-resources to support PABC. Two of the studies employed a mixed methods research design,[38,40] six utilised both quantitative and qualitative methods,[31,32,35-37,39] and two articles used solely qualitative methods.[33,34] Four studies explored user needs and preferences using focus groups,[32,38] discussion workshops,[33,34] semi-structured interviews,[33,39] and questionnaires.[32] Three articles explored preferences around the design of the web-based resources using discussion workshops,[34,37] and interviews.[31] Seven studies evaluated the usability and/or acceptability of web resources using 'think aloud' cognitive interviews,[36,38-40] focus groups,[35,36] semi-structured interviews,[40] structured interviews,[35] acceptability E-scales,[38] readiness scales,[31] website tracking,[31,37] and online surveys.[31,40] One study evaluated the feasibility of web-resources using a combination of surveys, questionnaires, and structured interviews,[35] and another carried out user testing (separate to usability testing) via interviews and evaluation surveys.[39] See Table 1 for further details of the characteristics of included studies.

For peer review only



For peer review only - http://bmjopen.bmj.com/site/about/guidelines.xhtml

**Table 1. Characteristics of included studies**

| Study (Country) | Population | Methods/Design | Key Outcomes |
|---|---|---|---|
| Amweg et al. (2020)[38]<br><br>(USA) | Hodgkin Lymphoma Survivors n=10<br><br>Healthcare professionals n=9 | Mixed methods, user-centred design<br><br>Two phases:<br>**1)** Focus groups<br>**2)** Usability testing (cognitive interviews and acceptability E-scale) | **User needs and preferences** (Phase 1)<br>Feedback on participants' specification preferences of website<br><br>**Usability** (Phase 2)<br>Feedback of preferences and experience of using website<br>Acceptability of website |
| Ashmore et al. (2020)[34]<br><br>(UK) | Gynaecological cancer patients n=5<br><br>Healthcare professionals n=5<br><br>Researchers n=3 | Qualitative, multi-disciplinary co-creation approach<br><br>Four discussion workshops<br>**1)** Establish understanding of available support and treatment<br>**2)** Establish key areas for support<br>**3)** Website design and requirements<br>**4)** Review of initial resource | **User needs and preferences** (Workshop 1 & 2)<br>Establish understanding of available support and treatment<br>Establish key areas of support<br><br>**Website development** (Workshop 3 & 4)<br>Development of initial resource through creation of a design brief 'wish list'<br>Review of the design of initial resource and identification of recommendations for design team |
| Badr et al. (2016)[39]<br><br>(USA) | Oral cancer survivors **n=13**<br><br>Family caregiver **n=12** | Quantitative and qualitative user-centred design<br><br>Three phases:<br>**1)** Qualitative needs assessment (semi-structured interviews)<br>**2)** Prototype development<br>**3)** Formative evaluation (usability testing - 'think aloud' interviews and user testing – interviews and survey) | **Preferences** (Phase 1)<br>Feedback on the unmet needs and preferences for website<br><br>**Website development** (Phase 2)<br>Development of website prototype<br><br>**Usability/User testing** (Phase 3)<br>Identify navigational difficulties of website<br>Identify participants' experiences of using website<br>Evaluation survey (attractiveness, controllability, efficiency, intuitiveness, learnability) |
| Bartlett et al. (2012)[35]<br><br>(UK) | Cancer patients n=259<br><br>(Breast, Colorectal, Germ cell, Gynaecology, Haematology, Kidney, Prostate, Sarcoma, Upper gastrointestinal) | Quantitative and qualitative user-centred design<br><br>Three phases:<br>**1)** Website design (focus groups and interviews)<br>**2)** Computer and internet survey usage<br>**3)** Crossover study (questionnaires and structured interviews) | **Usability** (Phase 1)<br>Patient feedback on initial web resource<br><br>**Feasibility** (Phase 2)<br>Socio-demographics<br>Computer and internet usage<br><br>**Usability/Feasibility/Acceptability** (Phase 2)<br>Web resource activity tracking<br>Usability and acceptability feedback |
| Bradbury et al. (2019)[36]<br><br>(UK) | Cancer patients n=32<br><br>(Breast, colorectal, and prostate cancer patients)<br><br>Supporters of cancer survivors (nurses, GPs, care assistants, cancer charities) n=31 | Quantitative and qualitative evidence, theory, and user-centred approach<br><br>Seven phases:<br>**1)** Scoping review<br>**2)** Guiding principles<br>**3)** Behavioural analysis<br>**4)** Logic model<br>**5)** Prototype of website<br>**6)** Qualitative optimisation study 1<br>**7)** Qualitative optimisation study 2 | **Usability/Acceptability** (Phase 6 and 7)<br>Feedback of participants experience of exploring website, includes participants' likes, dislikes and recommendations for change |
| Fennell et al. (2017)[31]<br><br>(Australia) | Cancer patients n=122<br><br>(Bones, breast, cervix, colorectal/bowel, lymphoma, lung, melanoma, ovaries, prostate, testicular, brain) | Quantitative and qualitative user-centred approach<br><br>Two phases:<br>1) Website development (interviews)<br>2) Acceptability testing (website tracking activity, readiness scale, online survey) | **Design** (Phase 1)<br>Feedback on initial website content and design<br><br>**Usability/Acceptability** (Phase 2)<br>Website usage<br>Website acceptability and perceived impact |



For peer review only - http://bmjopen.bmj.com/site/about/guidelines.xhtml

**Table 1. Continued**

| Study (Country) | Population | Methods/Design | Key Outcomes |
|---|---|---|---|
| Kapoor et al (2018)[40]<br><br>(USA) | Breast cancer patient or survivor n=15 | Mixed methods, evidence, theory, and user-centred approach<br><br>Seven phases:<br>**1)** Literature review and expert panel<br>**2)** Review of current breast cancer survivorship guidelines and plans<br>**3)** Development of decisions<br>**4)** Curation of decisions<br>**5)** Prototype design and development<br>**6)** User feedback (semi-structured interviews)<br>**7)** Usability testing ('think aloud' and semi-structured interviews and online surveys) | **Perceived usefulness** (Phase 6)<br>Identify participants' perception and perceived usefulness of the website<br><br>**Usability** (Phase 7)<br>Identify the overall usability of the website |
| Pauwels et al (2012)[32]<br><br>(Belgium) | Breast cancer survivors n=57<br><br>Intimate partners n=28 | Quantitative and qualitative user-centred design<br><br>Pre and post design (post-questionnaire, website tracking, care needs questionnaire) | **User needs**<br>Assessment of participants' needs for information and support<br><br>**Design**<br>Evaluation of the content and lay-out of the website. Concepts evaluated include user friendly, well-built, interesting, informative, understandable, new, incomplete, irrelevant, unreliable, too extensive, or confusing<br><br>**Usability**<br>Information gathered about participants' use of the website |
| Santin et al (2019)[37]<br><br>(UK) | Cancer carers n=12 | Quantitative and qualitative co-design approach<br><br>Two phases:<br>**1)** Co-design model<br>-Design of website (workshops and meetings)<br>-Development of prototype 1<br>-User testing phase 1 (unstructured feedback sessions)<br>-Refining prototype<br>**2)** User testing<br>-User testing and refinement (semi-structured interviews, web survey, website tracking)<br>-Final development | **Design** (Phase 1)<br>Evaluation and refinement of website design<br><br>**Usability** (Phase 1 & 2)<br>To gather views and experiences of users' interactions with the web-resource.<br><br>Evaluate website use through tracking website activity |
| Santin et al (2020)[33]<br><br>(Vietnam) | Informal cancer carers n=20<br><br>Healthcare professionals n=23 | Qualitative co-design approach<br><br>Two phases:<br>1) Identification of needs (interviews and focus groups)<br>2) Stakeholder verification and refinements (co-design workshops) | **User needs and perspectives** (Phase 1 & 2)<br>Identifying and understanding the needs of informal cancer carers to underpin resource content<br><br>Learn and agree upon shared priorities and resource components between informal cancer carers and healthcare professionals |



For peer review only - http://bmjopen.bmj.com/site/about/guidelines.xhtml

## Overarching themes

The findings from the ten articles were grouped under the following three areas (1) exploring current evidence, guidelines, and theory (2) identifying user needs and preferences, and (3) evaluating the usability, feasibility, and acceptability of resources.

Exploring current evidence, guidelines, and theory

Bradbury et al.[36] conducted a rapid scoping review to identify the barriers and facilitators to intervention success including the participants needs and attributes and intervention components. Synthesised evidence from the review informed key design objectives including employing an approach that promotes well-being, ensuring the appropriate promotion of behaviour change, providing easy, timely and tailored information, and ensuring an efficient design. These findings were used to establish intervention guiding principles and inform the behavioural analysis and logic model that would underpin resource development. Similarly, Kapoor et al.[40] conducted a literature review to identify the needs of breast cancer survivors to assist in informing web-resource development. The findings, combined with input from an expert panel, helped to identify core functions to be incorporated into the design of a prototype resource including recording and tracking of quality life indicators, recording user-reported treatment-related symptoms, viewing breast cancer related medical history, viewing scheduled follow-up visits, and generating and displaying customised alerts related to symptoms and quality of life issues. Other studies also reported reviewing patient websites and performing literature reviews but were not explicit on how findings informed web-resource development.[31,37]

In addition to reviewing the available literature, studies also reviewed existing guidelines and theory to inform web-resource development. Kapoor et al.[40] conducted a comprehensive review of current breast cancer survivorship guidelines and existing survivorship plans which were used to inform the inclusion of key support information within the web-resource. Badr et al.[39] explored the best practices underpinning the management for oral and swallowing complications following radiotherapy, whilst also reviewing national healthy lifestyle guidelines for cancer survivors and evidence surrounding the self-determination theory. The findings were used to develop a prototype web-resource that specifically focused on promoting survivor and caregiver autonomy, competence, and relatedness; by providing



For peer review only - http://bmjopen.bmj.com/site/about/guidelines.xhtml

tailored information, skill-building education, and support services. Other studies also reported reviewing clinical practice guidelines alongside reviewing the academic literature.[31]

### Identifying the needs and preferences for resources

Participants of the included studies emphasised the need for resources that provide comprehensive information on cancer management and survivorship.[32-34,39] The need for clear information on survivorship care with a specific focus on physical, psychosocial, psychosexual, and emotional well-being was identified;[32,34,38] in addition to information on adjusting to ‘new normal’, returning to work, financial management, and lifestyle advice.[32,34,39] The inclusion of practical advice and information on the side effects of cancer treatments was viewed as essential[34,39] and participants expressed the need to learn from other survivors and carers through shared experiences and self-care strategies.[33,34,39] Concerns were raised by survivors regarding the risk of secondary cancers and how to communicate with family about experiences of cancer survivorship.[34] The inclusion of a ‘Frequently Asked Questions’ page was also proposed to ensure a safe space for users to search for specific information.[33,34]

Reported discussions between healthcare professionals focused on the need to ensure resources can be integrated easily into existing digital systems and are accessible across clinical specialities.[38] It was also considered important that participants did not view resources as a substitute for clinical care[38] and that information on family/carer support be included.[34] Caregivers expressed the need for emotional and supportive information on how to cope with cancer in addition to information on cancer side effects and lifestyle advice.[32,33,39] Concerns were also raised regarding the fear of reoccurrence and the need for specific self-care information and better family communication for carers.[39] Caregivers also discussed the inclusion of information regarding cancer causes and treatment, pain management, hospital administration and treatment processes, hospital daily living, and signposting to skills training.[33,39]



For peer review only - http://bmjopen.bmj.com/site/about/guidelines.xhtml

Evaluating the usability, feasibility, and acceptability of resources

Studies explored the usability, feasibility, and acceptability of resources by qualitatively drawing upon the users' positive and negative experiences of web-resource interaction. Users viewed web-resources positively and valued their use in providing centralised, easily accessible information to support and facilitate survivorship care.[36-38,40] The content included within web-resources were regarded as useful in managing the consequences of cancer and was viewed as a credible source of information due to its development by trusted experts.[36,37,40] Accessing information through web-resources and video formats was perceived as less burdensome than written information and allowed users to easily access advice.[37] Resource features including providing useful website links, being able to access medical history, and tracking quality life indicators was also perceived as valuable components of web-resources.[40]

Whilst web-resources did provide easy access to information, the content of web-resources was considered impersonal with users expressing the need for more customised and prioritised information[35,37,38,40] that was representative of all genders.[37] Web-resources were found to be too complex with users experiencing difficulties in navigating and understanding the purpose of certain web-features highlighting the importance in developing simple and user-friendly web-resources.[35,38,39] Issues with web-resource design were also experienced with users emphasising the need for more appealing web-designs that use appropriate colour and size of both fonts and paragraphs, include greater cross-links, and incorporate much clearer navigational features.[31,38,39]

Studies also evaluated the usability, feasibility and acceptability of web-resources using a range of quantitative methods. A common approach identified was the use of Likert scale style questionnaires and surveys.[32,39,40] For example, Badr et al.[39] reported an overall resource usability score of 80/100 with individual areas rates as attractiveness (4.0/5), controllability (4.2/5), efficiency (4.1/5), intuitiveness (3.9/5), and learnability (3.8/5). Amweg et al.[38] employed an acceptability E-scale to objectively identify web-resource acceptability reporting an overall score of 29.8 (a score of <24 was considered an indicator of web-resource acceptability). Other studies also used descriptive



For peer review only - http://bmjopen.bmj.com/site/about/guidelines.xhtml

questionnaires and surveys with users rating web-resources as easy to use, useful, relevant, necessary, and likely to return and recommend.[35,37] Studies were also shown to objectively explore website usability using website analytics.[31,32,35,37] For example, Santin et al.[37] reported 2769 unique visits between November 2017 and May 2018 of which 743 were returning visitors. Visitors were shown to access multiple website components including the 'getting through treatment', 'caring for you', 'financial', and 'employment' elements. Peer-led videos were the most frequently accessed content whilst professional led material, supporting children, and the emotional aspects of caring were the least visited.

## DISCUSSION

This rapid review has systematically identified and mapped the peer reviewed academic evidence that reported on primary data concerning the development of web-based interventions for supporting PABC. Our findings highlight the use of user-centred, co-designed methodological approaches that are underpinned by iterative, but not necessarily sequential, development processes. A common approach used to develop web-based resources involved the initial exploration of the current evidence, guidelines, and theory followed by an assessment of user needs and preferences to ensure that web-resources were designed to meet the needs of its users. This was typically proceeded by the evaluation of resources involving usability, feasibility, or acceptability testing using a wide range of quantitative, qualitative, and mixed methods that often fed back into further resource refinement. Whilst previous reviews focus predominantly on evaluating the effectiveness of web-based resources, this rapid review differs in that it provides important and novel insights into the methodological approaches that underpin the development and implementation of web-based resources to support PABC. Our findings have the potential to assist other researchers who are developing digital resources and will be used by the current research team to inform the development of a web-based support platform (Shared Lives: Cancer)[25,26] that aims to make qualitative research data on lived cancer experiences publicly available via an open access searchable website.

Ensuring the appropriate design of web-based resources is a critical component of website development[41] in which the use of iterative, co-designed methods is strongly advocated,[42,43]



For peer review only - http://bmjopen.bmj.com/site/about/guidelines.xhtml

especially with respect to cancer care.[44] This is supported by previous evidence that demonstrated the engagement of stakeholders throughout the developmental process ensures that digital tools are firmly grounded within the user's needs, which consequently improves usability and increases user engagement.[45-47] However, there must be an appreciation that users will have varying levels of digital literacy and this needs to be considered when designing and delivering digital resources. Existing research has shown that poor digital literacy is linked with computer anxiety and barriers to internet use amongst PABC.[48,49] Therefore, resources should be accessible and lay friendly to encourage engagement with people who have lower levels of digital literacy. At the same time, there will and continues to be PABC who prefer non-digital support for a variety of reasons. Therefore it is important that face-to-face support is maintained as digital services continue to be widely rolled out as a consequence of both the Covid-19 pandemic and global healthcare policies.

The findings from this review also emphasises the importance of collecting data on usability, feasibility, and acceptability, which are widely considered as important elements when developing web-based resources. An important decision future researchers may face during the ongoing development of digital resources is deciding how these areas will be measure. In line with evidence concerning usability and acceptability testing,[50,51] our findings point towards employing the use of a wide range of quantitative and qualitative methods and where possible should consider a combination of methodologies.[52] Whilst we identify key assessment methods including website analytics, E-scales, questionnaires, 'think aloud' interviews, semi-structured interviews, focus groups and workshops, future research should also consider other methods including more objective and automated methods, especially in the context of usability testing.[50,52]

The development and implementation of digital tools has enormous potential in supporting future healthcare services through transforming the way individuals engage with services and professionals, advancing efficient care coordination, and allowing individuals to better manage one's health and well-being.[53-55] The use of digital technology is now considered a fundamental element that will underpin many of the proposed changes as part of the NHS long-term plan,[11] including desires to facilitate better care and support for individuals at home through the use of digital health tools. As the NHS looks



For peer review only - http://bmjopen.bmj.com/site/about/guidelines.xhtml

to transform and adapt over the next decade, it is important to consider digital health technologies as a potential solution to improve and strengthen aspects or cancer care.[44] The findings of the current review provide important methodological insight that should be used to develop emerging digital health technologies that may help transform and support future healthcare services.

A strength of this review is that it allowed for a rapid synthesis of the current evidence needed to provide timely information to inform the decision-making process surrounding the development and implementation of a novel digital support resource (Shared Lives: Cancer)[25,26] as part of an externally funded project. It provides important insight into the methodological approaches used to develop web-based resources which may be used to guide and inform the design of future digital resources. A limitation of the current review was the lack of consistency and uniformity across outcome measurement tools of included studies, making it challenging to compare and interpret findings. Whilst rapid reviews are key in synthesising timely and informative evidence, it is recognised that the accelerated review process is not subject to the same robustness as a full systematic review. The current rapid review used a streamlined review process that restricted literature searches to one database only and omitted the inclusion of assessing risk of bias. It is therefore acknowledged that the methodology of the current study is less comprehensive and as a consequence the results may be more susceptible to bias and error.

# CONCLUSION

This research adopted a rapid review approach as there is a timely need for an evidence synthesis to support and inform the development of an ongoing project to design an online web-based platform (Shared Lives: Cancer).[25,26] The findings of this rapid review provide an important insight into the methodological approaches used to underpin the development of web-based interventions to support PABC. The evidence generated from this review has the potential to inform and guide future research endeavours when considering the development and implementation of digital resources.



For peer review only - http://bmjopen.bmj.com/site/about/guidelines.xhtml

For peer review only

## AUTHOR CONTRIBUTIONS

SC, DN, HG and RK conceptualised and designed the review. SC reviewed titles, abstracts, full-text articles and extracted data with all data extraction verified by DN. SC and DN prepared the initial manuscript. HG, RK, KM, and MG reviewed and edited the final manuscript.

## ACKNOWLEDGEMENTS

The authors would like to acknowledge the ongoing support of the East Midlands Cancer Alliance and Macmillan Cancer Support with the development of Shared Lives: Cancer.

## FUNDING STATEMENT

This research was supported by the National Institute for Health Research (NIHR) Clinical Research Network (CRN) East Midlands Targeted Funding Call (Grant number/Award number: N/A).

## COMPETING INTERESTS

The authors declare no competing interests.

## PATIENT CONSENT FOR PUBLICATION

Not applicable.

## DATA SHARING

All data are included within the manuscript and supplementary material.

## REFERENCES

1 Arnold M, Rutherford MJ, Bardot A, et al. Progress in cancer survival, mortality, and incidence in seven high-income countries 1995-2014 (ICBP SURVMARK-2): a population-based study. *Lancet Oncol* 2019;20(11):1493-505. doi: 10.1016/s1470-2045(19)30456-5 [published Online First: 2019/09/16]

2 Hanna TP, King WD, Thibodeau S, et al. Mortality due to cancer treatment delay: systematic review and meta-analysis. *BMJ* 2020;371:m4087. doi: 10.1136/bmj.m4087

3 Loud JT, Murphy J. Cancer Screening and Early Detection in the 21(st) Century. *Seminars in oncology nursing* 2017;33(2):121-28. doi: 10.1016/j.soncn.2017.02.002 [published Online First: 2017/03/23]

4 Siegel RL, Miller KD, Fuchs HE, et al. Cancer Statistics, 2021. *CA: A Cancer Journal for Clinicians* 2021;71(1):7-33. doi: https://doi.org/10.3322/caac.21654

5 Bray F, Ferlay J, Soerjomataram I, et al. Global cancer statistics 2018: GLOBOCAN estimates of incidence and mortality worldwide for 36 cancers in 185 countries. *CA Cancer J Clin* 2018;68(6):394-424. doi: 10.3322/caac.21492 [published Online First: 2018/09/13]

6 NCRI. Living with and Beyond Cancer 2020 [Available from: https://www.ncri.org.uk/areas-of-interest/living-with-beyond-cancer/ accessed January 2022.

7 Bosanquet N, Sikora K. The economics of cancer care in the UK. *Lancet Oncol* 2004;5(9):568-74. doi: 10.1016/s1470-2045(04)01569-4 [published Online First: 2004/09/01]



For peer review only - http://bmjopen.bmj.com/site/about/guidelines.xhtml

8 Macklin-Doherty A. Accountability in the NHS: the impact on cancer care. *Ecancermedicalscience* 2018;12:ed83-ed83. doi: 10.3332/ecancer.2018.ed83
9 ICT. Acheiving world-class cancer outcomes: A strategy for England 2015-2020 2015 [updated 2015. Available from: https://www.iccp-portal.org/system/files/plans/Strategy%20-Final.pdf accessed December 2021.
10 Exarchakou A, Rachet B, Belot A, et al. Impact of national cancer policies on cancer survival trends and socioeconomic inequalities in England, 1996-2013: population based study. *Bmj* 2018;360:k764. doi: 10.1136/bmj.k764 [published Online First: 2018/03/16]
11 NHS. The NHS long term plan 2019 [Available from: https://www.longtermplan.nhs.uk/ accessed December 2021.
12 Kemp E, Trigg J, Beatty L, et al. Health literacy, digital health literacy and the implementation of digital health technologies in cancer care: the need for a strategic approach. *Health Promot J Austr* 2021;32 Suppl 1:104-14. doi: 10.1002/hpja.387 [published Online First: 2020/07/19]
13 Dee EC, Muralidhar V, Butler SS, et al. General and Health-Related Internet Use Among Cancer Survivors in the United States: A 2013-2018 Cross-Sectional Analysis. *J Natl Compr Canc Netw* 2020;18(11):1468-75. doi: 10.6004/jnccn.2020.7591 [published Online First: 2020/11/06]
14 Fareed N, Swoboda CM, Jonnalagadda P, et al. Persistent digital divide in health-related internet use among cancer survivors: findings from the Health Information National Trends Survey, 2003-2018. *J Cancer Surviv* 2021;15(1):87-98. doi: 10.1007/s11764-020-00913-8 [published Online First: 2020/07/17]
15 Castleton K, Fong T, Wang-Gillam A, et al. A survey of Internet utilization among patients with cancer. *Support Care Cancer* 2011;19(8):1183-90. doi: 10.1007/s00520-010-0935-5 [published Online First: 2010/06/18]
16 Eysenbach G. The impact of the Internet on cancer outcomes. *CA Cancer J Clin* 2003;53(6):356-71. doi: 10.3322/canjclin.53.6.356 [published Online First: 2004/07/01]
17 Lleras de Frutos M, Casellas-Grau A, Sumalla EC, et al. A systematic and comprehensive review of internet use in cancer patients: Psychological factors. *Psychooncology* 2020;29(1):6-16. doi: 10.1002/pon.5194 [published Online First: 2019/08/07]
18 Mattsson S, Olsson EMG, Johansson B, et al. Health-Related Internet Use in People With Cancer: Results From a Cross-Sectional Study in Two Outpatient Clinics in Sweden. *Journal of medical Internet research* 2017;19(5):e163-e63. doi: 10.2196/jmir.6830
19 Aapro M, Bossi P, Dasari A, et al. Digital health for optimal supportive care in oncology: benefits, limits, and future perspectives. *Support Care Cancer* 2020;28(10):4589-612. doi: 10.1007/s00520-020-05539-1 [published Online First: 2020/06/14]
20 Escriva Boulley G, Leroy T, Bernetière C, et al. Digital health interventions to help living with cancer: A systematic review of participants' engagement and psychosocial effects. *Psychooncology* 2018;27(12):2677-86. doi: 10.1002/pon.4867 [published Online First: 2018/08/29]
21. McCann L, McMillan KA, Pugh G. Digital Interventions to Support Adolescents and Young Adults With Cancer: Systematic Review. *JMIR Cancer* 2019;5(2):e12071. doi: 10.2196/12071 [published Online First: 2019/08/02]
22 Charbonneau DH, Hightower S, Katz A, et al. Smartphone apps for cancer: A content analysis of the digital health marketplace. *Digit Health* 2020;6:2055207620905413. doi: 10.1177/2055207620905413 [published Online First: 2020/02/29]
23 Prochaska JJ, Coughlin SS, Lyons EJ. Social Media and Mobile Technology for Cancer Prevention and Treatment. *Am Soc Clin Oncol Educ Book* 2017;37:128-37. doi: 10.1200/edbk_173841 [published Online First: 2017/06/01]
24 Roberts AL, Fisher A, Smith L, et al. Digital health behaviour change interventions targeting physical activity and diet in cancer survivors: a systematic review and meta-analysis. *J Cancer*



For peer review only - http://bmjopen.bmj.com/site/about/guidelines.xhtml

*Surviv* 2017;11(6):704-19. doi: 10.1007/s11764-017-0632-1 [published Online First: 2017/08/06]
25 Green H ND, Kane R, McPeake K, Willis R. Developing an online resource to support people affected by cancer through making academic research publicly accessible. National Cancer Research Institute (NCRI) virtual showcase Online, 2020.
26 Green H MK, Nelson D, Cooke S, Kane R, . Shared Lives Cancer: An update on the development of an innovative digital health tool to support those affected by cancer. National Cancer Research Institute (NCRI) cancer conference. Online 2021.
27 Moher D, Stewart L, Shekelle P. All in the Family: systematic reviews, rapid reviews, scoping reviews, realist reviews, and more. *Systematic Reviews* 2015;4(1):183. doi: 10.1186/s13643-015-0163-7
28 Khangura S, Konnyu K, Cushman R, et al. Evidence summaries: the evolution of a rapid review approach. *Systematic Reviews* 2012;1(1):10. doi: 10.1186/2046-4053-1-10
29 Tricco AC, Antony J, Zarin W, et al. A scoping review of rapid review methods. *BMC Medicine* 2015;13(1):224. doi: 10.1186/s12916-015-0465-6
30 Moher D, Liberati A, Tetzlaff J, et al. Preferred reporting items for systematic reviews and meta-analyses: the PRISMA statement. *PLoS Med* 2009;6(7):e1000097. doi: 10.1371/journal.pmed.1000097 [published Online First: 2009/07/22]
31 Fennell KM, Turnbull DA, Bidargaddi N, et al. The consumer-driven development and acceptability testing of a website designed to connect rural cancer patients and their families, carers and health professionals with appropriate information and psychosocial support. *Eur J Cancer Care (Engl)* 2017;26(5) doi: 10.1111/ecc.12533 [published Online First: 2016/07/14]
32 Pauwels E, Van Hoof E, Charlier C, et al. Design and process evaluation of an informative website tailored to breast cancer survivors' and intimate partners' post-treatment care needs. *BMC research notes* 2012;5:548-48. doi: 10.1186/1756-0500-5-548
33 Santin O, Jenkins C, Nghiem HLP, et al. The development of a web-based resource to provide information and psychosocial support to informal cancer carers in hospitals in Vietnam. *Psychooncology* 2020;29(5):920-26. doi: 10.1002/pon.5368 [published Online First: 2020/02/27]
34 Ashmore LA, Stewart H, Hutton D, et al. Digital support for living with and beyond gynaecological cancer. *Radiography (Lond)* 2020;26(4):e270-e76. doi: 10.1016/j.radi.2020.03.014 [published Online First: 2020/05/23]
35 Bartlett YK, Selby DL, Newsham A, et al. Developing a useful, user-friendly website for cancer patient follow-up: users' perspectives on ease of access and usefulness. *Eur J Cancer Care (Engl)* 2012;21(6):747-57. doi: 10.1111/j.1365-2354.2012.01357.x [published Online First: 2012/04/27]
36 Bradbury K, Steele M, Corbett T, et al. Developing a digital intervention for cancer survivors: an evidence-, theory- and person-based approach. *NPJ Digit Med* 2019;2:85. doi: 10.1038/s41746-019-0163-4 [published Online First: 2019/09/12]
37 Santin O, McShane T, Hudson P, et al. Using a six-step co-design model to develop and test a peer-led web-based resource (PLWR) to support informal carers of cancer patients. *Psychooncology* 2019;28(3):518-24. doi: 10.1002/pon.4969 [published Online First: 2019/01/01]
38 Amweg LN, McReynolds J, Lansang K, et al. Hodgkin Lymphoma Survivor Wellness: Development of a Web-Based Intervention. *Clin J Oncol Nurs* 2020;24(3):284-89. doi: 10.1188/20.Cjon.284-289 [published Online First: 2020/05/23]
39 Badr H, Lipnick D, Diefenbach MA, et al. Development and usability testing of a web-based self-management intervention for oral cancer survivors and their family caregivers. *Eur J Cancer Care (Engl)* 2016;25(5):806-21. doi: 10.1111/ecc.12396 [published Online First: 2015/10/29]



For peer review only - http://bmjopen.bmj.com/site/about/guidelines.xhtml

40 Kapoor A, Nambisan P. Usability and acceptance evaluation of ACESO: a Web-based breast cancer survivorship tool. *J Cancer Surviv* 2018;12(3):316-25. doi: 10.1007/s11764-017-0670-8 [published Online First: 2018/01/27]
41 Garett R, Chiu J, Zhang L, et al. A Literature Review: Website Design and User Engagement. *Online J Commun Media Technol* 2016;6(3):1-14.
42 Leask CF, Sandlund M, Skelton DA, et al. Framework, principles and recommendations for utilising participatory methodologies in the co-creation and evaluation of public health interventions. *Res Involv Engagem* 2019;5:2. doi: 10.1186/s40900-018-0136-9 [published Online First: 2019/01/18]
43 Skivington K, Matthews L, Simpson SA, et al. A new framework for developing and evaluating complex interventions: update of Medical Research Council guidance. *BMJ* 2021;374:n2061. doi: 10.1136/bmj.n2061
44 Hesse BW, Kwasnicka D, Ahern DK. Emerging digital technologies in cancer treatment, prevention, and control. *Transl Behav Med* 2021;11(11):2009-17. doi: 10.1093/tbm/ibab033 [published Online First: 2021/12/02]
45 Bjerkan J, Hedlund M, Hellesø R. Patients' contribution to the development of a web-based plan for integrated care - a participatory design study. *Inform Health Soc Care* 2015;40(2):167-84. doi: 10.3109/17538157.2014.907803 [published Online First: 2014/05/03]
46 Davenport T, Milton A, Ospina-Pinillos L, et al. Project Synergy research and development cycle: iterative processes of participatory design, user testing, implementation and feasibility testing. *Med J Aust* 2019;211(7):S8-11.
47 LaMonica HM, Davenport TA, Burns J, et al. Technology-Enabled Mental Health Service Reform for Open Arms - Veterans and Families Counselling: Participatory Design Study. *JMIR Form Res* 2019;3(3):e13662. doi: 10.2196/13662 [published Online First: 2019/09/21]
48 Lepore SJ, Rincon MA, Buzaglo JS, et al. Digital literacy linked to engagement and psychological benefits among breast cancer survivors in Internet-based peer support groups. *European journal of cancer care* 2019;28(4):e13134-e34. doi: 10.1111/ecc.13134
49 Hoogland AI, Mansfield J, Lafranchise EA, et al. eHealth literacy in older adults with cancer. *J Geriatr Oncol* 2020;11(6):1020-22. doi: 10.1016/j.jgo.2019.12.015 [published Online First: 2020/01/10]
50 Maramba I, Chatterjee A, Newman C. Methods of usability testing in the development of eHealth applications: A scoping review. *Int J Med Inform* 2019;126:95-104. doi: 10.1016/j.ijmedinf.2019.03.018 [published Online First: 2019/04/29]
51 Sekhon M, Cartwright M, Francis JJ. Acceptability of healthcare interventions: an overview of reviews and development of a theoretical framework. *BMC Health Services Research* 2017;17(1):88. doi: 10.1186/s12913-017-2031-8
52 Zapata BC, Fernández-Alemán JL, Idri A, et al. Empirical Studies on Usability of mHealth Apps: A Systematic Literature Review. *Journal of Medical Systems* 2015;39(2):1. doi: 10.1007/s10916-014-0182-2
53 Manteghinejad A, Javanmard SH. Challenges and opportunities of digital health in a post-COVID19 world. *J Res Med Sci* 2021;26:11-11. doi: 10.4103/jrms.JRMS_1255_20
54 Meskó B, Drobni Z, Bényei É, et al. Digital health is a cultural transformation of traditional healthcare. *mHealth* 2017;3:38-38. doi: 10.21037/mhealth.2017.08.07
55 Powell J, Newhouse N, Boylan A-M, et al. Digital health citizens and the future of the NHS. *Digital health* 2016;2:2055207616672033-33. doi: 10.1177/2055207616672033

## References included within tables

31 Fennell KM, Turnbull DA, Bidargaddi N, et al. The consumer-driven development and acceptability testing of a website designed to connect rural cancer patients and their families, carers and



For peer review only - http://bmjopen.bmj.com/site/about/guidelines.xhtml

health professionals with appropriate information and psychosocial support. *Eur J Cancer Care (Engl)* 2017;26(5) doi: 10.1111/ecc.12533 [published Online First: 2016/07/14]

32 Pauwels E, Van Hoof E, Charlier C, et al. Design and process evaluation of an informative website tailored to breast cancer survivors' and intimate partners' post-treatment care needs. *BMC research notes* 2012;5:548-48. doi: 10.1186/1756-0500-5-548

33 Santin O, Jenkins C, Nghiem HLP, et al. The development of a web-based resource to provide information and psychosocial support to informal cancer carers in hospitals in Vietnam. *Psychooncology* 2020;29(5):920-26. doi: 10.1002/pon.5368 [published Online First: 2020/02/27]

34 Ashmore LA, Stewart H, Hutton D, et al. Digital support for living with and beyond gynaecological cancer. *Radiography (Lond)* 2020;26(4):e270-e76. doi: 10.1016/j.radi.2020.03.014 [published Online First: 2020/05/23]

35 Bartlett YK, Selby DL, Newsham A, et al. Developing a useful, user-friendly website for cancer patient follow-up: users' perspectives on ease of access and usefulness. *Eur J Cancer Care (Engl)* 2012;21(6):747-57. doi: 10.1111/j.1365-2354.2012.01357.x [published Online First: 2012/04/27]

36 Bradbury K, Steele M, Corbett T, et al. Developing a digital intervention for cancer survivors: an evidence-, theory- and person-based approach. *NPJ Digit Med* 2019;2:85. doi: 10.1038/s41746-019-0163-4 [published Online First: 2019/09/12]

37 Santin O, McShane T, Hudson P, et al. Using a six-step co-design model to develop and test a peer-led web-based resource (PLWR) to support informal carers of cancer patients. *Psychooncology* 2019;28(3):518-24. doi: 10.1002/pon.4969 [published Online First: 2019/01/01]

38 Amweg LN, McReynolds J, Lansang K, et al. Hodgkin Lymphoma Survivor Wellness: Development of a Web-Based Intervention. *Clin J Oncol Nurs* 2020;24(3):284-89. doi: 10.1188/20.Cjon.284-289 [published Online First: 2020/05/23]

39 Badr H, Lipnick D, Diefenbach MA, et al. Development and usability testing of a web-based self-management intervention for oral cancer survivors and their family caregivers. *Eur J Cancer Care (Engl)* 2016;25(5):806-21. doi: 10.1111/ecc.12396 [published Online First: 2015/10/29]

40 Kapoor A, Nambisan P. Usability and acceptance evaluation of ACESO: a Web-based breast cancer survivorship tool. *J Cancer Surviv* 2018;12(3):316-25. doi: 10.1007/s11764-017-0670-8 [published Online First: 2018/01/27]

For peer review only

## References included within figures

30 Moher D, Liberati A, Tetzlaff J, et al. Preferred reporting items for systematic reviews and meta-analyses: the PRISMA statement. *PLoS Med* 2009;6(7):e1000097. doi: 10.1371/journal.pmed.1000097 [published Online First: 2009/07/22]

## Figure legends

**Figure 1.** Study flowchart[30]



For peer review only - http://bmjopen.bmj.com/site/about/guidelines.xhtml

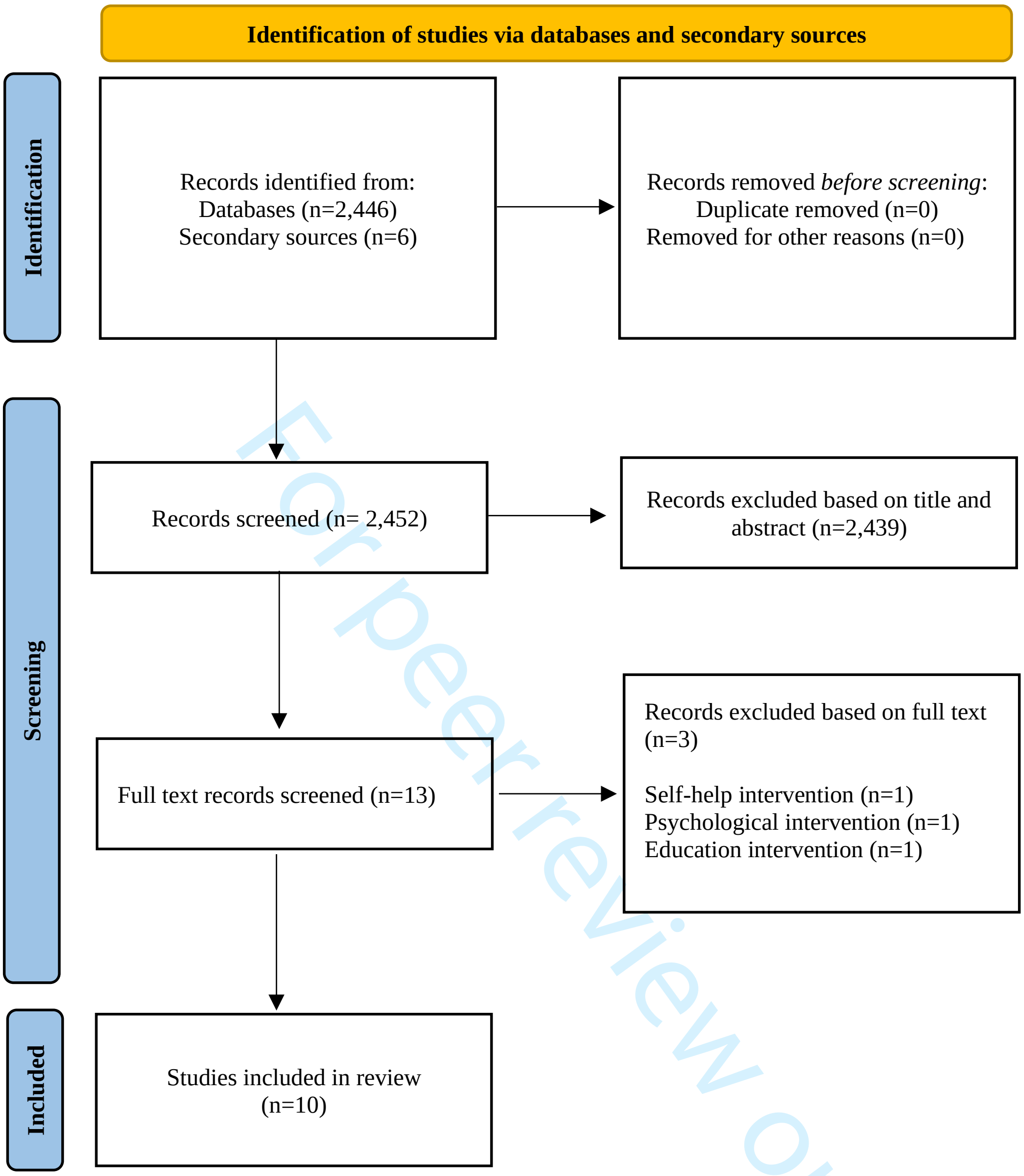


For peer review only

# PRISMA 2020 Checklist

| Section and Topic | Item # | Checklist item | Location where item is reported |
|---|---|---|---|
| **TITLE** | | | |
| Title | 1 | Identify the report as a systematic review. | Page 1 |
| **ABSTRACT** | | | |
| Abstract | 2 | See the PRISMA 2020 for Abstracts checklist. | Page 2 |
| **INTRODUCTION** | | | |
| Rationale | 3 | Describe the rationale for the review in the context of existing knowledge. | Page 4/5 |
| Objectives | 4 | Provide an explicit statement of the objective(s) or question(s) the review addresses. | Page 2/5 |
| **METHODS** | | | |
| Eligibility criteria | 5 | Specify the inclusion and exclusion criteria for the review and how studies were grouped for the syntheses. | Page 7 |
| Information sources | 6 | Specify all databases, registers, websites, organisations, reference lists and other sources searched or consulted to identify studies. Specify the date when each source was last searched or consulted. | Page 6 |
| Search strategy | 7 | Present the full search strategies for all databases, registers and websites, including any filters and limits used. | Supplementary material (S2) |
| Selection process | 8 | Specify the methods used to decide whether a study met the inclusion criteria of the review, including how many reviewers screened each record and each report retrieved, whether they worked independently, and if applicable, details of automation tools used in the process. | Page 6/7 |
| Data collection process | 9 | Specify the methods used to collect data from reports, including how many reviewers collected data from each report, whether they worked independently, any processes for obtaining or confirming data from study investigators, and if applicable, details of automation tools used in the process. | Page 7 |
| Data items | 10a | List and define all outcomes for which data were sought. Specify whether all results that were compatible with each outcome domain in each study were sought (e.g. for all measures, time points, analyses), and if not, the methods used to decide which results to collect. | Page 7 |
| | 10b | List and define all other variables for which data were sought (e.g. participant and intervention characteristics, funding sources). Describe any assumptions made about any missing or unclear information. | Page 7 |
| Study risk of bias assessment | 11 | Specify the methods used to assess risk of bias in the included studies, including details of the tool(s) used, how many reviewers assessed each study and whether they worked independently, and if applicable, details of automation tools used in the process. | N/A |
| Effect measures | 12 | Specify for each outcome the effect measure(s) (e.g. risk ratio, mean difference) used in the synthesis or presentation of results. | N/A |
| Synthesis methods | 13a | Describe the processes used to decide which studies were eligible for each synthesis (e.g. tabulating the study intervention characteristics and comparing against the planned groups for each synthesis (item #5)). | Page 7 |
| | 13b | Describe any methods required to prepare the data for presentation or synthesis, such as handling of missing summary statistics, or data conversions. | N/A |
| | 13c | Describe any methods used to tabulate or visually display results of individual studies and syntheses. | Page 7 |
| | 13d | Describe any methods used to synthesize results and provide a rationale for the choice(s). If meta-analysis was performed, describe the model(s), method(s) to identify the presence and extent of statistical heterogeneity, and software package(s) used. | Page 7 |
| | 13e | Describe any methods used to explore possible causes of heterogeneity among study results (e.g. subgroup analysis, meta-regression). | N/A |
| | 13f | Describe any sensitivity analyses conducted to assess robustness of the synthesized results. | N/A |
| Reporting bias assessment | 14 | Describe any methods used to assess risk of bias due to missing results in a synthesis (arising from reporting biases). | N/A |
| Certainty | 15 | Describe any methods used to assess certainty (or confidence) in the body of evidence for an outcome. | N/A |

For peer review only - http://bmjopen.bmj.com/site/about/guidelines.xhtml

# PRISMA 2020 Checklist

| Section and Topic | Item # | Checklist item | Location where item is reported |
|---|---|---|---|
| assessment | | | |
| **RESULTS** | | | |
| Study selection | 16a | Describe the results of the search and selection process, from the number of records identified in the search to the number of studies included in the review, ideally using a flow diagram. | Page 8 and Figure 1 |
| | 16b | Cite studies that might appear to meet the inclusion criteria, but which were excluded, and explain why they were excluded. | Figure 1 |
| Study characteristics | 17 | Cite each included study and present its characteristics. | Page 8/9/10/11 (Table 1) |
| Risk of bias in studies | 18 | Present assessments of risk of bias for each included study. | N/A |
| Results of individual studies | 19 | For all outcomes, present, for each study: (a) summary statistics for each group (where appropriate) and (b) an effect estimate and its precision (e.g. confidence/credible interval), ideally using structured tables or plots. | N/A |
| Results of syntheses | 20a | For each synthesis, briefly summarise the characteristics and risk of bias among contributing studies. | N/A |
| | 20b | Present results of all statistical syntheses conducted. If meta-analysis was done, present for each the summary estimate and its precision (e.g. confidence/credible interval) and measures of statistical heterogeneity. If comparing groups, describe the direction of the effect. | N/A |
| | 20c | Present results of all investigations of possible causes of heterogeneity among study results. | N/A |
| | 20d | Present results of all sensitivity analyses conducted to assess the robustness of the synthesized results. | N/A |
| Reporting biases | 21 | Present assessments of risk of bias due to missing results (arising from reporting biases) for each synthesis assessed. | N/A |
| Certainty of evidence | 22 | Present assessments of certainty (or confidence) in the body of evidence for each outcome assessed. | N/A |
| **DISCUSSION** | | | |
| Discussion | 23a | Provide a general interpretation of the results in the context of other evidence. | Page 15/16/17 |
| | 23b | Discuss any limitations of the evidence included in the review. | Page 17 |
| | 23c | Discuss any limitations of the review processes used. | Page 16/17 |
| | 23d | Discuss implications of the results for practice, policy, and future research. | Page 15/16/17 |
| **OTHER INFORMATION** | | | |
| Registration and protocol | 24a | Provide registration information for the review, including register name and registration number, or state that the review was not registered. | Page 5 |
| | 24b | Indicate where the review protocol can be accessed, or state that a protocol was not prepared. | Page 5 |
| | 24c | Describe and explain any amendments to information provided at registration or in the protocol. | N/A |
| Support | 25 | Describe sources of financial or non-financial support for the review, and the role of the funders or sponsors in the review. | Page 18 |
| Competing interests | 26 | Declare any competing interests of review authors. | Page 18 |
| Availability of data, code and other materials | 27 | Report which of the following are publicly available and where they can be found: template data collection forms; data extracted from included studies; data used for all analyses; analytic code; any other materials used in the review. | Page 7/18 |

For peer review only

# PRISMA 2020 Checklist

*From:* Page MJ, McKenzie JE, Bossuyt PM, Boutron I, Hoffmann TC, Mulrow CD, et al. The PRISMA 2020 statement: an updated guideline for reporting systematic reviews. BMJ 2021;372:n71. doi: 10.1136/bmj.n71

For more information, visit: http://www.prisma-statement.org/

For peer review only

# Developing web-based interventions to support people living with and affected by cancer: a protocol for a rapid review

Dr David Nelson[1], Dr Samuel Cooke[2], Heidi Green[2], Kathie McPeake[3], Prof. Mark Gussy[1], Dr Ros Kane[2]

[1] Lincoln International Institute for Rural Health, University of Lincoln, UK.

[2] School of Health and Social Care, University of Lincoln, UK.

[3] NHS Lincolnshire Clinical Commissioning Group

Correspondence to Dr David Nelson; dnelson@lincoln.ac.uk

## Abstract

**Introduction:** More people are living with and beyond cancer and digital interventions are increasingly being used to support them at all stages through their journey. This rapid review aims to systematically identify and explore the existing evidence that reports on primary data concerning the development of web-based interventions used to support people living with and affected by cancer.

**Methods and analysis:** Keyword searches were performed in MEDLINE to identify peer-reviewed literature on web-based interventions that are designed to support people living with and affected by cancer. The review will include studies published in the English language and will not have any restrictions on publication date or geography. Screening and data extraction will be completed independently by two reviewers. The included studies will be tabulated and the results synthesised narratively.

**Discussion:** This rapid review aims to identify and synthesise the peer-reviewed academic literature that reports on primary data concerning the development of web-based interventions to support people living with and affected by cancer. This methodology was chosen to rapidly synthesise the existing peer-reviewed evidence to support the development and design of an online web-based platform that the team are working on to make qualitative research data on lived cancer experience publicly available and accessible.

**Ethics and dissemination:** The review was registered and given a favourable ethical opinion on the 19/07/21 by a committee at the University of Lincoln (Review ref: 2021_6976). The findings from this rapid review will be presented at appropriate conferences and published in a peer reviewed academic journal as well as a report for the National Institute for Health Research Clinical Research Network.

**Systematic review registration:** The protocol was registered on the Open Science Framework [insert link here].

**Keywords:** cancer survivorship; web-based support; user experience; rapid review; protocol



For peer review only - http://bmjopen.bmj.com/site/about/guidelines.xhtml

# Introduction

Globally, cancer incidence is increasing with an estimated 18.1 million new diagnoses in 2018.[1] This can be attributed to advances in screening, earlier detection, diagnostic methods and improved treatments. Consequently, more and more people are now surviving cancer, and in the UK it is predicted that there will be four million people living with and beyond cancer by 2030.[2] Cancer raises a wide range of specific issues pertaining to information provision and emotional support [3] and there are now an increasing number of online health communities for people affected by cancer, each with their own specific aims.[4-6] Existing research has shown that people living with and affected by cancer use the internet for (1) content (online health information) (2) communication (e-mail and instant messaging) (3) communities (virtual support groups and forums) and (4) e-commerce (selling or buying products). [7]

The ongoing Covid-19 pandemic poses several challenges to oncology services and people living with and affected by cancer may now rely more heavily on digital and remote support.[8 9] Consequently, some psychosocial and supportive cancer care has now shifted from face-to-face to virtual delivery.[10] Digital health technologies have the potential to reduce health inequalities in cancer care and can improve access, integration and personalisation of care.[6] They can be particularly beneficial to those in rural and remote settings where access has long been acknowledged as a barrier to care.[11] However, the benefits of digital health technology depend partly on digital health literacy (capabilities and resources required by people to use and benefit from it).[6]

Existing reviews have previously explored the use of web-based interventions for supporting people living with and beyond cancer. These include identifying and evaluating the effectiveness of a wide range of web-based resources such as educational and psychosocial platforms[5 12 13], social media sites[14], mobile applications[14 15], and digital health interventions that focus on specific health behaviours e.g. physical activity and diet.[16] The current review will differ to previous reviews by exploring the academic evidence that reports on user and developer experience/perception for building and developing web-based tools. The evidence synthesised from the review will be used to directly inform the development of a novel web-based resource that will support those living with and beyond cancer through making qualitative research data on lived cancer experience publicly available and accessible.

This rapid review will aim to achieve the following:

- ❖ Identify and map the peer reviewed academic evidence that reports on primary data concerning the development and utilisation of web-based tools for supporting people living with and affected by cancer.

- ❖ Collate and analyse primary data with a view to informing evidence-based recommendations for the development of a novel and accessible web-based tool that meets the needs of people living with and affected by cancer.



For peer review only - http://bmjopen.bmj.com/site/about/guidelines.xhtml

## Methods

The team will utilise a rapid review approach which is now considered a key component of the knowledge synthesis family alongside systematic reviews, scoping reviews and realist reviews.[17] Despite the increase in popularity of rapid review methods there is still no universal agreement within the extant literature as to how a rapid review should be conducted or defined.[18-20] In sum, rapid reviews are a form of evidence synthesis in which components of the systematic review process are omitted or simplified with a view to producing findings in a timely manner. [18 19] Still, rapid reviews must remain systematic in their approach and have a duty to report the methods in a transparent manner making sure that they are clear about deviations or omissions from the Preferred Reporting Items for Systematic Reviews and Meta-Analyses (PRISMA) criteria.[21] This review will be conducted in line with the recently published guidance from the Cochrane Rapid Reviews Methods Group[22] and reported using the PRISMA checklist.[23]

*Protocol and Ethics*

The protocol has been registered on the Open Science Framework [insert link here]. This rapid review will be conducted over a 3-4 month period (July 2021 – October 2021).

The review was given a favourable ethical opinion by a committee at the University of Lincoln (Review ref: 2021_6976) on 19/07/21.

*Search Strategy*

We used keyword searches together with Boolean operators (OR and AND) and truncation (*) to locate relevant peer-reviewed literature on the user experience of web-based support that is delivered to people living with and affected by cancer. We searched MEDLINE (20/07/21) which is the leading full-text database of biomedical and health journals. The primary search strategy and syntax was developed and refined by three members of the review team (DN, SC, HG). The search strategy for MEDLINE can be found at Appendix 1. Due to time constraints, limited sources and the need to produce findings in a timely manner, we are limiting our search to one database which is considered acceptable for a rapid systematic review.[18 19] All database searches were supplemented with google scholar searches in addition with forward and backward citation searchers of relevant articles. Database searches will continually be updated to identify and incorporate the most up to date evidence where appropriate.

To identify people living with and affected by cancer we will use the following keywords: "cancer surviv*" or "living with cancer" or "living with and beyond cancer" or "cancer patient*" or "patients with cancer" "people affected by cancer" or "oncology patient" or "cancer experience*" or "cancer management" or "cancer support" or "cancer care*"

The following keywords will be used to identify web-based support and interventions: "web*" or "internet*" or "online*" or "digital*"

To search literature on user experience we will use the following keywords: "user experience*" or "usability" or "functionality" or "design" or "interaction" or "development" or "user testing"



For peer review only - http://bmjopen.bmj.com/site/about/guidelines.xhtml

*Inclusion and Exclusion Criteria*

Peer-reviewed publications will be selected for inclusion in the review utilising the pre-defined eligibility criteria outlined in Table 1 below.

**Table 1 Inclusion and Exclusion Criteria**

| Criterion | Include | Exclude |
|---|---|---|
| Population | Adults (18+)<br>All genders<br>People living with cancer/caregivers/people affected by cancer<br>Any geography. | Non-adult populations (under 18). |
| Intervention | Website based/internet-based cancer support resources. | Support programmes that focus solely on mobile and digital apps.<br><br>E-learning programmes or interventions (self-directed and practitioner/professionally lead).<br><br>Social media/networking sites |
| Comparator | Not applicable. | Not applicable. |
| Outcomes | Reports primary data on user and developer experience/usability/functionality/design on web/internet-based support for people living with and affected by cancer. | There are no primary data reported on user experience/usability/functionality/design on web/internet-based support for people living with and affected by cancer. |
| Study design | Reports empirical research data using the following designs:<br>• Quantitative<br>• Qualitative<br>• Mixed Methods | • Systematic and literature reviews<br>• Editorials<br>• Commentaries<br>• Opinion pieces<br>• Case series or reports |
| Language | Published in the English language. | Not published in the English language. |

## Data and Analysis

*Record Selection*

References identified via the search were exported and managed using Endnote Version X9. The final search identified 2,452 articles for screening, see Appendix 1. The titles and abstracts will be independently screened against the eligibility criteria by two reviewers (DN and SC). Where discrepancies exist, the team will aim to resolve via discussion or through a third reviewer (HG). Following title and abstract screening, the remaining articles will be independently screened by full text, for inclusion by two reviewers (DN and SC), with any disagreements resolved through discussion or a third reviewer (RK). The results will be presented in a PRISMA flow diagram.

*Data Extraction*

Data will be extracted using an adapted Cochrane Data Extraction Template and this can be found at Appendix 2. This will be piloted with a subset (*n*=5) of full text studies that meet our eligibility criteria to determine whether any further changes are needed to the data extraction



For peer review only - http://bmjopen.bmj.com/site/about/guidelines.xhtml

template. After piloting the tool, two reviewers (DN and SC) will undertake data extraction for each full text article with cross checking for data quality taking place by a third reviewer (HG). Study characteristics will be extracted from each study based upon (1) study methods (e.g. aims/objectives, study design, participants, outcomes) (2) details on the web-based intervention/support and (3) study findings (details of all relevant data concerning user experience, usability, functionality, and design). All discrepancies will be resolved through further discussion, or where required, a fourth reviewer (RK).

*Data synthesis and analysis*

It is likely that the review will include a wide range of study designs that make use of both quantitative and qualitative methodologies. Following data extraction, the results of the full text articles will be tabulated. Quantitative data will be described using basic descriptive statistics as well as being written up narratively. We do not plan to conduct a formal statistical meta-analysis. Qualitative findings on user experience will be analysed using thematic synthesis.[24] Where possible we will group and comment on similarities and dissimilarities within the user experience data.

*Quality assessment*

The focus of this rapid review is on identifying and exploring the literature on user experience of web-based support that is delivered to people living with and beyond cancer, therefore, a quality assessment of included articles was not deemed appropriate.

## Discussion

The aim of this research is to identify and map the peer reviewed academic evidence that reports on primary data concerning the development of web-based tools that support people living with and affected by cancer. It will also aim to collate and analyse data with a view to informing evidence-based recommendations for the development of a novel and accessible web-based tool that meets the needs of people living with and affected by cancer. It has been acknowledged that digital interventions have the potential to provide an excellent source of support for people living with and affected by cancer.[5 25] Specifically, they can help people to cope better with the disease and with side effects as well as improving self-management and wellbeing.[5]

A rapid review methodology was chosen to support and inform the timely need for the development and implementation of an innovative online web-based platform that is informed by peer-reviewed academic evidence. Rapid reviews are useful in adapting to and overcoming time and resource constraints that genuinely prevent the development and execution of a high-quality systematic review.[22] Nevertheless, researchers must endeavour to optimise the methodological rigour, clarity, and reproducibility of a rapid review, starting with the implementation of a rigorous and systematic protocol.[22]

Systematic reviews and meta-analyses are often intensive[26], especially when considering the large number of included studies, which is likely to be the case for evidence concerning user experience for developing web-based interventions in cancer. Whilst conducting a rapid review may accelerate the development process, it will enable a timely evidence appraisal that will address priority research questions as well as allowing for the rapid dissemination of findings.



For peer review only - http://bmjopen.bmj.com/site/about/guidelines.xhtml

*Dissemination*

This rapid review and wider work (development of a web-based platform to support people living with and affected by cancer) benefits from the establishment of a study steering group with representation from academic researchers, cancer professionals and people with lived cancer experience. The review team will draw on their expertise and the findings will be disseminated in accordance with an ongoing dissemination strategy that will be developed collaboratively by the review team and steering group. This will involve presenting at appropriate local and national conferences, as well as, publishing in a peer reviewed academic journal. A summary of the findings will also be written up as a report for the National Institute for Health Research (NIHR) Clinical Research Network (CRN) who are funding this work. The team will disseminate the results in lay and accessible formats including using social media and press releases via the University of Lincoln and Macmillan Cancer Support.

## Funding

This review was funded by the National Institute for Health Research (NIHR) Clinical Research Network (CRN).

## Acknowledgements

We would like to acknowledge the Steering Group for their support in directing this review as well as the wider project. Acknowledgments must also go to Macmillan Cancer Support and the East Midlands Cancer Alliance for previous funding and their continued support throughout the early development of the Shared Lives Cancer project.

## References

1. Bray F, Ferlay J, Soerjomataram I, et al. Global cancer statistics 2018: GLOBOCAN estimates of incidence and mortality worldwide for 36 cancers in 185 countries. *CA: A Cancer Journal for Clinicians* 2018;68(6):394-424. doi: https://doi.org/10.3322/caac.21492
2. Maddams J, Utley M, Møller H. Projections of cancer prevalence in the United Kingdom, 2010–2040. *British Journal of Cancer* 2012;107(7):1195-202. doi: 10.1038/bjc.2012.366
3. van Eenbergen MC, van de Poll-Franse LV, Heine P, et al. The Impact of Participation in Online Cancer Communities on Patient Reported Outcomes: Systematic Review. *JMIR Cancer* 2017;3(2):e15. doi: 10.2196/cancer.7312
4. van Eenbergen MC, van de Poll-Franse LV, Krahmer E, et al. Analysis of Content Shared in Online Cancer Communities: Systematic Review. *JMIR Cancer* 2018;4(1):e6. doi: 10.2196/cancer.7926
5. Escriva Boulley G, Leroy T, Bernetière C, et al. Digital health interventions to help living with cancer: A systematic review of participants' engagement and psychosocial effects. *Psycho-Oncology* 2018;27(12):2677-86. doi: https://doi.org/10.1002/pon.4867
6. Kemp E, Trigg J, Beatty L, et al. Health literacy, digital health literacy and the implementation of digital health technologies in cancer care: the need for a strategic approach. *Health Promot J Austr* 2021;32 Suppl 1:104-14. doi: 10.1002/hpja.387
7. Eysenbach G. The Impact of the Internet on Cancer Outcomes. *CA: A Cancer Journal for Clinicians* 2003;53(6):356-71. doi: https://doi.org/10.3322/canjclin.53.6.356
8. Al-Quteimat OM, Amer AM. The Impact of the COVID-19 Pandemic on Cancer Patients. *Am J Clin Oncol* 2020;43(6):452-55. doi: 10.1097/COC.0000000000000712
9. Burki TK. Cancer guidelines during the COVID-19 pandemic. *The Lancet Oncology* 2020;21(5):629-30. doi: 10.1016/S1470-2045(20)30217-5
10. Bultz BD, Watson L. Lessons learned about virtual cancer care and distress screening in the time of COVID-19. *Supportive Care in Cancer* 2021 doi: 10.1007/s00520-021-06322-6



For peer review only - http://bmjopen.bmj.com/site/about/guidelines.xhtml

11. Douthit N, Kiv S, Dwolatzky T, et al. Exposing some important barriers to health care access in the rural USA. *Public Health* 2015;129(6):611-20. doi: https://doi.org/10.1016/j.puhe.2015.04.001
12. Aapro M, Bossi P, Dasari A, et al. Digital health for optimal supportive care in oncology: benefits, limits, and future perspectives. *Support Care Cancer* 2020;28(10):4589-612. doi: 10.1007/s00520-020-05539-1 [published Online First: 2020/06/14]
13. McCann L, McMillan KA, Pugh G. Digital Interventions to Support Adolescents and Young Adults With Cancer: Systematic Review. *JMIR Cancer* 2019;5(2):e12071. doi: 10.2196/12071 [published Online First: 2019/08/02]
14. Charbonneau DH, Hightower S, Katz A, et al. Smartphone apps for cancer: A content analysis of the digital health marketplace. *DIGITAL HEALTH* 2020;6:2055207620905413. doi: 10.1177/2055207620905413
15. Prochaska JJ, Coughlin SS, Lyons EJ. Social Media and Mobile Technology for Cancer Prevention and Treatment. *Am Soc Clin Oncol Educ Book* 2017;37:128-37. doi: 10.1200/edbk_173841 [published Online First: 2017/06/01]
16. Roberts AL, Fisher A, Smith L, et al. Digital health behaviour change interventions targeting physical activity and diet in cancer survivors: a systematic review and meta-analysis. *J Cancer Surviv* 2017;11(6):704-19. doi: 10.1007/s11764-017-0632-1 [published Online First: 2017/08/06]
17. Moher D, Stewart L, Shekelle P. All in the Family: systematic reviews, rapid reviews, scoping reviews, realist reviews, and more. *Systematic Reviews* 2015;4(1):183. doi: 10.1186/s13643-015-0163-7
18. Khangura S, Konnyu K, Cushman R, et al. Evidence summaries: the evolution of a rapid review approach. *Systematic Reviews* 2012;1(1):10. doi: 10.1186/2046-4053-1-10
19. Tricco AC, Antony J, Zarin W, et al. A scoping review of rapid review methods. *BMC Medicine* 2015;13(1):224. doi: 10.1186/s12916-015-0465-6
20. Kelly SE, Moher D, Clifford TJ. DEFINING RAPID REVIEWS: A MODIFIED DELPHI CONSENSUS APPROACH. *International Journal of Technology Assessment in Health Care* 2016;32(4):265-75. doi: 10.1017/S0266462316000489 [published Online First: 2016/10/11]
21. Schünemann HJ, Moja L. Reviews: Rapid! Rapid! Rapid! …and systematic. *Systematic Reviews* 2015;4(1):4. doi: 10.1186/2046-4053-4-4
22. Garritty C, Gartlehner G, Nussbaumer-Streit B, et al. Cochrane Rapid Reviews Methods Group offers evidence-informed guidance to conduct rapid reviews. *Journal of Clinical Epidemiology* 2021;130:13-22. doi: https://doi.org/10.1016/j.jclinepi.2020.10.007
23. Moher D, Liberati A, Tetzlaff J, et al. Preferred Reporting Items for Systematic Reviews and Meta-Analyses: The PRISMA Statement. *PLOS Medicine* 2009;6(7):e1000097. doi: 10.1371/journal.pmed.1000097
24. Thomas J, Harden A. Methods for the thematic synthesis of qualitative research in systematic reviews. *BMC Medical Research Methodology* 2008;8(1):45. doi: 10.1186/1471-2288-8-45
25. Laing CM, Moules NJ, Sinclair S, et al. Digital Storytelling as a Psychosocial Tool for Adult Cancer Survivors. *Oncol Nurs Forum* 2019;46(2):147-54. doi: 10.1188/19.onf.147-154
26. Allen IE, Olkin I. Estimating time to conduct a meta-analysis from number of citations retrieved. *Jama* 1999;282(7):634-5. doi: 10.1001/jama.282.7.634 [published Online First: 1999/10/12]



For peer review only - http://bmjopen.bmj.com/site/about/guidelines.xhtml

## Appendix 1 Search Strategy for MEDLINE

| Key search terms | Date | Hits (*n*=) | Parameters |
|---|---|---|---|
| S1: “Cancer surviv*” | 20/07/21 | 27,751 | Medline only |
| S2: “Living with cancer” | 20/07/21 | 865 | Medline only |
| S3: “Living with and beyond cancer” | 20/07/21 | 103 | Medline only |
| S4: “Cancer patient*” | 20/07/21 | 203,503 | Medline only |
| S5: “Patients with cancer” | 20/07/21 | 31,724 | Medline only |
| S6: “People affected by cancer” | 20/07/21 | 146 | Medline only |
| S7: “Oncology patient*” | 20/07/21 | 6,036 | Medline only |
| S8: “Cancer experience*” | 20/07/21 | 2,911 | Medline only |
| S9: “Cancer management” | 20/07/21 | 4,928 | Medline only |
| S10: “Cancer support” | 20/07/21 | 1,350 | Medline only |
| S11: “Cancer care*” | 20/07/21 | 26,976 | Medline only |
| **S12: S1 OR S2 OR S3 OR S4 OR S5 OR S6 OR S7 OR S8 OR S9 OR S10 OR S11** | **20/07/21** | **280,235** | **Medline only** |
| S13: “Web*” | 20/07/21 | 287,832 | Medline only |
| S14: “Internet*” | 20/07/21 | 113,871 | Medline only |
| S15: “Online*” | 20/07/21 | 150,659 | Medline only |
| S16: “Digital*” | 20/07/21 | 182,713 | Medline only |
| **S17: S13 OR S14 OR S15 OR S16** | **20/07/21** | **650,510** | **Medline only** |
| S18: “User experience*” | 20/07/21 | 2,428 | Medline only |
| S19: “Usability” | 20/07/21 | 15,671 | Medline only |
| S20: “Functionality” | 20/07/21 | 66,353 | Medline only |
| S21: “Design” | 20/07/21 | 1,135,342 | Medline only |
| S22: “Interaction” | 20/07/21 | 876,907 | Medline only |
| S23: “Development” | 20/07/21 | 3,148,188 | Medline only |
| S24: “User testing” | 20/07/21 | 350 | Medline only |
| **S25: S18 OR S19 OR S20 OR S21 OR S22 OR S23 OR S24** | **20/07/21** | **4,858,310** | **Medline only** |
| **S26: S12 AND S17 AND S25** | **02/08/21** | **2,452** | **Medline only** |



For peer review only - http://bmjopen.bmj.com/site/about/guidelines.xhtml

## Appendix 2 Adapted Data Extraction Form

| | |
|---|---|
| Review title or ID | |
| Study ID *(surname of first author and year first full report of study was published e.g. Smith 2001)* | |
| Report ID | |
| Report ID of other reports of this study including errata or retractions | |
| Notes | |

## General Information

| | |
|---|---|
| Date form completed *(dd/mm/yyyy)* | |
| Name/ID of person extracting data | |
| Reference citation | |
| Study author contact details | |
| Publication type *(e.g. full report, abstract, letter)* | |
| Notes: | |

For peer review only

## Study eligibility

| **Study Characteristics** | **Eligibility criteria** *(Insert inclusion criteria for each characteristic as defined in the Protocol)* | **Eligibility criteria met?** **Yes** | **No** | **Unclear** | **Location in text or source** *(pg & ¶/fig/table/other)* |
|---|---|---|---|---|---|
| **Type of study** | Quantitative design | ☐ | ☐ | ☐ | |
| | Qualitative design | ☐ | ☐ | ☐ | |
| | Mixed methods design | ☐ | ☐ | ☐ | |
| | Other (Please specify) | | | | |
| **Participants** | Cancer (Specify what type) | ☐ | ☐ | ☐ | |
| | Caregiver | ☐ | ☐ | ☐ | |
| | Friends/family | ☐ | ☐ | ☐ | |
| **Types of intervention** | Website/Internet-based cancer support programme | ☐ | ☐ | ☐ | |



For peer review only - http://bmjopen.bmj.com/site/about/guidelines.xhtml

| | | | | | |
|---|---|---|---|---|---|
| **Type of data (quant and/or qual)**<br><br>(Reports primary data on any of the following) | User experience | ☐ | ☐ | ☐ | |
| | Usability | ☐ | ☐ | ☐ | |
| | Functionality | ☐ | ☐ | ☐ | |
| | Design on web | ☐ | ☐ | ☐ | |
| | Internet-based support living with and affected by cancer. | ☐ | ☐ | ☐ | |
| INCLUDE ☐ | | | EXCLUDE ☐ | | |
| Reason for exclusion | | | | | |
| Notes: | | | | | |

**DO NOT PROCEED IF STUDY EXCLUDED FROM REVIEW**

## Characteristics of included studies

### Methods

| | **Descriptions as stated in report/paper** | **Location in text or source** *(pg & ¶/fig/table/other)* |
|---|---|---|
| **Aim of study** *(e.g. efficacy, equivalence, pragmatic)* | | |
| **Participants** *(e.g. Type of cancer, caregiver role, family and friend's role)* | | |
| **Design** *(e.g. parallel, crossover, non-RCT, exploratory)* | | |

| | | | |
|---|---|---|---|
| **Outcomes** *(details of primary data e.g. user experience, usability, functionality, design etc )* | | | |
| **Start/End date** | | | |
| **Ethical approval needed/ obtained for study** | ☐ Yes ☐ No ☐ Unclear | | |
| **Notes:** | | | |

For peer review only

| | |
|---|---|
| Review title or ID | Hodgkin lymphoma survivor wellness: Development of a web-based intervention. |
| Study ID *(surname of first author and year first full report of study was published e.g. Smith 2001)* | Amweg et al. (2020) |
| Report ID | 10.1188/20.CJON.284-289 |
| Report ID of other reports of this study including errata or retractions | N/A |
| Notes: N/A | |

## General Information

| | |
|---|---|
| Date form completed *(dd/mm/yyyy)* | 12/10/2021 |
| Name/ID of person extracting data | SC |
| Reference citation | Amweg LN, McReynolds J, Lansang K, Jones T, Snow C, Berry DL, Partridge AH, Underhill-Blazey ML. Hodgkin Lymphoma Survivor Wellness: Development of a Web-Based Intervention. Clin J Oncol Nurs. 2020 Jun 1;24(3):284-289. doi: 10.1188/20.CJON.284-289. PMID: 32441674. |
| Study author contact details | Could not access |
| Publication type *(e.g. full report, abstract, letter)* | Full report |
| Notes: N/A | |

## Study eligibility

| **Study Characteristics** | **Eligibility criteria** *(Insert inclusion criteria for each characteristic as defined in the Protocol)* | **Eligibility criteria met?** Yes | No | Unclear | **Location in text or source** *(pg & ¶/fig/table/other)* |
|---|---|---|---|---|---|
| **Type of study** | Quantitative design | ☐ | ☐ | ☐ | |
| | Qualitative design | ☐ | ☐ | ☐ | |
| | Mixed methods design | ☒ | ☐ | ☐ | Page 1 – Abstract<br>Page 2 – methods – Focus groups – Development and usability testing |
| | Other (Please specify) | | | | |

For peer review only

| | | | | | |
|---|---|---|---|---|---|
| **Participants** | Cancer (Specify what type) | ☒ | ☐ | ☐ | Page 2 – Participant identification and recruitment |
| | Caregiver | ☐ | ☐ | ☐ | |
| | Friends/family | ☐ | ☐ | ☐ | |
| **Types of intervention** | Website/Internet-based cancer support programme | ☒ | ☐ | ☐ | Page 2 – Design and setting |

| | | | | | |
|---|---|---|---|---|---|
| **Type of data (quant and/or qual)** (Reports primary data on any of the following) | User experience/Needs/Preferences | ☒ | ☐ | ☐ | Page 2 – Focus groups |
| | Usability/Acceptability/Feasibility | ☒ | ☐ | ☐ | Page 2 – Development and individual usability testing |
| | Functionality | ☐ | ☐ | ☐ | |
| | Design on web | ☐ | ☐ | ☐ | |
| | Internet-based support living with and affected by cancer. | ☐ | ☐ | ☐ | |
| INCLUDE ☒ | | | EXCLUDE ☐ | | |
| Reason for exclusion | | | | | |

Notes:

The aim of this article was to adapt and evaluate a previously developed survivorship care website for Hodgkin Lymphoma survivors.

A mixed methods design broken down into phases

1) Focus group interviews – user needs
2) Web design and user testing – usability

**DO NOT PROCEED IF STUDY EXCLUDED FROM REVIEW**

For peer review only

# Characteristics of included studies

## Methods

| | Descriptions as stated in report/paper | | | | Location in text or source *(pg & ¶/fig/table/other)* |
|---|---|---|---|---|---|
| **Aim of study** *(e.g. efficacy, equivalence, pragmatic)* | The aim of this article was to adapt and evaluate a previously developed survivorship care website for Hodgkin Lymphoma survivors. | | | | Page 1 – Abstract<br>Page 2 – Introduction – paragraph 2 |
| **Participants** *(e.g. Type of cancer, caregiver role, family and friend's role)* | Hodgekins Lymphoma survivors | | | | Page 2 – Methods – Participant identification and recruitment |
| **Design** *(e.g. parallel, crossover, non-RCT, exploratory)* | Mixed methods user centred design – two phases<br>1) Focus groups<br>2) Development and individual usability testing | | | | Page 2 – Methods – Focus groups – Development and usability testing |
| **Outcomes** *(details of primary data e.g. user experience, usability, functionality, design etc )* | User needs/preferences and usability of web-resource | | | | Page 2 – Methods – Focus groups – Development and usability testing |
| **Start/End date** | | | | | |
| **Ethical approval needed/ obtained for study** | ☒ Yes | ☐ No | ☐ Unclear | The Dana-Farber/Harvard Cancer Centre Institutional Review Board approved all study procedures. The study was conducted from April 2017 through December 2018. | Page 2 – Methods – Design and setting. |

For peer review only

**Notes:**

For peer review only

BMJ Open

# A rapid systematic review on developing web-based interventions to support people affected by cancer.

| | |
|---|---|
| Journal: | *BMJ Open* |
| Manuscript ID | bmjopen-2022-062026.R1 |
| Article Type: | Original research |
| Date Submitted by the Author: | 16-Jun-2022 |
| Complete List of Authors: | Cooke, Samuel; University of Lincoln, School of Health and Social Care<br>Nelson, David; University of Lincoln, Lincoln International Institute for Rural Health; Macmillan Cancer Support<br>Green, Heidi; University of Lincoln, School of Health and Social Care<br>McPeake, Kathie; Macmillan Cancer Support; NHS Lincolnshire Clinical Commissioning Group<br>Gussy , Mark ; University of Lincoln, Lincoln International Institute for Rural Health<br>Kane, R; University of Lincoln, School of Health and Social Care |
| <b>Primary Subject Heading</b>: | Oncology |
| Secondary Subject Heading: | Health informatics, Public health |
| Keywords: | PUBLIC HEALTH, ONCOLOGY, Health informatics < BIOTECHNOLOGY & BIOINFORMATICS, World Wide Web technology < BIOTECHNOLOGY & BIOINFORMATICS |
| | |

SCHOLARONE™
Manuscripts

*I, the Submitting Author has the right to grant and does grant on behalf of all authors of the Work (as defined in the below author licence), an exclusive licence and/or a non-exclusive licence for contributions from authors who are: i) UK Crown employees; ii) where BMJ has agreed a CC-BY licence shall apply, and/or iii) in accordance with the terms applicable for US Federal Government officers or employees acting as part of their official duties; on a worldwide, perpetual, irrevocable, royalty-free basis to BMJ Publishing Group Ltd ("BMJ") its licensees and where the relevant Journal is co-owned by BMJ to the co-owners of the Journal, to publish the Work in this journal and any other BMJ products and to exploit all rights, as set out in our licence.*

*The Submitting Author accepts and understands that any supply made under these terms is made by BMJ to the Submitting Author unless you are acting as an employee on behalf of your employer or a postgraduate student of an affiliated institution which is paying any applicable article publishing charge ("APC") for Open Access articles. Where the Submitting Author wishes to make the Work available on an Open Access basis (and intends to pay the relevant APC), the terms of reuse of such Open Access shall be governed by a Creative Commons licence – details of these licences and which Creative Commons licence will apply to this Work are set out in our licence referred to above.*

*Other than as permitted in any relevant BMJ Author's Self Archiving Policies, I confirm this Work has not been accepted for publication elsewhere, is not being considered for publication elsewhere and does not duplicate material already published. I confirm all authors consent to publication of this Work and authorise the granting of this licence.*

For peer review only

# A rapid systematic review on developing web-based interventions to support people affected by cancer.

Samuel Cooke[1], David Nelson[2,3], Heidi Green[1], Kathie McPeake[3,4], Mark Gussy[2], Ros Kane[1]

[1] School of Health and Social Care, University of Lincoln, UK.

[2] Lincoln International Institute for Rural Health, University of Lincoln, UK.

[3] Macmillan Cancer Support, London, UK.

[4] NHS Lincolnshire Clinical Commissioning Group

Correspondence to Samuel Cooke, University of Lincoln, School of Health and Social Care, Brayford Way, Brayford Pool, Lincoln, LN6 7TS Email: scooke@lincoln.ac.uk

Word Count: 3856



For peer review only - http://bmjopen.bmj.com/site/about/guidelines.xhtml

# ABSTRACT

**Objective:** To systematically identify and explore the existing evidence to inform the development of web-based interventions to support people affected by cancer (PABC).

**Design:** A rapid review design was employed in accordance with the guidance produced by the Cochrane Rapid Reviews Methods Group and reported using the PRISMA checklist. A rapid review was chosen due to the need for a timely evidence synthesis to underpin the subsequent development of a digital resource (Shared Lives: Cancer) as part of an ongoing funded project.

**Methods and outcomes:** Keyword searches were performed in MEDLINE to identify peer-reviewed literature that reported primary data on the development of web-based interventions designed to support PABC. The review included peer reviewed studies published in English with no limits set on publication date or geography. Key outcomes included any primary data that reported on the design, usability, feasibility, acceptability, functionality, and user experience of web-based resource development.

**Results:** Ten studies were identified that met the pre-specified eligibility criteria. All studies employed an iterative, co-design approach underpinned by either quantitative, qualitative, or mixed methods. The findings were grouped into the following overarching themes (1) exploring current evidence, guidelines, and theory, (2) identifying user needs and preferences, and (3) evaluating the usability, feasibility, and acceptability of resources. Resources should be informed by the experiences of a wide range of end-users taking into consideration current guidelines and theory early in the design process. Resource design and content should be developed around the user's needs and preferences and evaluated through usability, feasibility, or acceptability testing using quantitative, qualitative, or mixed methods.

**Conclusion:** The findings of this rapid review provide novel methodological insights into the approaches used to design web-based interventions to support PABC. Our findings have the potential to inform and guide researchers when considering the development of future digital health resources.

**Trial registration:** The review protocol was registered on the Open Science Framework (osf.io/ucvsz).


For peer review only

## STRENGTHS AND LIMITATIONS OF THIS STUDY

- This review provides a rapid, yet comprehensive synthesis of the current evidence to support the time-sensitive decision making for the development and implementation of a novel digital resource (Shared Lives: Cancer) to help support PABC.

- This rapid review, whilst streamlined, was conducted using a systematic methodology, following rigorous reporting guidelines to ensure transparency and reproducibility.

- Whilst considered a key part of the knowledge synthesis 'family', rapid review methods are not subject to the same robustness as a full systematic review and are more vulnerable to bias and error.

- Due to time constraints, database searches were restricted to one database only and no formal quality assessment was performed on the included studies.

For peer review only

# INTRODUCTION

Improvements in cancer screening, early detection, diagnostic methods, and treatment are resulting in an increasing number of people living with and beyond cancer.[1-4] Globally, there were an estimated 18.1 million new diagnoses in 2018.[5] In the UK, it is estimated that 4 million people will be living with and beyond cancer by 2030.[6] As services have expanded to support the continuing rise in cancer incidence, so too have the complexities in delivering care.[7-9] This is epitomised by the changes in the way cancer care has been implemented over recent decades, which in the UK for example, now involves a multitude of bodies responsible for purchasing, commissioning, delivering, and regulating services.[8,10]

To ensure the provision of future cancer services adapts to changes in health needs, medical advances, and societal developments, NHS England implemented a long-term plan in which digital health technologies are central.[11] Digital health technologies have become an important tool in cancer care with the potential to revolutionise patient data, transform patient experiences, improve patient recovery, and improve the access, integration, and personalisation of care.[9-12] Evidence suggests that individuals living with and beyond cancer are engaging with digital health technologies now more than ever[13-16] and are using them to frequently access online health information as well as virtual support groups and forums.[17,18]

The rapid growth of internet use has led to a substantial increase in the number of web-based interventions to support PABC, including a wide range of educational and psychosocial platforms,[19-21] social media sites,[22] mobile applications,[22,23] and digital health interventions that focus on specific health behaviours e.g. physical activity and diet.[24] Whilst previous reviews have focused predominantly on the evaluation of web-based interventions, there remains little evidence documenting the developmental (design, usability, feasibility, acceptability, functionality, and user experience) processes of web-based interventions in cancer populations. This review assumes a novel approach by exploring and synthesising the academic literature that reports on the development of web-based resources that support PABC. This will explicitly include resources designed to support the physical, mental, and social consequences of cancer.



For peer review only - http://bmjopen.bmj.com/site/about/guidelines.xhtml

The findings will be used to directly inform the development of a novel web-based resource called (Shared Lives: Cancer),[25,26] that aims to support PABC through making qualitative research data on lived cancer experience publicly available and freely accessible.

This rapid review aims to:

- Identify and map the peer-reviewed academic evidence that reports primary data concerning the development of web-based interventions for supporting PABC.
- Collate and report on the academic evidence with a view to informing web-based interventions for supporting PABC.

# METHODS

This study used a rapid review approach adhering to the recently published guidance from the Cochrane Rapid Reviews Methods Group and for reporting used the Preferred Reporting Item for Systematic Reviews and Meta-Analyses (PRISMA) checklist, see supplementary material (S1). Rapid reviews are now considered a key component of the knowledge synthesis family alongside systematic reviews, scoping reviews and realist reviews. They provide a streamlined, efficient, and pragmatic approach to evidence synthesis.[27] In summary, rapid reviews are a form of evidence synthesis in which components of the systematic review process are simplified, with a view to producing findings in a timely manner.[28,29] Still, rapid reviews must remain systematic in their approach and have a duty to report their methods in a transparent manner making sure they are clear about deviations or omissions from the PRISMA criteria. This review was conducted over a 4-month period (July 2021 – October 2021). The study protocol has been registered on the Open Science Framework (osf.io/ucvsz) to promote reproducibility and facilitate methodological transparency, see supplementary material (S2).

**Ethics Approval**

Not applicable/No human participants included.

**Patient and Public Involvement**

No patient involvement.



For peer review only - http://bmjopen.bmj.com/site/about/guidelines.xhtml

## Search Strategy

Keyword searches together with Boolean operators (OR and AND) and truncation (*) were used to locate relevant peer-reviewed literature on the development of web-based support that is delivered to PABC. Due to the need to produce findings in a timely manner, database searches were limited to one database which is considered acceptable for a rapid systematic review. MEDLINE was searched as it is the leading full-text database of biomedical and health journals. The primary search strategy and syntax was developed and refined by three members of the review team (SC, DN, HG). All database searches were supplemented by Google Scholar searches in addition to forward and backward citation tracking on all relevant articles. Database searches were continually updated to identify and incorporate the most up to date evidence where appropriate.

To identify PABC the following keywords were used: “cancer surviv*” or “living with cancer” or “living with and beyond cancer” or “cancer patient*” or “patients with cancer” “people affected by cancer” or “oncology patient” or “cancer experience*” or “cancer management” or “cancer support” or “cancer care*”. To identify web-based support and interventions the following keywords were used: “web*” or “internet*” or “online*” or “digital*”. To search literature on user experience the following keywords were used: “user experience*” or “usability” or “functionality” or “design” or “interaction” or “development” or “user testing”. The search strategy for MEDLINE can be found in supplementary material (S2).

All retrieved records were collated and stored using Endnote referencing software (EndNote X9, Clarivate Analytics, Philadelphia, USA). The titles and abstracts were screened against the eligibility criteria by one reviewer (SC). Where there was uncertainty about the inclusion of an article after title and abstract screening the first author (SC) discussed this with the second author (DN) to reach a final decision. Following title and abstract screening, the remaining articles were independently screened by full text, for inclusion by two reviewers (SC and DN), with any disagreements again resolved through discussion.

## Eligibility Criteria



For peer review only - http://bmjopen.bmj.com/site/about/guidelines.xhtml

Inclusion criteria

Peer-reviewed publications were selected for inclusion in this review if they met the following pre-defined eligibility based on the PICOT approach. **Population:** Adults (aged 18+), all genders, people living with cancer or affected by cancer, caregivers, any geographical location. **Intervention:** Website-based cancer support resources. **Comparator:** Not applicable. **Outcomes:** Reports primary data on the design, usability, feasibility, acceptability functionality, or user and developer experience of web-based support for PABC. **Type:** Reports empirical research data using either quantitative, qualitative, or mixed methods design. Only publications written in English language were included.

Exclusion criteria

Peer-reviewed publications were excluded based on the following exclusion criteria. **Population:** Non-adult population (under the age of 18). **Intervention:** Support programmes that focus solely on mobile and digital apps, E-learning programmes or interventions (self-directed and practitioner/professionally led), social media or networking sites. **Comparator:** Not applicable. **Outcomes:** No primary data reported on the design, usability, feasibility, acceptability, functionality, or user experiences of web-based support for PABC. **Type**: Systematic reviews or literature reviews, editorials, commentaries, opinion pieces, case series, or reports.

## Data abstraction

Data were extracted using an adapted Cochrane Data Extraction Template, see supplementary (S3). One reviewer (SC) undertook data extraction for each full text article with cross checking taking place by a second reviewer (DN). Study characteristics were extracted from each study based upon (1) study methods (e.g. aims/objectives, study design, participants, outcomes) (2) details on the web-based intervention/support and (3) study findings (details of all relevant data concerning user experience, needs, preferences, usability, acceptability ,feasibility, functionality, and design).

## Quality assessment

The focus of this rapid review is on identifying and exploring the literature on the development of web-based support that is delivered to PABC therefore, a quality assessment of included



For peer review only - http://bmjopen.bmj.com/site/about/guidelines.xhtml

articles was not deemed appropriate. The omission of a quality assessment was in line with the methodological approach taken by other rapid systematic reviews where the focus is on producing evidence quickly.[30]

### Data synthesis and analysis

The review included a wide range of study designs that utilised quantitative, qualitative, and mixed methodologies. To identify and map the evidence on the development of web-based interventions for supporting PABC we tabulated the results. This was then accompanied by a narrative summary where comments on the similarities and dissimilarities within data were made. Due to the wide heterogeneity of the design and outcomes of included studies, as well as the considerable amount of qualitative data, a formal statistical meta-analysis was not conducted; however, the findings were synthesised narratively.

## RESULTS

### Search results

The search of MEDLINE database provided a total of 2,446 distinct citations with an additional 6 identified through secondary sources, see Figure 1. After reviewing for title and abstract, 2,439 did not meet the pre-specified eligibility criteria. The remaining 13 citations were reviewed for full text and examined in detail for inclusion in this review. Three did not meet the pre-specified inclusion criteria as these were self-help, psychological, and educational supportive interventions. The resource the team are creating (Shared Lives: Cancer) cannot be classified as a self-help, psychological, or educational intervention, it exists primarily as a stand-alone website that the public can browse and interact with at their convenience. Therefore, we needed evidence directly in line with this approach to inform our own work and so consequently these articles were excluded. Overall, 10 studies met the pre-defined eligibility criteria that focused on the development of web-based tools to support PABC.

### Study Characteristics

The ten articles were published between 2012-2020 and were undertaken in Australia,[31] Belgium,[32] Vietnam,[33] United Kingdom,[34-37] and the United States of America.[38-40] Five studies focused



For peer review only - http://bmjopen.bmj.com/site/about/guidelines.xhtml

For peer review only

on people with specific cancer types including survivors of Hodgkin Lymphoma,[38] patients with experience of gynaecological cancers,[34] survivors of oral cancer,[39] and patients and survivors of breast cancer,[32,40] whilst four studies,[31,35,36] included patients with experience of a range of cancer types. Some studies also included family caregivers,[39] intimate partners,[32] healthcare professionals,[33,34,36,38] and researchers[34] alongside people with lived cancer experience. One of the included studies collected data with carers of people with cancer and health professionals only.[37]

All studies employed an iterative, co-designed methodological approach for the development of web-resources to support PABC. Two of the studies employed a mixed methods research design,[38,40] six utilised both quantitative and qualitative methods,[31,32,35-37,39] and two articles used solely qualitative methods.[33,34] Four studies explored user needs and preferences using focus groups,[32,38] discussion workshops,[33,34] semi-structured interviews,[33,39] and questionnaires.[32] Three articles explored preferences around the design of the web-based resources using discussion workshops,[34,37] and interviews.[31] Seven studies evaluated the usability and/or acceptability of web resources using 'think aloud' cognitive interviews,[36,38-40] focus groups,[35,36] semi-structured interviews,[40] structured interviews,[35] acceptability E-scales,[38] readiness scales,[31] website tracking,[31,37] and online surveys.[31,40] One study evaluated the feasibility of web-resources using a combination of surveys, questionnaires, and structured interviews,[35] and another carried out user testing (separate to usability testing) via interviews and evaluation surveys.[39] See Table 1 for further details of the characteristics of included studies.



For peer review only - http://bmjopen.bmj.com/site/about/guidelines.xhtml

## Table 1. Characteristics of included studies

| Study (Country) | Population | Methods/Design | Key Outcomes |
|---|---|---|---|
| Amweg et al. (2020)[38]<br><br>(USA) | Hodgkin Lymphoma Survivors n=10<br><br>Healthcare professionals n=9 | Mixed methods, user-centred design<br><br>Two phases:<br>**1)** Focus groups<br>**2)** Usability testing (cognitive interviews and acceptability E-scale) | **User needs and preferences** (Phase 1)<br>Feedback on participants' specification preferences of website<br><br>**Usability** (Phase 2)<br>Feedback of preferences and experience of using website<br>Acceptability of website |
| Ashmore et al. (2020)[34]<br><br>(UK) | Gynaecological cancer patients n=5<br><br>Healthcare professionals n=5<br><br>Researchers n=3 | Qualitative, multi-disciplinary co-creation approach<br><br>Four discussion workshops<br>**1)** Establish understanding of available support and treatment<br>**2)** Establish key areas for support<br>**3)** Website design and requirements<br>**4)** Review of initial resource | **User needs and preferences** (Workshop 1 & 2)<br>Establish understanding of available support and treatment<br>Establish key areas of support<br><br>**Website development** (Workshop 3 & 4)<br>Development of initial resource through creation of a design brief 'wish list'<br>Review of the design of initial resource and identification of recommendations for design team |
| Badr et al. (2016)[39]<br><br>(USA) | Oral cancer survivors n=16<br><br>Family caregiver n=12 | Quantitative and qualitative user-centred design<br><br>Three phases:<br>**1)** Qualitative needs assessment (semi-structured interviews)<br>**2)** Prototype development<br>**3)** Formative evaluation (usability testing - 'think aloud' interviews and user testing – interviews and survey) | **Preferences** (Phase 1)<br>Feedback on the unmet needs and preferences for website<br><br>**Website development** (Phase 2)<br>Development of website prototype<br><br>**Usability/User testing** (Phase 3)<br>Identify navigational difficulties of website<br>Identify participants' experiences of using website<br>Evaluation survey (attractiveness, controllability, efficiency, intuitiveness, learnability) |
| Bartlett et al. (2012)[35]<br><br>(UK) | Cancer patients n=259<br><br>(Breast, Colorectal, Germ cell, Gynaecology, Haematology, Kidney, Prostate, Sarcoma, Upper gastrointestinal) | Quantitative and qualitative user-centred design<br><br>Three phases:<br>**1)** Website design (focus groups and interviews)<br>**2)** Computer and internet survey usage<br>**3)** Crossover study (questionnaires and structured interviews) | **Usability** (Phase 1)<br>Patient feedback on initial web resource<br><br>**Feasibility** (Phase 2)<br>Socio-demographics<br>Computer and internet usage<br><br>**Usability/Feasibility/Acceptability** (Phase 2)<br>Web resource activity tracking<br>Usability and acceptability feedback |
| Bradbury et al. (2019)[36]<br><br>(UK) | Cancer patients n=32<br><br>(Breast, colorectal, and prostate cancer patients)<br><br>Supporters of cancer survivors (nurses, GPs, care assistants, cancer charities) n=31 | Quantitative and qualitative evidence, theory, and user-centred approach<br><br>Seven phases:<br>**1)** Scoping review<br>**2)** Guiding principles<br>**3)** Behavioural analysis<br>**4)** Logic model<br>**5)** Prototype of website<br>**6)** Qualitative optimisation study 1<br>**7)** Qualitative optimisation study 2 | **Usability/Acceptability** (Phase 6 and 7)<br>Feedback of participants experience of exploring website, includes participants' likes, dislikes and recommendations for change |
| Fennell et al. (2017)[31]<br><br>(Australia) | Cancer patients n=122<br><br>(Bones, breast, cervix, colorectal/bowel, lymphoma, lung, melanoma, ovaries, prostate, testicular, brain) | Quantitative and qualitative user-centred approach<br><br>Two phases:<br>1) Website development (interviews)<br>2) Acceptability testing (website tracking activity, readiness scale, online survey) | **Design** (Phase 1)<br>Feedback on initial website content and design<br><br>**Usability/Acceptability** (Phase 2)<br>Website usage<br>Website acceptability and perceived impact |



For peer review only - http://bmjopen.bmj.com/site/about/guidelines.xhtml

**Table 1. Continued**

| Study (Country) | Population | Methods/Design | Key Outcomes |
|---|---|---|---|
| Kapoor et al (2018)[40]<br><br>(USA) | Breast cancer patient or survivor n=15 | Mixed methods, evidence, theory, and user-centred approach<br><br>Seven phases:<br>**1)** Literature review and expert panel<br>**2)** Review of current breast cancer survivorship guidelines and plans<br>**3)** Development of decisions<br>**4)** Curation of decisions<br>**5)** Prototype design and development<br>**6)** User feedback (semi-structured interviews)<br>**7)** Usability testing ('think aloud' and semi-structured interviews and online surveys) | **Perceived usefulness** (Phase 6)<br>Identify participants' perception and perceived usefulness of the website<br><br>**Usability** (Phase 7)<br>Identify the overall usability of the website |
| Pauwels et al (2012)[32]<br><br>(Belgium) | Breast cancer survivors n=57<br><br>Intimate partners n=28 | Quantitative and qualitative user-centred design<br><br>Pre and post design (post-questionnaire, website tracking, care needs questionnaire) | **User needs**<br>Assessment of participants' needs for information and support<br><br>**Design**<br>Evaluation of the content and lay-out of the website. Concepts evaluated include user friendly, well-built, interesting, informative, understandable, new, incomplete, irrelevant, unreliable, too extensive, or confusing<br><br>**Usability**<br>Information gathered about participants' use of the website |
| Santin et al (2019)[37]<br><br>(UK) | Cancer carers n=12 | Quantitative and qualitative co-design approach<br><br>Two phases:<br>**1)** Co-design model<br>-Design of website (workshops and meetings)<br>-Development of prototype 1<br>-User testing phase 1 (unstructured feedback sessions)<br>-Refining prototype<br>**2)** User testing<br>-User testing and refinement (semi-structured interviews, web survey, website tracking)<br>-Final development | **Design** (Phase 1)<br>Evaluation and refinement of website design<br><br>**Usability** (Phase 1 & 2)<br>To gather views and experiences of users' interactions with the web-resource.<br><br>Evaluate website use through tracking website activity |
| Santin et al (2020)[33]<br><br>(Vietnam) | Informal cancer carers n=20<br><br>Healthcare professionals n=23 | Qualitative co-design approach<br><br>Two phases:<br>1) Identification of needs (interviews and focus groups)<br>2) Stakeholder verification and refinements (co-design workshops) | **User needs and perspectives** (Phase 1 & 2)<br>Identifying and understanding the needs of informal cancer carers to underpin resource content<br><br>Learn and agree upon shared priorities and resource components between informal cancer carers and healthcare professionals |



For peer review only - http://bmjopen.bmj.com/site/about/guidelines.xhtml

## Overarching themes

The findings from the ten articles were grouped under the following three areas (1) exploring current evidence, guidelines, and theory (2) identifying user needs and preferences, and (3) evaluating the usability, feasibility, and acceptability of resources.

Exploring current evidence, guidelines, and theory

Bradbury et al.[36] conducted a rapid scoping review to identify the barriers and facilitators to intervention success including the participants needs and attributes and intervention components. Synthesised evidence from the review informed key design objectives including employing an approach that promotes well-being, ensuring the appropriate promotion of behaviour change, providing easy, timely and tailored information, and ensuring an efficient design. These findings were used to establish intervention guiding principles and inform the behavioural analysis and logic model that would underpin resource development. Similarly, Kapoor et al.[40] conducted a literature review to identify the needs of breast cancer survivors to assist in informing web-resource development. The findings, combined with input from an expert panel, helped to identify core functions to be incorporated into the design of a prototype resource including recording and tracking of quality life indicators, recording user-reported treatment-related symptoms, viewing breast cancer related medical history, viewing scheduled follow-up visits, and generating and displaying customised alerts related to symptoms and quality of life issues. Other studies also reported reviewing patient websites and performing literature reviews but were not explicit on how findings informed web-resource development.[31,37]

In addition to reviewing the available literature, studies also reviewed existing guidelines and theory to inform web-resource development. Kapoor et al.[40] conducted a comprehensive review of current breast cancer survivorship guidelines and existing survivorship plans which were used to inform the inclusion of key support information within the web-resource. Badr et al.[39] explored the best practices underpinning the management for oral and swallowing complications following radiotherapy, whilst also reviewing national healthy lifestyle guidelines for cancer survivors and evidence surrounding the self-determination theory. The findings were used to develop a prototype web-resource that specifically focused on promoting survivor and caregiver autonomy, competence, and relatedness; by providing



For peer review only - http://bmjopen.bmj.com/site/about/guidelines.xhtml

tailored information, skill-building education, and support services. Other studies also reported reviewing clinical practice guidelines alongside reviewing the academic literature.[31]

### Identifying the needs and preferences for resources

Participants of the included studies emphasised the need for resources that provide comprehensive information on cancer management and survivorship.[32-34,39] The need for clear information on survivorship care with a specific focus on physical, psychosocial, psychosexual, and emotional well-being was identified;[32,34,38] in addition to information on adjusting to 'new normal', returning to work, financial management, and lifestyle advice.[32,34,39] The inclusion of practical advice and information on the side effects of cancer treatments was viewed as essential[34,39] and participants expressed the need to learn from other survivors and carers through shared experiences and self-care strategies.[33,34,39] Concerns were raised by survivors regarding the risk of secondary cancers and how to communicate with family about experiences of cancer survivorship.[34] The inclusion of a 'Frequently Asked Questions' page was also proposed to ensure a safe space for users to search for specific information.[33,34]

Reported discussions between healthcare professionals focused on the need to ensure resources can be integrated easily into existing digital systems and are accessible across clinical specialities.[38] It was also considered important that participants did not view resources as a substitute for clinical care[38] and that information on family/carer support be included.[34] Caregivers expressed the need for emotional and supportive information on how to cope with cancer in addition to information on cancer side effects and lifestyle advice.[32,33,39] Concerns were also raised regarding the fear of reoccurrence and the need for specific self-care information and better family communication for carers.[39] Caregivers also discussed the inclusion of information regarding cancer causes and treatment, pain management, hospital administration and treatment processes, hospital daily living, and signposting to skills training.[33,39]



For peer review only - http://bmjopen.bmj.com/site/about/guidelines.xhtml

Evaluating the usability, feasibility, and acceptability of resources

Studies explored the usability, feasibility, and acceptability of resources by qualitatively drawing upon the users' positive and negative experiences of web-resource interaction. Users viewed web-resources positively and valued their use in providing centralised, easily accessible information to support and facilitate survivorship care.[36-38,40] The content included within web-resources were regarded as useful in managing the consequences of cancer and was viewed as a credible source of information due to its development by trusted experts.[36,37,40] Accessing information through web-resources and video formats was perceived as less burdensome than written information and allowed users to easily access advice.[37] Resource features including providing useful website links, being able to access medical history, and tracking quality life indicators was also perceived as valuable components of web-resources.[40]

Whilst web-resources did provide easy access to information, the content of web-resources was considered impersonal with users expressing the need for more customised and prioritised information[35,37,38,40] that was representative of all genders.[37] Web-resources were found to be too complex with users experiencing difficulties in navigating and understanding the purpose of certain web-features highlighting the importance in developing simple and user-friendly web-resources.[35,38,39] Issues with web-resource design were also experienced with users emphasising the need for more appealing web-designs that use appropriate colour and size of both fonts and paragraphs, include greater cross-links, and incorporate much clearer navigational features.[31,38,39]

Studies also evaluated the usability, feasibility and acceptability of web-resources using a range of quantitative methods. A common approach identified was the use of Likert scale style questionnaires and surveys.[32,39,40] For example, Badr et al.[39] reported an overall resource usability score of 80/100 with individual areas rates as attractiveness (4.0/5), controllability (4.2/5), efficiency (4.1/5), intuitiveness (3.9/5), and learnability (3.8/5). Amweg et al.[38] employed an acceptability E-scale to objectively identify web-resource acceptability reporting an overall score of 29.8 (a score of <24 was considered an indicator of web-resource acceptability). Other studies also used descriptive



For peer review only - http://bmjopen.bmj.com/site/about/guidelines.xhtml

questionnaires and surveys with users rating web-resources as easy to use, useful, relevant, necessary, and likely to return and recommend.[35,37] Studies were also shown to objectively explore website usability using website analytics.[31,32,35,37] For example, Santin et al.[37] reported 2769 unique visits between November 2017 and May 2018 of which 743 were returning visitors. Visitors were shown to access multiple website components including the 'getting through treatment', 'caring for you', 'financial', and 'employment' elements. Peer-led videos were the most frequently accessed content whilst professional led material, supporting children, and the emotional aspects of caring were the least visited.

## DISCUSSION

This rapid review has systematically identified and mapped the peer reviewed academic evidence that reported on primary data concerning the development of web-based interventions for supporting PABC. Our findings highlight the use of user-centred, co-designed methodological approaches that are underpinned by iterative, but not necessarily sequential, development processes. A common approach used to develop web-based resources involved the initial exploration of the current evidence, guidelines, and theory followed by an assessment of user needs and preferences to ensure that web-resources were designed to meet the needs of its users. This was typically proceeded by the evaluation of resources involving usability, feasibility, or acceptability testing using a wide range of quantitative, qualitative, and mixed methods that often fed back into further resource refinement. Whilst previous reviews focus predominantly on evaluating the effectiveness of web-based resources, this rapid review differs in that it provides important and novel insights into the methodological approaches that underpin the development and implementation of web-based resources to support PABC. Our findings have the potential to assist other researchers who are developing digital resources and will be used by the current research team to inform the development of a web-based support platform (Shared Lives: Cancer)[25,26] that aims to make qualitative research data on lived cancer experiences publicly available via an open access searchable website. Specifically, the findings have made the team aware that the development of digital resources should be informed by the experiences of a wide range of end-users and co-developed where possible and appropriate. The design and content of resources should be



For peer review only - http://bmjopen.bmj.com/site/about/guidelines.xhtml

centred around the user's needs and preferences and include resource evaluation as part of an iterative approach through usability, feasibility, or acceptability testing using a range of different methods. Following the launch of Shared Lives: Cancer, the team will continue to collect data on user experience to ensure its design and content is grounded within the needs of its intended audience.

Ensuring the appropriate design of web-based resources is a critical component of website development[41] in which the use of iterative, co-designed methods is strongly advocated,[42,43] especially with respect to cancer care.[44] This is supported by previous evidence that demonstrated the engagement of stakeholders throughout the developmental process ensures that digital tools are firmly grounded within the user's needs, which consequently improves usability and increases user engagement.[45-47] However, there must be an appreciation that users will have varying levels of digital literacy and this needs to be considered when designing and delivering digital resources. Existing research has shown that poor digital literacy is linked with computer anxiety and barriers to internet use amongst PABC.[48,49] Therefore, resources should be accessible and lay friendly to encourage engagement with people who have lower levels of digital literacy. At the same time, there will and continues to be PABC who prefer non-digital support for a variety of reasons. Therefore it is important that face-to-face support is maintained as digital services continue to be widely rolled out as a consequence of both the Covid-19 pandemic and global healthcare policies.

The findings from this review also emphasises the importance of collecting data on usability, feasibility, and acceptability, which are widely considered as important elements when developing web-based resources. An important decision future researchers may face during the ongoing development of digital resources is deciding how these areas will be measure. In line with evidence concerning usability and acceptability testing,[50,51] our findings point towards employing the use of a wide range of quantitative and qualitative methods and where possible should consider a combination of methodologies.[52] Whilst we identify key assessment methods including website analytics, E-scales, questionnaires, 'think aloud' interviews, semi-structured interviews, focus groups and workshops, future research should also consider other methods including more objective and automated methods, especially in the context of usability testing.[50,52]



For peer review only - http://bmjopen.bmj.com/site/about/guidelines.xhtml

The development and implementation of digital tools has enormous potential in supporting future healthcare services through transforming the way individuals engage with services and professionals, advancing efficient care coordination, and allowing individuals to better manage one's health and well-being.[53-55] The use of digital technology is now considered a fundamental element that will underpin many of the proposed changes as part of the NHS long-term plan,[11] including desires to facilitate better care and support for individuals at home through the use of digital health tools. As the NHS looks to transform and adapt over the next decade, it is important to consider digital health technologies as a potential solution to improve and strengthen aspects or cancer care.[44] The findings of the current review provide important methodological insight that should be used to develop emerging digital health technologies that may help transform and support future healthcare services.

A strength of this review is that it allowed for a rapid synthesis of the current evidence needed to provide timely information to inform the decision-making process surrounding the development and implementation of a novel digital support resource (Shared Lives: Cancer)[25,26] as part of an externally funded project. It provides important insight into the methodological approaches used to develop web-based resources which may be used to guide and inform the design of future digital resources. A limitation of the current review was the lack of consistency and uniformity across outcome measurement tools of included studies, making it challenging to compare and interpret findings. Whilst rapid reviews are key in synthesising timely and informative evidence, it is recognised that the accelerated review process is not subject to the same robustness as a full systematic review. The current rapid review used a streamlined review process that restricted literature searches to one database only and omitted the inclusion of assessing risk of bias. We would encourage other researchers who are developing this work further to conduct a full systematic review that also includes a quality assessment of the academic literature. It is therefore acknowledged that the methodology of the current study is less comprehensive and as a consequence the results may be more susceptible to bias and error.

## CONCLUSION

This research adopted a rapid review approach as there is a timely need for an evidence synthesis to support and inform the development of an ongoing project to design an online web-based platform



For peer review only - http://bmjopen.bmj.com/site/about/guidelines.xhtml

(Shared Lives: Cancer).[25,26] The findings of this rapid review provide an important insight into the methodological approaches used to underpin the development of web-based interventions to support PABC. The evidence generated from this review has the potential to inform and guide future research endeavours when considering the development and implementation of digital resources.

## AUTHOR CONTRIBUTIONS

SC, DN, HG and RK conceptualised and designed the review. SC reviewed titles, abstracts, full-text articles and extracted data with all data extraction verified by DN. SC and DN prepared the initial manuscript. HG, RK, KM, and MG reviewed and edited the final manuscript.

## ACKNOWLEDGEMENTS

The authors would like to acknowledge the ongoing support of the East Midlands Cancer Alliance and Macmillan Cancer Support with the development of Shared Lives: Cancer.

## FUNDING STATEMENT

This research was supported by the National Institute for Health Research (NIHR) Clinical Research Network (CRN) East Midlands Targeted Funding Call (Grant number/Award number: N/A).

## COMPETING INTERESTS

The authors declare no competing interests.

## PATIENT CONSENT FOR PUBLICATION

Not applicable.

## DATA SHARING

All data are included within the manuscript and supplementary material.

For peer review only

## REFERENCES

1 Arnold M, Rutherford MJ, Bardot A, et al. Progress in cancer survival, mortality, and incidence in seven high-income countries 1995-2014 (ICBP SURVMARK-2): a population-based study. *Lancet Oncol* 2019;20(11):1493-505. doi: 10.1016/s1470-2045(19)30456-5 [published Online First: 2019/09/16]

2 Hanna TP, King WD, Thibodeau S, et al. Mortality due to cancer treatment delay: systematic review and meta-analysis. *BMJ* 2020;371:m4087. doi: 10.1136/bmj.m4087

3 Loud JT, Murphy J. Cancer Screening and Early Detection in the 21(st) Century. *Seminars in oncology nursing* 2017;33(2):121-28. doi: 10.1016/j.soncn.2017.02.002 [published Online First: 2017/03/23]



For peer review only - http://bmjopen.bmj.com/site/about/guidelines.xhtml

4 Siegel RL, Miller KD, Fuchs HE, et al. Cancer Statistics, 2021. *CA: A Cancer Journal for Clinicians* 2021;71(1):7-33. doi: https://doi.org/10.3322/caac.21654
5 Bray F, Ferlay J, Soerjomataram I, et al. Global cancer statistics 2018: GLOBOCAN estimates of incidence and mortality worldwide for 36 cancers in 185 countries. *CA Cancer J Clin* 2018;68(6):394-424. doi: 10.3322/caac.21492 [published Online First: 2018/09/13]
6 NCRI. Living with and Beyond Cancer 2020 [Available from: https://www.ncri.org.uk/areas-of-interest/living-with-beyond-cancer/ accessed January 2022.
7 Bosanquet N, Sikora K. The economics of cancer care in the UK. *Lancet Oncol* 2004;5(9):568-74. doi: 10.1016/s1470-2045(04)01569-4 [published Online First: 2004/09/01]
8 Macklin-Doherty A. Accountability in the NHS: the impact on cancer care. *Ecancermedicalscience* 2018;12:ed83-ed83. doi: 10.3332/ecancer.2018.ed83
9 ICT. Acheiving world-class cancer outcomes: A strategy for England 2015-2020 2015 [updated 2015. Available from: https://www.iccp-portal.org/system/files/plans/Strategy%20-Final.pdf accessed December 2021.
10 Exarchakou A, Rachet B, Belot A, et al. Impact of national cancer policies on cancer survival trends and socioeconomic inequalities in England, 1996-2013: population based study. *Bmj* 2018;360:k764. doi: 10.1136/bmj.k764 [published Online First: 2018/03/16]
11 NHS. The NHS long term plan 2019 [Available from: https://www.longtermplan.nhs.uk/ accessed December 2021.
12 Kemp E, Trigg J, Beatty L, et al. Health literacy, digital health literacy and the implementation of digital health technologies in cancer care: the need for a strategic approach. *Health Promot J Austr* 2021;32 Suppl 1:104-14. doi: 10.1002/hpja.387 [published Online First: 2020/07/19]
13 Dee EC, Muralidhar V, Butler SS, et al. General and Health-Related Internet Use Among Cancer Survivors in the United States: A 2013-2018 Cross-Sectional Analysis. *J Natl Compr Canc Netw* 2020;18(11):1468-75. doi: 10.6004/jnccn.2020.7591 [published Online First: 2020/11/06]
14 Fareed N, Swoboda CM, Jonnalagadda P, et al. Persistent digital divide in health-related internet use among cancer survivors: findings from the Health Information National Trends Survey, 2003-2018. *J Cancer Surviv* 2021;15(1):87-98. doi: 10.1007/s11764-020-00913-8 [published Online First: 2020/07/17]
15 Castleton K, Fong T, Wang-Gillam A, et al. A survey of Internet utilization among patients with cancer. *Support Care Cancer* 2011;19(8):1183-90. doi: 10.1007/s00520-010-0935-5 [published Online First: 2010/06/18]
16 Eysenbach G. The impact of the Internet on cancer outcomes. *CA Cancer J Clin* 2003;53(6):356-71. doi: 10.3322/canjclin.53.6.356 [published Online First: 2004/07/01]
17 Lleras de Frutos M, Casellas-Grau A, Sumalla EC, et al. A systematic and comprehensive review of internet use in cancer patients: Psychological factors. *Psychooncology* 2020;29(1):6-16. doi: 10.1002/pon.5194 [published Online First: 2019/08/07]
18 Mattsson S, Olsson EMG, Johansson B, et al. Health-Related Internet Use in People With Cancer: Results From a Cross-Sectional Study in Two Outpatient Clinics in Sweden. *Journal of medical Internet research* 2017;19(5):e163-e63. doi: 10.2196/jmir.6830
19 Aapro M, Bossi P, Dasari A, et al. Digital health for optimal supportive care in oncology: benefits, limits, and future perspectives. *Support Care Cancer* 2020;28(10):4589-612. doi: 10.1007/s00520-020-05539-1 [published Online First: 2020/06/14]
20 Escriva Boulley G, Leroy T, Bernetière C, et al. Digital health interventions to help living with cancer: A systematic review of participants' engagement and psychosocial effects. *Psychooncology* 2018;27(12):2677-86. doi: 10.1002/pon.4867 [published Online First: 2018/08/29]
21. McCann L, McMillan KA, Pugh G. Digital Interventions to Support Adolescents and Young Adults With Cancer: Systematic Review. *JMIR Cancer* 2019;5(2):e12071. doi: 10.2196/12071 [published Online First: 2019/08/02]



For peer review only - http://bmjopen.bmj.com/site/about/guidelines.xhtml

22 Charbonneau DH, Hightower S, Katz A, et al. Smartphone apps for cancer: A content analysis of the digital health marketplace. *Digit Health* 2020;6:2055207620905413. doi: 10.1177/2055207620905413 [published Online First: 2020/02/29]

23 Prochaska JJ, Coughlin SS, Lyons EJ. Social Media and Mobile Technology for Cancer Prevention and Treatment. *Am Soc Clin Oncol Educ Book* 2017;37:128-37. doi: 10.1200/edbk_173841 [published Online First: 2017/06/01]

24 Roberts AL, Fisher A, Smith L, et al. Digital health behaviour change interventions targeting physical activity and diet in cancer survivors: a systematic review and meta-analysis. *J Cancer Surviv* 2017;11(6):704-19. doi: 10.1007/s11764-017-0632-1 [published Online First: 2017/08/06]

25 Green H ND, Kane R, McPeake K, Willis R. Developing an online resource to support people affected by cancer through making academic research publicly accessible. National Cancer Research Institute (NCRI) virtual showcase Online, 2020.

26 Green H MK, Nelson D, Cooke S, Kane R, . Shared Lives Cancer: An update on the development of an innovative digital health tool to support those affected by cancer. National Cancer Research Institute (NCRI) cancer conference. Online 2021.

27 Moher D, Stewart L, Shekelle P. All in the Family: systematic reviews, rapid reviews, scoping reviews, realist reviews, and more. *Systematic Reviews* 2015;4(1):183. doi: 10.1186/s13643-015-0163-7

28 Khangura S, Konnyu K, Cushman R, et al. Evidence summaries: the evolution of a rapid review approach. *Systematic Reviews* 2012;1(1):10. doi: 10.1186/2046-4053-1-10

29 Tricco AC, Antony J, Zarin W, et al. A scoping review of rapid review methods. *BMC Medicine* 2015;13(1):224. doi: 10.1186/s12916-015-0465-6

30 Haby MM, Chapman E, Clark R, Barreto J, Reveiz L, Lavis JN. What are the best methodologies for rapid reviews of the research evidence for evidence-informed decision making in health policy and practice: a rapid review. *Health Res Policy Syst*. 2016;14(1):83. doi:10.1186/s12961-016-0155-7

31 Fennell KM, Turnbull DA, Bidargaddi N, et al. The consumer-driven development and acceptability testing of a website designed to connect rural cancer patients and their families, carers and health professionals with appropriate information and psychosocial support. *Eur J Cancer Care (Engl)* 2017;26(5) doi: 10.1111/ecc.12533 [published Online First: 2016/07/14]

32 Pauwels E, Van Hoof E, Charlier C, et al. Design and process evaluation of an informative website tailored to breast cancer survivors' and intimate partners' post-treatment care needs. *BMC research notes* 2012;5:548-48. doi: 10.1186/1756-0500-5-548

33 Santin O, Jenkins C, Nghiem HLP, et al. The development of a web-based resource to provide information and psychosocial support to informal cancer carers in hospitals in Vietnam. *Psychooncology* 2020;29(5):920-26. doi: 10.1002/pon.5368 [published Online First: 2020/02/27]

34 Ashmore LA, Stewart H, Hutton D, et al. Digital support for living with and beyond gynaecological cancer. *Radiography (Lond)* 2020;26(4):e270-e76. doi: 10.1016/j.radi.2020.03.014 [published Online First: 2020/05/23]

35 Bartlett YK, Selby DL, Newsham A, et al. Developing a useful, user-friendly website for cancer patient follow-up: users' perspectives on ease of access and usefulness. *Eur J Cancer Care (Engl)* 2012;21(6):747-57. doi: 10.1111/j.1365-2354.2012.01357.x [published Online First: 2012/04/27]

36 Bradbury K, Steele M, Corbett T, et al. Developing a digital intervention for cancer survivors: an evidence-, theory- and person-based approach. *NPJ Digit Med* 2019;2:85. doi: 10.1038/s41746-019-0163-4 [published Online First: 2019/09/12]

37 Santin O, McShane T, Hudson P, et al. Using a six-step co-design model to develop and test a peer-led web-based resource (PLWR) to support informal carers of cancer patients.



For peer review only - http://bmjopen.bmj.com/site/about/guidelines.xhtml

*Psychooncology* 2019;28(3):518-24. doi: 10.1002/pon.4969 [published Online First: 2019/01/01]
38 Amweg LN, McReynolds J, Lansang K, et al. Hodgkin Lymphoma Survivor Wellness: Development of a Web-Based Intervention. *Clin J Oncol Nurs* 2020;24(3):284-89. doi: 10.1188/20.Cjon.284-289 [published Online First: 2020/05/23]
39 Badr H, Lipnick D, Diefenbach MA, et al. Development and usability testing of a web-based self-management intervention for oral cancer survivors and their family caregivers. *Eur J Cancer Care (Engl)* 2016;25(5):806-21. doi: 10.1111/ecc.12396 [published Online First: 2015/10/29]
40 Kapoor A, Nambisan P. Usability and acceptance evaluation of ACESO: a Web-based breast cancer survivorship tool. *J Cancer Surviv* 2018;12(3):316-25. doi: 10.1007/s11764-017-0670-8 [published Online First: 2018/01/27]
41 Garett R, Chiu J, Zhang L, et al. A Literature Review: Website Design and User Engagement. *Online J Commun Media Technol* 2016;6(3):1-14.
42 Leask CF, Sandlund M, Skelton DA, et al. Framework, principles and recommendations for utilising participatory methodologies in the co-creation and evaluation of public health interventions. *Res Involv Engagem* 2019;5:2. doi: 10.1186/s40900-018-0136-9 [published Online First: 2019/01/18]
43 Skivington K, Matthews L, Simpson SA, et al. A new framework for developing and evaluating complex interventions: update of Medical Research Council guidance. *BMJ* 2021;374:n2061. doi: 10.1136/bmj.n2061
44 Hesse BW, Kwasnicka D, Ahern DK. Emerging digital technologies in cancer treatment, prevention, and control. *Transl Behav Med* 2021;11(11):2009-17. doi: 10.1093/tbm/ibab033 [published Online First: 2021/12/02]
45 Bjerkan J, Hedlund M, Hellesø R. Patients' contribution to the development of a web-based plan for integrated care - a participatory design study. *Inform Health Soc Care* 2015;40(2):167-84. doi: 10.3109/17538157.2014.907803 [published Online First: 2014/05/03]
46 Davenport T, Milton A, Ospina-Pinillos L, et al. Project Synergy research and development cycle: iterative processes of participatory design, user testing, implementation and feasibility testing. *Med J Aust* 2019;211(7):S8-11.
47 LaMonica HM, Davenport TA, Burns J, et al. Technology-Enabled Mental Health Service Reform for Open Arms - Veterans and Families Counselling: Participatory Design Study. *JMIR Form Res* 2019;3(3):e13662. doi: 10.2196/13662 [published Online First: 2019/09/21]
48 Lepore SJ, Rincon MA, Buzaglo JS, et al. Digital literacy linked to engagement and psychological benefits among breast cancer survivors in Internet-based peer support groups. *European journal of cancer care* 2019;28(4):e13134-e34. doi: 10.1111/ecc.13134
49 Hoogland AI, Mansfield J, Lafranchise EA, et al. eHealth literacy in older adults with cancer. *J Geriatr Oncol* 2020;11(6):1020-22. doi: 10.1016/j.jgo.2019.12.015 [published Online First: 2020/01/10]
50 Maramba I, Chatterjee A, Newman C. Methods of usability testing in the development of eHealth applications: A scoping review. *Int J Med Inform* 2019;126:95-104. doi: 10.1016/j.ijmedinf.2019.03.018 [published Online First: 2019/04/29]
51 Sekhon M, Cartwright M, Francis JJ. Acceptability of healthcare interventions: an overview of reviews and development of a theoretical framework. *BMC Health Services Research* 2017;17(1):88. doi: 10.1186/s12913-017-2031-8
52 Zapata BC, Fernández-Alemán JL, Idri A, et al. Empirical Studies on Usability of mHealth Apps: A Systematic Literature Review. *Journal of Medical Systems* 2015;39(2):1. doi: 10.1007/s10916-014-0182-2
53 Manteghinejad A, Javanmard SH. Challenges and opportunities of digital health in a post-COVID19 world. *J Res Med Sci* 2021;26:11-11. doi: 10.4103/jrms.JRMS_1255_20
54 Meskó B, Drobni Z, Bényei É, et al. Digital health is a cultural transformation of traditional healthcare. *mHealth* 2017;3:38-38. doi: 10.21037/mhealth.2017.08.07



For peer review only - http://bmjopen.bmj.com/site/about/guidelines.xhtml

55 Powell J, Newhouse N, Boylan A-M, et al. Digital health citizens and the future of the NHS. *Digital health* 2016;2:2055207616672033-33. doi: 10.1177/2055207616672033
56 Moher D, Liberati A, Tetzlaff J, et al. Preferred reporting items for systematic reviews and meta-analyses: the PRISMA statement. *PLoS Med* 2009;6(7):e1000097. doi: 10.1371/journal.pmed.1000097 [published Online First: 2009/07/22]

**References included within tables**

31 Fennell KM, Turnbull DA, Bidargaddi N, et al. The consumer-driven development and acceptability testing of a website designed to connect rural cancer patients and their families, carers and health professionals with appropriate information and psychosocial support. *Eur J Cancer Care (Engl)* 2017;26(5) doi: 10.1111/ecc.12533 [published Online First: 2016/07/14]
32 Pauwels E, Van Hoof E, Charlier C, et al. Design and process evaluation of an informative website tailored to breast cancer survivors' and intimate partners' post-treatment care needs. *BMC research notes* 2012;5:548-48. doi: 10.1186/1756-0500-5-548
33 Santin O, Jenkins C, Nghiem HLP, et al. The development of a web-based resource to provide information and psychosocial support to informal cancer carers in hospitals in Vietnam. *Psychooncology* 2020;29(5):920-26. doi: 10.1002/pon.5368 [published Online First: 2020/02/27]
34 Ashmore LA, Stewart H, Hutton D, et al. Digital support for living with and beyond gynaecological cancer. *Radiography (Lond)* 2020;26(4):e270-e76. doi: 10.1016/j.radi.2020.03.014 [published Online First: 2020/05/23]
35 Bartlett YK, Selby DL, Newsham A, et al. Developing a useful, user-friendly website for cancer patient follow-up: users' perspectives on ease of access and usefulness. *Eur J Cancer Care (Engl)* 2012;21(6):747-57. doi: 10.1111/j.1365-2354.2012.01357.x [published Online First: 2012/04/27]
36 Bradbury K, Steele M, Corbett T, et al. Developing a digital intervention for cancer survivors: an evidence-, theory- and person-based approach. *NPJ Digit Med* 2019;2:85. doi: 10.1038/s41746-019-0163-4 [published Online First: 2019/09/12]
37 Santin O, McShane T, Hudson P, et al. Using a six-step co-design model to develop and test a peer-led web-based resource (PLWR) to support informal carers of cancer patients. *Psychooncology* 2019;28(3):518-24. doi: 10.1002/pon.4969 [published Online First: 2019/01/01]
38 Amweg LN, McReynolds J, Lansang K, et al. Hodgkin Lymphoma Survivor Wellness: Development of a Web-Based Intervention. *Clin J Oncol Nurs* 2020;24(3):284-89. doi: 10.1188/20.Cjon.284-289 [published Online First: 2020/05/23]
39 Badr H, Lipnick D, Diefenbach MA, et al. Development and usability testing of a web-based self-management intervention for oral cancer survivors and their family caregivers. *Eur J Cancer Care (Engl)* 2016;25(5):806-21. doi: 10.1111/ecc.12396 [published Online First: 2015/10/29]
40 Kapoor A, Nambisan P. Usability and acceptance evaluation of ACESO: a Web-based breast cancer survivorship tool. *J Cancer Surviv* 2018;12(3):316-25. doi: 10.1007/s11764-017-0670-8 [published Online First: 2018/01/27]

**References included within figures**

56 Moher D, Liberati A, Tetzlaff J, et al. Preferred reporting items for systematic reviews and meta-analyses: the PRISMA statement. *PLoS Med* 2009;6(7):e1000097. doi: 10.1371/journal.pmed.1000097 [published Online First: 2009/07/22]



For peer review only - http://bmjopen.bmj.com/site/about/guidelines.xhtml

## Figure legends

**Figure 1.** Study flowchart[56]

For peer review only



For peer review only - http://bmjopen.bmj.com/site/about/guidelines.xhtml

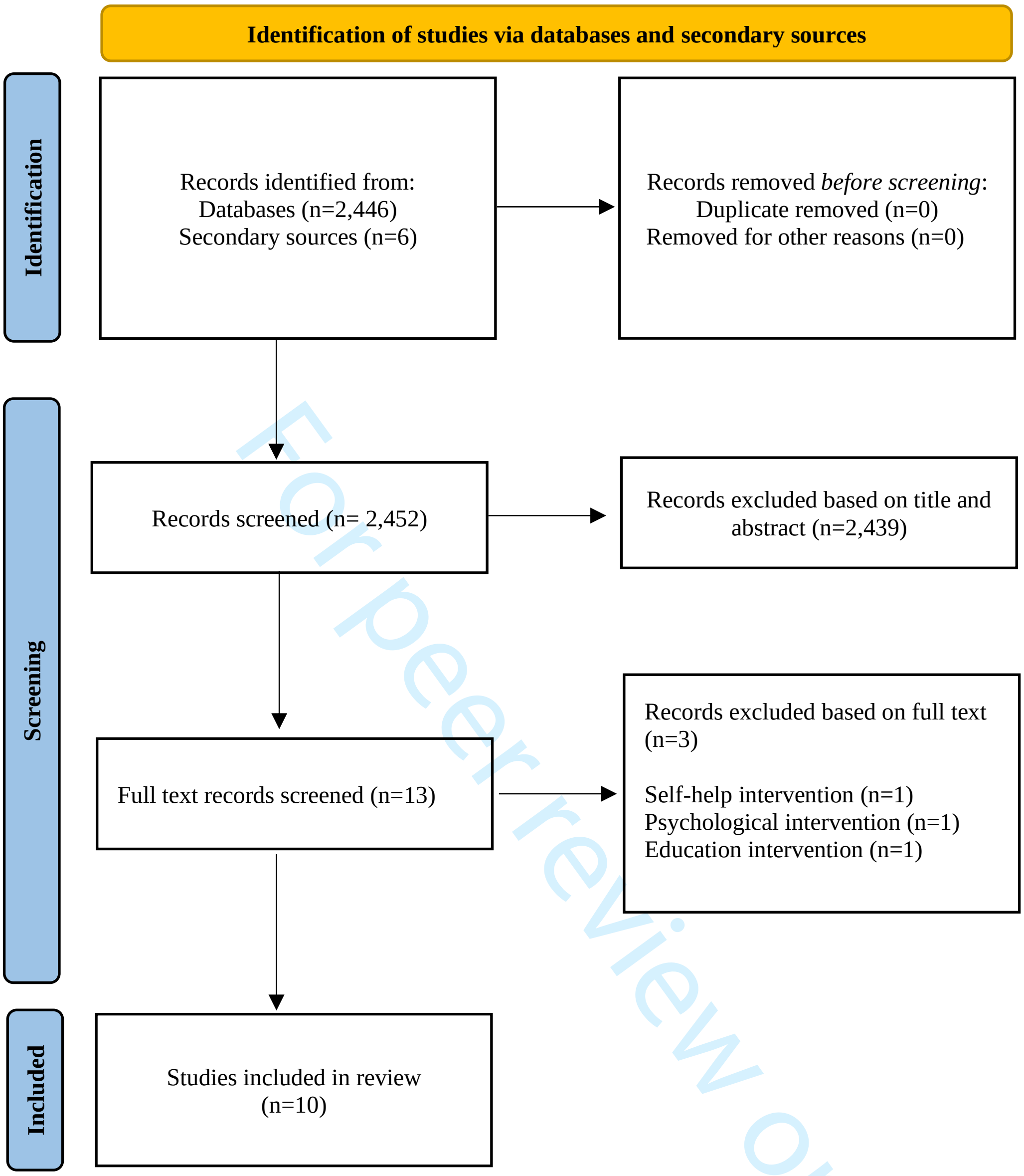


For peer review only

# PRISMA 2020 Checklist

| Section and Topic | Item # | Checklist item | Location where item is reported |
|---|---|---|---|
| **TITLE** | | | |
| Title | 1 | Identify the report as a systematic review. | Page 1 |
| **ABSTRACT** | | | |
| Abstract | 2 | See the PRISMA 2020 for Abstracts checklist. | Page 2 |
| **INTRODUCTION** | | | |
| Rationale | 3 | Describe the rationale for the review in the context of existing knowledge. | Page 4/5 |
| Objectives | 4 | Provide an explicit statement of the objective(s) or question(s) the review addresses. | Page 2/5 |
| **METHODS** | | | |
| Eligibility criteria | 5 | Specify the inclusion and exclusion criteria for the review and how studies were grouped for the syntheses. | Page 7 |
| Information sources | 6 | Specify all databases, registers, websites, organisations, reference lists and other sources searched or consulted to identify studies. Specify the date when each source was last searched or consulted. | Page 6 |
| Search strategy | 7 | Present the full search strategies for all databases, registers and websites, including any filters and limits used. | Supplementary material (S2) |
| Selection process | 8 | Specify the methods used to decide whether a study met the inclusion criteria of the review, including how many reviewers screened each record and each report retrieved, whether they worked independently, and if applicable, details of automation tools used in the process. | Page 6/7 |
| Data collection process | 9 | Specify the methods used to collect data from reports, including how many reviewers collected data from each report, whether they worked independently, any processes for obtaining or confirming data from study investigators, and if applicable, details of automation tools used in the process. | Page 7 |
| Data items | 10a | List and define all outcomes for which data were sought. Specify whether all results that were compatible with each outcome domain in each study were sought (e.g. for all measures, time points, analyses), and if not, the methods used to decide which results to collect. | Page 7 |
| | 10b | List and define all other variables for which data were sought (e.g. participant and intervention characteristics, funding sources). Describe any assumptions made about any missing or unclear information. | Page 7 |
| Study risk of bias assessment | 11 | Specify the methods used to assess risk of bias in the included studies, including details of the tool(s) used, how many reviewers assessed each study and whether they worked independently, and if applicable, details of automation tools used in the process. | N/A |
| Effect measures | 12 | Specify for each outcome the effect measure(s) (e.g. risk ratio, mean difference) used in the synthesis or presentation of results. | N/A |
| Synthesis methods | 13a | Describe the processes used to decide which studies were eligible for each synthesis (e.g. tabulating the study intervention characteristics and comparing against the planned groups for each synthesis (item #5)). | Page 7 |
| | 13b | Describe any methods required to prepare the data for presentation or synthesis, such as handling of missing summary statistics, or data conversions. | N/A |
| | 13c | Describe any methods used to tabulate or visually display results of individual studies and syntheses. | Page 7 |
| | 13d | Describe any methods used to synthesize results and provide a rationale for the choice(s). If meta-analysis was performed, describe the model(s), method(s) to identify the presence and extent of statistical heterogeneity, and software package(s) used. | Page 7 |
| | 13e | Describe any methods used to explore possible causes of heterogeneity among study results (e.g. subgroup analysis, meta-regression). | N/A |
| | 13f | Describe any sensitivity analyses conducted to assess robustness of the synthesized results. | N/A |
| Reporting bias assessment | 14 | Describe any methods used to assess risk of bias due to missing results in a synthesis (arising from reporting biases). | N/A |
| Certainty | 15 | Describe any methods used to assess certainty (or confidence) in the body of evidence for an outcome. | N/A |

For peer review only - http://bmjopen.bmj.com/site/about/guidelines.xhtml

# PRISMA 2020 Checklist

| Section and Topic | Item # | Checklist item | Location where item is reported |
|---|---|---|---|
| assessment | | | |
| **RESULTS** | | | |
| Study selection | 16a | Describe the results of the search and selection process, from the number of records identified in the search to the number of studies included in the review, ideally using a flow diagram. | Page 8 and Figure 1 |
| | 16b | Cite studies that might appear to meet the inclusion criteria, but which were excluded, and explain why they were excluded. | Figure 1 |
| Study characteristics | 17 | Cite each included study and present its characteristics. | Page 8/9/10/11 (Table 1) |
| Risk of bias in studies | 18 | Present assessments of risk of bias for each included study. | N/A |
| Results of individual studies | 19 | For all outcomes, present, for each study: (a) summary statistics for each group (where appropriate) and (b) an effect estimate and its precision (e.g. confidence/credible interval), ideally using structured tables or plots. | N/A |
| Results of syntheses | 20a | For each synthesis, briefly summarise the characteristics and risk of bias among contributing studies. | N/A |
| | 20b | Present results of all statistical syntheses conducted. If meta-analysis was done, present for each the summary estimate and its precision (e.g. confidence/credible interval) and measures of statistical heterogeneity. If comparing groups, describe the direction of the effect. | N/A |
| | 20c | Present results of all investigations of possible causes of heterogeneity among study results. | N/A |
| | 20d | Present results of all sensitivity analyses conducted to assess the robustness of the synthesized results. | N/A |
| Reporting biases | 21 | Present assessments of risk of bias due to missing results (arising from reporting biases) for each synthesis assessed. | N/A |
| Certainty of evidence | 22 | Present assessments of certainty (or confidence) in the body of evidence for each outcome assessed. | N/A |
| **DISCUSSION** | | | |
| Discussion | 23a | Provide a general interpretation of the results in the context of other evidence. | Page 15/16/17 |
| | 23b | Discuss any limitations of the evidence included in the review. | Page 17 |
| | 23c | Discuss any limitations of the review processes used. | Page 16/17 |
| | 23d | Discuss implications of the results for practice, policy, and future research. | Page 15/16/17 |
| **OTHER INFORMATION** | | | |
| Registration and protocol | 24a | Provide registration information for the review, including register name and registration number, or state that the review was not registered. | Page 5 |
| | 24b | Indicate where the review protocol can be accessed, or state that a protocol was not prepared. | Page 5 |
| | 24c | Describe and explain any amendments to information provided at registration or in the protocol. | N/A |
| Support | 25 | Describe sources of financial or non-financial support for the review, and the role of the funders or sponsors in the review. | Page 18 |
| Competing interests | 26 | Declare any competing interests of review authors. | Page 18 |
| Availability of data, code and other materials | 27 | Report which of the following are publicly available and where they can be found: template data collection forms; data extracted from included studies; data used for all analyses; analytic code; any other materials used in the review. | Page 7/18 |

For peer review only

# PRISMA 2020 Checklist

*From:* Page MJ, McKenzie JE, Bossuyt PM, Boutron I, Hoffmann TC, Mulrow CD, et al. The PRISMA 2020 statement: an updated guideline for reporting systematic reviews. BMJ 2021;372:n71. doi: 10.1136/bmj.n71

For more information, visit: http://www.prisma-statement.org/

For peer review only

# Developing web-based interventions to support people living with and affected by cancer: a protocol for a rapid review

Dr David Nelson[1], Dr Samuel Cooke[2], Heidi Green[2], Kathie McPeake[3], Prof. Mark Gussy[1], Dr Ros Kane[2]

[1] Lincoln International Institute for Rural Health, University of Lincoln, UK.

[2] School of Health and Social Care, University of Lincoln, UK.

[3] NHS Lincolnshire Clinical Commissioning Group

Correspondence to Dr David Nelson; dnelson@lincoln.ac.uk

## Abstract

**Introduction:** More people are living with and beyond cancer and digital interventions are increasingly being used to support them at all stages through their journey. This rapid review aims to systematically identify and explore the existing evidence that reports on primary data concerning the development of web-based interventions used to support people living with and affected by cancer.

**Methods and analysis:** Keyword searches were performed in MEDLINE to identify peer-reviewed literature on web-based interventions that are designed to support people living with and affected by cancer. The review will include studies published in the English language and will not have any restrictions on publication date or geography. Screening and data extraction will be completed independently by two reviewers. The included studies will be tabulated and the results synthesised narratively.

**Discussion:** This rapid review aims to identify and synthesise the peer-reviewed academic literature that reports on primary data concerning the development of web-based interventions to support people living with and affected by cancer. This methodology was chosen to rapidly synthesise the existing peer-reviewed evidence to support the development and design of an online web-based platform that the team are working on to make qualitative research data on lived cancer experience publicly available and accessible.

**Ethics and dissemination:** The review was registered and given a favourable ethical opinion on the 19/07/21 by a committee at the University of Lincoln (Review ref: 2021_6976). The findings from this rapid review will be presented at appropriate conferences and published in a peer reviewed academic journal as well as a report for the National Institute for Health Research Clinical Research Network.

**Systematic review registration:** The protocol was registered on the Open Science Framework [insert link here].

**Keywords:** cancer survivorship; web-based support; user experience; rapid review; protocol



For peer review only - http://bmjopen.bmj.com/site/about/guidelines.xhtml

# Introduction

Globally, cancer incidence is increasing with an estimated 18.1 million new diagnoses in 2018.[1] This can be attributed to advances in screening, earlier detection, diagnostic methods and improved treatments. Consequently, more and more people are now surviving cancer, and in the UK it is predicted that there will be four million people living with and beyond cancer by 2030.[2] Cancer raises a wide range of specific issues pertaining to information provision and emotional support [3] and there are now an increasing number of online health communities for people affected by cancer, each with their own specific aims.[4-6] Existing research has shown that people living with and affected by cancer use the internet for (1) content (online health information) (2) communication (e-mail and instant messaging) (3) communities (virtual support groups and forums) and (4) e-commerce (selling or buying products). [7]

The ongoing Covid-19 pandemic poses several challenges to oncology services and people living with and affected by cancer may now rely more heavily on digital and remote support.[8 9] Consequently, some psychosocial and supportive cancer care has now shifted from face-to-face to virtual delivery.[10] Digital health technologies have the potential to reduce health inequalities in cancer care and can improve access, integration and personalisation of care.[6] They can be particularly beneficial to those in rural and remote settings where access has long been acknowledged as a barrier to care.[11] However, the benefits of digital health technology depend partly on digital health literacy (capabilities and resources required by people to use and benefit from it).[6]

Existing reviews have previously explored the use of web-based interventions for supporting people living with and beyond cancer. These include identifying and evaluating the effectiveness of a wide range of web-based resources such as educational and psychosocial platforms[5 12 13], social media sites[14], mobile applications[14 15], and digital health interventions that focus on specific health behaviours e.g. physical activity and diet.[16] The current review will differ to previous reviews by exploring the academic evidence that reports on user and developer experience/perception for building and developing web-based tools. The evidence synthesised from the review will be used to directly inform the development of a novel web-based resource that will support those living with and beyond cancer through making qualitative research data on lived cancer experience publicly available and accessible.

This rapid review will aim to achieve the following:

- Identify and map the peer reviewed academic evidence that reports on primary data concerning the development and utilisation of web-based tools for supporting people living with and affected by cancer.

- Collate and analyse primary data with a view to informing evidence-based recommendations for the development of a novel and accessible web-based tool that meets the needs of people living with and affected by cancer.



For peer review only - http://bmjopen.bmj.com/site/about/guidelines.xhtml

## Methods

The team will utilise a rapid review approach which is now considered a key component of the knowledge synthesis family alongside systematic reviews, scoping reviews and realist reviews.[17] Despite the increase in popularity of rapid review methods there is still no universal agreement within the extant literature as to how a rapid review should be conducted or defined.[18-20] In sum, rapid reviews are a form of evidence synthesis in which components of the systematic review process are omitted or simplified with a view to producing findings in a timely manner. [18 19] Still, rapid reviews must remain systematic in their approach and have a duty to report the methods in a transparent manner making sure that they are clear about deviations or omissions from the Preferred Reporting Items for Systematic Reviews and Meta-Analyses (PRISMA) criteria.[21] This review will be conducted in line with the recently published guidance from the Cochrane Rapid Reviews Methods Group[22] and reported using the PRISMA checklist.[23]

*Protocol and Ethics*

The protocol has been registered on the Open Science Framework [insert link here]. This rapid review will be conducted over a 3-4 month period (July 2021 – October 2021).

The review was given a favourable ethical opinion by a committee at the University of Lincoln (Review ref: 2021_6976) on 19/07/21.

*Search Strategy*

We used keyword searches together with Boolean operators (OR and AND) and truncation (*) to locate relevant peer-reviewed literature on the user experience of web-based support that is delivered to people living with and affected by cancer. We searched MEDLINE (20/07/21) which is the leading full-text database of biomedical and health journals. The primary search strategy and syntax was developed and refined by three members of the review team (DN, SC, HG). The search strategy for MEDLINE can be found at Appendix 1. Due to time constraints, limited sources and the need to produce findings in a timely manner, we are limiting our search to one database which is considered acceptable for a rapid systematic review.[18 19] All database searches were supplemented with google scholar searches in addition with forward and backward citation searchers of relevant articles. Database searches will continually be updated to identify and incorporate the most up to date evidence where appropriate.

To identify people living with and affected by cancer we will use the following keywords: "cancer surviv*" or "living with cancer" or "living with and beyond cancer" or "cancer patient*" or "patients with cancer" "people affected by cancer" or "oncology patient" or "cancer experience*" or "cancer management" or "cancer support" or "cancer care*"

The following keywords will be used to identify web-based support and interventions: "web*" or "internet*" or "online*" or "digital*"

To search literature on user experience we will use the following keywords: "user experience*" or "usability" or "functionality" or "design" or "interaction" or "development" or "user testing"



For peer review only - http://bmjopen.bmj.com/site/about/guidelines.xhtml

*Inclusion and Exclusion Criteria*

Peer-reviewed publications will be selected for inclusion in the review utilising the pre-defined eligibility criteria outlined in Table 1 below.

**Table 1 Inclusion and Exclusion Criteria**

| **Criterion** | **Include** | **Exclude** |
|---|---|---|
| Population | Adults (18+)<br>All genders<br>People living with cancer/caregivers/people affected by cancer<br>Any geography. | Non-adult populations (under 18). |
| Intervention | Website based/internet-based cancer support resources. | Support programmes that focus solely on mobile and digital apps.<br><br>E-learning programmes or interventions (self-directed and practitioner/professionally lead).<br><br>Social media/networking sites |
| Comparator | Not applicable. | Not applicable. |
| Outcomes | Reports primary data on user and developer experience/usability/functionality/design on web/internet-based support for people living with and affected by cancer. | There are no primary data reported on user experience/usability/functionality/design on web/internet-based support for people living with and affected by cancer. |
| Study design | Reports empirical research data using the following designs:<br>• Quantitative<br>• Qualitative<br>• Mixed Methods | • Systematic and literature reviews<br>• Editorials<br>• Commentaries<br>• Opinion pieces<br>• Case series or reports |
| Language | Published in the English language. | Not published in the English language. |

## Data and Analysis

*Record Selection*

References identified via the search were exported and managed using Endnote Version X9. The final search identified 2,452 articles for screening, see Appendix 1. The titles and abstracts will be independently screened against the eligibility criteria by two reviewers (DN and SC). Where discrepancies exist, the team will aim to resolve via discussion or through a third reviewer (HG). Following title and abstract screening, the remaining articles will be independently screened by full text, for inclusion by two reviewers (DN and SC), with any disagreements resolved through discussion or a third reviewer (RK). The results will be presented in a PRISMA flow diagram.

*Data Extraction*

Data will be extracted using an adapted Cochrane Data Extraction Template and this can be found at Appendix 2. This will be piloted with a subset ($n$=5) of full text studies that meet our eligibility criteria to determine whether any further changes are needed to the data extraction



For peer review only - http://bmjopen.bmj.com/site/about/guidelines.xhtml

template. After piloting the tool, two reviewers (DN and SC) will undertake data extraction for each full text article with cross checking for data quality taking place by a third reviewer (HG). Study characteristics will be extracted from each study based upon (1) study methods (e.g. aims/objectives, study design, participants, outcomes) (2) details on the web-based intervention/support and (3) study findings (details of all relevant data concerning user experience, usability, functionality, and design). All discrepancies will be resolved through further discussion, or where required, a fourth reviewer (RK).

*Data synthesis and analysis*

It is likely that the review will include a wide range of study designs that make use of both quantitative and qualitative methodologies. Following data extraction, the results of the full text articles will be tabulated. Quantitative data will be described using basic descriptive statistics as well as being written up narratively. We do not plan to conduct a formal statistical meta-analysis. Qualitative findings on user experience will be analysed using thematic synthesis.[24] Where possible we will group and comment on similarities and dissimilarities within the user experience data.

*Quality assessment*

The focus of this rapid review is on identifying and exploring the literature on user experience of web-based support that is delivered to people living with and beyond cancer, therefore, a quality assessment of included articles was not deemed appropriate.

## Discussion

The aim of this research is to identify and map the peer reviewed academic evidence that reports on primary data concerning the development of web-based tools that support people living with and affected by cancer. It will also aim to collate and analyse data with a view to informing evidence-based recommendations for the development of a novel and accessible web-based tool that meets the needs of people living with and affected by cancer. It has been acknowledged that digital interventions have the potential to provide an excellent source of support for people living with and affected by cancer.[5 25] Specifically, they can help people to cope better with the disease and with side effects as well as improving self-management and wellbeing.[5]

A rapid review methodology was chosen to support and inform the timely need for the development and implementation of an innovative online web-based platform that is informed by peer-reviewed academic evidence. Rapid reviews are useful in adapting to and overcoming time and resource constraints that genuinely prevent the development and execution of a high-quality systematic review.[22] Nevertheless, researchers must endeavour to optimise the methodological rigour, clarity, and reproducibility of a rapid review, starting with the implementation of a rigorous and systematic protocol.[22]

Systematic reviews and meta-analyses are often intensive[26], especially when considering the large number of included studies, which is likely to be the case for evidence concerning user experience for developing web-based interventions in cancer. Whilst conducting a rapid review may accelerate the development process, it will enable a timely evidence appraisal that will address priority research questions as well as allowing for the rapid dissemination of findings.



For peer review only - http://bmjopen.bmj.com/site/about/guidelines.xhtml

*Dissemination*

This rapid review and wider work (development of a web-based platform to support people living with and affected by cancer) benefits from the establishment of a study steering group with representation from academic researchers, cancer professionals and people with lived cancer experience. The review team will draw on their expertise and the findings will be disseminated in accordance with an ongoing dissemination strategy that will be developed collaboratively by the review team and steering group. This will involve presenting at appropriate local and national conferences, as well as, publishing in a peer reviewed academic journal. A summary of the findings will also be written up as a report for the National Institute for Health Research (NIHR) Clinical Research Network (CRN) who are funding this work. The team will disseminate the results in lay and accessible formats including using social media and press releases via the University of Lincoln and Macmillan Cancer Support.

## Funding

This review was funded by the National Institute for Health Research (NIHR) Clinical Research Network (CRN).

## Acknowledgements

We would like to acknowledge the Steering Group for their support in directing this review as well as the wider project. Acknowledgments must also go to Macmillan Cancer Support and the East Midlands Cancer Alliance for previous funding and their continued support throughout the early development of the Shared Lives Cancer project.

## References

1. Bray F, Ferlay J, Soerjomataram I, et al. Global cancer statistics 2018: GLOBOCAN estimates of incidence and mortality worldwide for 36 cancers in 185 countries. *CA: A Cancer Journal for Clinicians* 2018;68(6):394-424. doi: https://doi.org/10.3322/caac.21492
2. Maddams J, Utley M, Møller H. Projections of cancer prevalence in the United Kingdom, 2010–2040. *British Journal of Cancer* 2012;107(7):1195-202. doi: 10.1038/bjc.2012.366
3. van Eenbergen MC, van de Poll-Franse LV, Heine P, et al. The Impact of Participation in Online Cancer Communities on Patient Reported Outcomes: Systematic Review. *JMIR Cancer* 2017;3(2):e15. doi: 10.2196/cancer.7312
4. van Eenbergen MC, van de Poll-Franse LV, Krahmer E, et al. Analysis of Content Shared in Online Cancer Communities: Systematic Review. *JMIR Cancer* 2018;4(1):e6. doi: 10.2196/cancer.7926
5. Escriva Boulley G, Leroy T, Bernetière C, et al. Digital health interventions to help living with cancer: A systematic review of participants' engagement and psychosocial effects. *Psycho-Oncology* 2018;27(12):2677-86. doi: https://doi.org/10.1002/pon.4867
6. Kemp E, Trigg J, Beatty L, et al. Health literacy, digital health literacy and the implementation of digital health technologies in cancer care: the need for a strategic approach. *Health Promot J Austr* 2021;32 Suppl 1:104-14. doi: 10.1002/hpja.387
7. Eysenbach G. The Impact of the Internet on Cancer Outcomes. *CA: A Cancer Journal for Clinicians* 2003;53(6):356-71. doi: https://doi.org/10.3322/canjclin.53.6.356
8. Al-Quteimat OM, Amer AM. The Impact of the COVID-19 Pandemic on Cancer Patients. *Am J Clin Oncol* 2020;43(6):452-55. doi: 10.1097/COC.0000000000000712
9. Burki TK. Cancer guidelines during the COVID-19 pandemic. *The Lancet Oncology* 2020;21(5):629-30. doi: 10.1016/S1470-2045(20)30217-5
10. Bultz BD, Watson L. Lessons learned about virtual cancer care and distress screening in the time of COVID-19. *Supportive Care in Cancer* 2021 doi: 10.1007/s00520-021-06322-6



For peer review only - http://bmjopen.bmj.com/site/about/guidelines.xhtml

11. Douthit N, Kiv S, Dwolatzky T, et al. Exposing some important barriers to health care access in the rural USA. *Public Health* 2015;129(6):611-20. doi: https://doi.org/10.1016/j.puhe.2015.04.001
12. Aapro M, Bossi P, Dasari A, et al. Digital health for optimal supportive care in oncology: benefits, limits, and future perspectives. *Support Care Cancer* 2020;28(10):4589-612. doi: 10.1007/s00520-020-05539-1 [published Online First: 2020/06/14]
13. McCann L, McMillan KA, Pugh G. Digital Interventions to Support Adolescents and Young Adults With Cancer: Systematic Review. *JMIR Cancer* 2019;5(2):e12071. doi: 10.2196/12071 [published Online First: 2019/08/02]
14. Charbonneau DH, Hightower S, Katz A, et al. Smartphone apps for cancer: A content analysis of the digital health marketplace. *DIGITAL HEALTH* 2020;6:2055207620905413. doi: 10.1177/2055207620905413
15. Prochaska JJ, Coughlin SS, Lyons EJ. Social Media and Mobile Technology for Cancer Prevention and Treatment. *Am Soc Clin Oncol Educ Book* 2017;37:128-37. doi: 10.1200/edbk_173841 [published Online First: 2017/06/01]
16. Roberts AL, Fisher A, Smith L, et al. Digital health behaviour change interventions targeting physical activity and diet in cancer survivors: a systematic review and meta-analysis. *J Cancer Surviv* 2017;11(6):704-19. doi: 10.1007/s11764-017-0632-1 [published Online First: 2017/08/06]
17. Moher D, Stewart L, Shekelle P. All in the Family: systematic reviews, rapid reviews, scoping reviews, realist reviews, and more. *Systematic Reviews* 2015;4(1):183. doi: 10.1186/s13643-015-0163-7
18. Khangura S, Konnyu K, Cushman R, et al. Evidence summaries: the evolution of a rapid review approach. *Systematic Reviews* 2012;1(1):10. doi: 10.1186/2046-4053-1-10
19. Tricco AC, Antony J, Zarin W, et al. A scoping review of rapid review methods. *BMC Medicine* 2015;13(1):224. doi: 10.1186/s12916-015-0465-6
20. Kelly SE, Moher D, Clifford TJ. DEFINING RAPID REVIEWS: A MODIFIED DELPHI CONSENSUS APPROACH. *International Journal of Technology Assessment in Health Care* 2016;32(4):265-75. doi: 10.1017/S0266462316000489 [published Online First: 2016/10/11]
21. Schünemann HJ, Moja L. Reviews: Rapid! Rapid! Rapid! …and systematic. *Systematic Reviews* 2015;4(1):4. doi: 10.1186/2046-4053-4-4
22. Garritty C, Gartlehner G, Nussbaumer-Streit B, et al. Cochrane Rapid Reviews Methods Group offers evidence-informed guidance to conduct rapid reviews. *Journal of Clinical Epidemiology* 2021;130:13-22. doi: https://doi.org/10.1016/j.jclinepi.2020.10.007
23. Moher D, Liberati A, Tetzlaff J, et al. Preferred Reporting Items for Systematic Reviews and Meta-Analyses: The PRISMA Statement. *PLOS Medicine* 2009;6(7):e1000097. doi: 10.1371/journal.pmed.1000097
24. Thomas J, Harden A. Methods for the thematic synthesis of qualitative research in systematic reviews. *BMC Medical Research Methodology* 2008;8(1):45. doi: 10.1186/1471-2288-8-45
25. Laing CM, Moules NJ, Sinclair S, et al. Digital Storytelling as a Psychosocial Tool for Adult Cancer Survivors. *Oncol Nurs Forum* 2019;46(2):147-54. doi: 10.1188/19.onf.147-154
26. Allen IE, Olkin I. Estimating time to conduct a meta-analysis from number of citations retrieved. *Jama* 1999;282(7):634-5. doi: 10.1001/jama.282.7.634 [published Online First: 1999/10/12]



For peer review only - http://bmjopen.bmj.com/site/about/guidelines.xhtml

**Appendix 1 Search Strategy for MEDLINE**

| Key search terms | Date | Hits (*n*=) | Parameters |
|---|---|---|---|
| S1: "Cancer surviv*" | 20/07/21 | 27,751 | Medline only |
| S2: "Living with cancer" | 20/07/21 | 865 | Medline only |
| S3: "Living with and beyond cancer" | 20/07/21 | 103 | Medline only |
| S4: "Cancer patient*" | 20/07/21 | 203,503 | Medline only |
| S5: "Patients with cancer" | 20/07/21 | 31,724 | Medline only |
| S6: "People affected by cancer" | 20/07/21 | 146 | Medline only |
| S7: "Oncology patient*" | 20/07/21 | 6,036 | Medline only |
| S8: "Cancer experience*" | 20/07/21 | 2,911 | Medline only |
| S9: "Cancer management" | 20/07/21 | 4,928 | Medline only |
| S10: "Cancer support" | 20/07/21 | 1,350 | Medline only |
| S11: "Cancer care*" | 20/07/21 | 26,976 | Medline only |
| **S12: S1 OR S2 OR S3 OR S4 OR S5 OR S6 OR S7 OR S8 OR S9 OR S10 OR S11** | **20/07/21** | **280,235** | **Medline only** |
| S13: "Web*" | 20/07/21 | 287,832 | Medline only |
| S14: "Internet*" | 20/07/21 | 113,871 | Medline only |
| S15: "Online*" | 20/07/21 | 150,659 | Medline only |
| S16: "Digital*" | 20/07/21 | 182,713 | Medline only |
| **S17: S13 OR S14 OR S15 OR S16** | **20/07/21** | **650,510** | **Medline only** |
| S18: "User experience*" | 20/07/21 | 2,428 | Medline only |
| S19: "Usability" | 20/07/21 | 15,671 | Medline only |
| S20: "Functionality" | 20/07/21 | 66,353 | Medline only |
| S21: "Design" | 20/07/21 | 1,135,342 | Medline only |
| S22: "Interaction" | 20/07/21 | 876,907 | Medline only |
| S23: "Development" | 20/07/21 | 3,148,188 | Medline only |
| S24: "User testing" | 20/07/21 | 350 | Medline only |
| **S25: S18 OR S19 OR S20 OR S21 OR S22 OR S23 OR S24** | **20/07/21** | **4,858,310** | **Medline only** |
| **S26: S12 AND S17 AND S25** | **02/08/21** | **2,452** | **Medline only** |

For peer review only

### Appendix 2 Adapted Data Extraction Form

| | |
|---|---|
| Review title or ID | |
| Study ID *(surname of first author and year first full report of study was published e.g. Smith 2001)* | |
| Report ID | |
| Report ID of other reports of this study including errata or retractions | |
| Notes | |

## General Information

| | |
|---|---|
| Date form completed *(dd/mm/yyyy)* | |
| Name/ID of person extracting data | |
| Reference citation | |
| Study author contact details | |
| Publication type *(e.g. full report, abstract, letter)* | |
| Notes: | |

## Study eligibility

| **Study Characteristics** | **Eligibility criteria** *(Insert inclusion criteria for each characteristic as defined in the Protocol)* | **Eligibility criteria met?** Yes / No / Unclear | **Location in text or source** *(pg & ¶/fig/table/other)* |
|---|---|---|---|
| **Type of study** | Quantitative design | ☐ ☐ ☐ | |
| | Qualitative design | ☐ ☐ ☐ | |
| | Mixed methods design | ☐ ☐ ☐ | |
| | Other (Please specify) | | |
| **Participants** | Cancer (Specify what type) | ☐ ☐ ☐ | |
| | Caregiver | ☐ ☐ ☐ | |
| | Friends/family | ☐ ☐ ☐ | |
| **Types of intervention** | Website/Internet-based cancer support programme | ☐ ☐ ☐ | |



For peer review only - http://bmjopen.bmj.com/site/about/guidelines.xhtml

| | | | | | |
|---|---|---|---|---|---|
| **Type of data (quant and/or qual)**<br><br>(Reports primary data on any of the following) | User experience | ☐ | ☐ | ☐ | |
| | Usability | ☐ | ☐ | ☐ | |
| | Functionality | ☐ | ☐ | ☐ | |
| | Design on web | ☐ | ☐ | ☐ | |
| | Internet-based support living with and affected by cancer. | ☐ | ☐ | ☐ | |
| INCLUDE ☐ | | EXCLUDE ☐ | | | |
| Reason for exclusion | | | | | |
| Notes: | | | | | |

**DO NOT PROCEED IF STUDY EXCLUDED FROM REVIEW**

## Characteristics of included studies

### Methods

| | **Descriptions as stated in report/paper** | **Location in text or source** *(pg & ¶/fig/table/other)* |
|---|---|---|
| **Aim of study** *(e.g. efficacy, equivalence, pragmatic)* | | |
| **Participants** *(e.g. Type of cancer, caregiver role, family and friend's role)* | | |
| **Design** *(e.g. parallel, crossover, non-RCT, exploratory)* | | |



For peer review only - http://bmjopen.bmj.com/site/about/guidelines.xhtml

| | | |
|---|---|---|
| **Outcomes** *(details of primary data e.g. user experience, usability, functionality, design etc )* | | |
| **Start/End date** | | |
| **Ethical approval needed/ obtained for study** | ☐ Yes ☐ No ☐ Unclear | | |
| **Notes:** | | |

For peer review only



For peer review only - http://bmjopen.bmj.com/site/about/guidelines.xhtml

| | |
|---|---|
| Review title or ID | Hodgkin lymphoma survivor wellness: Development of a web-based intervention. |
| Study ID *(surname of first author and year first full report of study was published e.g. Smith 2001)* | Amweg et al. (2020) |
| Report ID | 10.1188/20.CJON.284-289 |
| Report ID of other reports of this study including errata or retractions | N/A |
| Notes: N/A | |

## General Information

| | |
|---|---|
| Date form completed *(dd/mm/yyyy)* | 12/10/2021 |
| Name/ID of person extracting data | SC |
| Reference citation | Amweg LN, McReynolds J, Lansang K, Jones T, Snow C, Berry DL, Partridge AH, Underhill-Blazey ML. Hodgkin Lymphoma Survivor Wellness: Development of a Web-Based Intervention. Clin J Oncol Nurs. 2020 Jun 1;24(3):284-289. doi: 10.1188/20.CJON.284-289. PMID: 32441674. |
| Study author contact details | Could not access |
| Publication type *(e.g. full report, abstract, letter)* | Full report |
| Notes: N/A | |

## Study eligibility

| **Study Characteristics** | **Eligibility criteria** *(Insert inclusion criteria for each characteristic as defined in the Protocol)* | **Eligibility criteria met?** **Yes** | **No** | **Unclear** | **Location in text or source** *(pg & ¶/fig/table/other)* |
|---|---|---|---|---|---|
| **Type of study** | Quantitative design | ☐ | ☐ | ☐ | |
| | Qualitative design | ☐ | ☐ | ☐ | |
| | Mixed methods design | ☒ | ☐ | ☐ | Page 1 – Abstract<br>Page 2 – methods – Focus groups – Development and usability testing |
| | Other (Please specify) | | | | |

For peer review only

| | | | | | |
|---|---|---|---|---|---|
| **Participants** | Cancer (Specify what type) | ☒ | ☐ | ☐ | Page 2 – Participant identification and recruitment |
| | Caregiver | ☐ | ☐ | ☐ | |
| | Friends/family | ☐ | ☐ | ☐ | |
| **Types of intervention** | Website/Internet-based cancer support programme | ☒ | ☐ | ☐ | Page 2 – Design and setting |

| | | | | | |
|---|---|---|---|---|---|
| **Type of data (quant and/or qual)**<br>(Reports primary data on any of the following) | User experience/Needs/Preferences | ☒ | ☐ | ☐ | Page 2 – Focus groups |
| | Usability/Acceptability/Feasibility | ☒ | ☐ | ☐ | Page 2 – Development and individual usability testing |
| | Functionality | ☐ | ☐ | ☐ | |
| | Design on web | ☐ | ☐ | ☐ | |
| | Internet-based support living with and affected by cancer. | ☐ | ☐ | ☐ | |
| INCLUDE ☒ | | EXCLUDE ☐ | | | |
| Reason for exclusion | | | | | |

Notes:

The aim of this article was to adapt and evaluate a previously developed survivorship care website for Hodgkin Lymphoma survivors.

A mixed methods design broken down into phases

1) Focus group interviews – user needs
2) Web design and user testing – usability

**DO NOT PROCEED IF STUDY EXCLUDED FROM REVIEW**

For peer review only

# Characteristics of included studies

## Methods

| | Descriptions as stated in report/paper | | | | Location in text or source *(pg & ¶/fig/table/other)* |
|---|---|---|---|---|---|
| **Aim of study** *(e.g. efficacy, equivalence, pragmatic)* | The aim of this article was to adapt and evaluate a previously developed survivorship care website for Hodgkin Lymphoma survivors. | | | | Page 1 – Abstract<br><br>Page 2 – Introduction – paragraph 2 |
| **Participants** *(e.g. Type of cancer, caregiver role, family and friend's role)* | Hodgekins Lymphoma survivors | | | | Page 2 – Methods – Participant identification and recruitment |
| **Design** *(e.g. parallel, crossover, non-RCT, exploratory)* | Mixed methods user centred design – two phases<br><br>1) Focus groups<br>2) Development and individual usability testing | | | | Page 2 – Methods – Focus groups – Development and usability testing |
| **Outcomes** *(details of primary data e.g. user experience, usability, functionality, design etc )* | User needs/preferences and usability of web-resource | | | | Page 2 – Methods – Focus groups – Development and usability testing |
| **Start/End date** | | | | | |
| **Ethical approval needed/ obtained for study** | ☒ Yes | ☐ No | ☐ Unclear | The Dana-Farber/Harvard Cancer Centre Institutional Review Board approved all study procedures. The study was conducted from April 2017 through December 2018. | Page 2 – Methods – Design and setting. |

For peer review only

**Notes:**

For peer review only
